# *MYC* Is an Early Response Regulator of Human Adipogenesis in Adipose Stem Cells

**DOI:** 10.1371/journal.pone.0114133

**Published:** 2014-12-01

**Authors:** Chad Deisenroth, Michael B. Black, Salil Pendse, Linda Pluta, Sam M. Witherspoon, Patrick D. McMullen, Russell S. Thomas

**Affiliations:** 1 The Hamner Institutes for Health Sciences, Institute for Chemical Safety Sciences, Research Triangle Park, North Carolina, United States of America; 2 Biomanufacturing Research Institute and Technology Enterprise, North Carolina Central University, Durham, North Carolina, United States of America; Wake Forest Institute for Regenerative Medicine, United States of America

## Abstract

Adipose stem cell (ASC) differentiation is necessary for the proper maintenance and function of adipose tissue. The procurement and characterization of multipotent ASCs has enabled investigation into the molecular determinants driving human adipogenesis. Here, the transcription factor *MYC* was identified as a significant regulator of ASC differentiation. Expression of *MYC* transcript and protein was found to accumulate during the initial course of differentiation. Loss-of-function analysis using siRNA mediated knockdown of *MYC* demonstrated inhibition of hormonally stimulated adipogenesis. *MYC* exhibited an early and sustained expression pattern that preceded down regulation of key suppressor genes, as well as induction of transcriptional and functional effectors. Glucocorticoid stimulation was identified as a necessary component for *MYC* induction and was found to impact adipogenesis in a concentration-dependent manner. Global gene expression analysis of *MYC* knockdown in ASC enriched for functional pathways related to cell adhesion, cytoskeletal remodeling, and transcriptional components of adipogenesis. These results identify a functional role for *MYC* in promotion of multipotent ASC to the adipogenic lineage.

## Introduction

Human adipose stem cells (ASC) are derived from the stromal vascular fraction of subcutaneous white adipose tissue. Like bone marrow-derived mesenchymal stem cells, ASC are multipotent, fibroblast-like cells of mesoderm lineage with the capacity to differentiate into multiple lineages with directed stimuli [1]. In adult adipose tissue, adipocytes turnover at a rate of ∼10% of cells per year in order to maintain balance between cell death and renewal [2]. The *in vivo* dynamics of adipogenesis are relatively unknown, but may involve recruitment of ASC from a perivascular stem cell niche to the location of terminal differentiation [3]. Maturation of ASC is encompassed by initial commitment to an adipogenic lineage, followed by the coordinated execution of morphological, biochemical, and transcriptional changes that are required to promote a terminal lineage fate [4]. While the majority of molecular determinants driving adipogenesis have been identified *in vitro* using mouse committed preadipocyte models such as 3T3-L1 and 3T3-F442A, the procurement of primary human ASC has facilitated investigation into the regulatory components that direct ASC lineage commitment and terminal differentiation.

The transcription factor MYC is a multi-functional protein implicated in a broad range of cellular functions including cell growth, proliferation, metabolism, apoptosis, and differentiation [5]. Stimulation by a variety of hormones and cytokines can promote stabilization of MYC protein levels to enhance subsequent nuclear transactivation of MYC dependent target genes. While the function of MYC has been well studied in the context of cancer cell growth and proliferation, the role of MYC in cellular differentiation has been less clear. Ectopic expression of *MYC* has been reported to inhibit differentiation of a number of cell types, including preadipocyte models [6],[7],[8],[9],[10],[11],[12],[13]. For instance, over expression of *MYC* in 3T3-L1 committed preadipocytes facilitates normal expression of early response regulators *CEBPB* and *CEBPD* during the course of differentiation, but attenuates induction of *CEBPA* and *PPARG* to inhibit terminal adipocyte maturation [12]. These effects are suggested to be independent of cell cycle progression that occurs in response to adipogenic stimulus during mitotic clonal expansion in the murine 3T3-L1 and 3T3-F442A models [13],[14]. Such findings are in contrast to more recent observations in a number of cellular systems where *MYC* is essential for proper tissue development [15],[16],[17]. In primary epidermal stem cells, MYC expression promotes exit from the stem cell compartment to aid in terminal differentiation [18]. Indeed, deregulation of *MYC* depletes the epidermal stem cell niche by promoting transient mobilization and migration of cells to sites of terminal differentiation [19],[20] in a manner that involves modulation of cell adhesion, motility, and extracellular matrix (ECM) components [21]. Interestingly, similar effects are observed for hematopoietic stem cells where *MYC* maintains the balance between hematopoietic stem cell self-renewal and differentiation by regulating compartmentalization within the stem cell niche via regulation of cell-ECM interactions [17]. Taken together, regulation of endogenous *MYC* during biologically-defined differentiation programs suggests that *MYC* may exert a positive influence on determination of adipose stem cell fate.

Using ASC as a human relevant model, *MYC* was identified as a critical regulator of adipogenesis. Loss-of-function analysis of *MYC* yielded a functional phenotype of reduced lipid accumulation in two independent donor pools of human subcutaneous ASC. Reduced *MYC* expression also correlated with attenuated expression of terminal adipogenic markers both at the protein and transcript level. Time course gene expression measurements showed that *MYC* expression was an early event following adipogenic stimulation. Microarray analysis of *MYC* knockdown samples points to pathways affecting adipogenesis such as cell adhesion, cytoskeletal remodeling, and key genes implicated in transcription-mediated adipogenic programming. Expression of *MYC* was also observed to be glucocorticoid-dependent. The cumulative data suggest *MYC* is essential for adipogenesis in human multipotent adipose stem cells.

## Materials and Methods

### Cell Culture and Reagents

Human subcutaneous adipose stem cells derived from pooled donor superlots (SL0044 and SL0048, Zen-Bio, Research Triangle Park, NC) were obtained at passage 2–3 and used for all experiments. SL0044 was obtained from six independent, non-diabetic, non-smoker, Caucasian female donors, with mean age of 41.3 [range: 36–45] and mean Body Mass Index of 28.9 [range: 28.1–29.8] (Table 1). SL0048 was obtained from a range of anatomical sites of eight Caucasian female donors with a mean age of 44 [range: 29–51] and mean BMI of 26.3 [range: 25.1–29.2] (Table 2). Cells were maintained in Dulbecco's Modified Eagle's Medium (Thermo Scientific, Waltham, MA) supplemented with 10% fetal bovine serum (Sigma, St. Louis, MO), L-glutamine, 100 U/ml penicillin and 100 µg/ml streptomycin at 5% CO2 in a 37°C humidified chamber. Antibodies used were c-Myc (N-262) (sc-764, Santa Cruz Biotechnology, Dallas, TX), PPARγ (H-100) (sc-7196, Santa Cruz Biotechnology), A-FABP (C-15) (sc-18661, Santa Cruz Biotechnology), and GAPDH (ab9483, Abcam, Cambridge, MA).

**Table 1:** Demographic information for white subcutaneous fat tissue derived SL0044 adipose stem cells.

SL0044 Adipose Stem Cell Characteristics							
Donors	Gender	Mean Age (years)	Mean BMI	Location	Diabetic	Ethnicity	Smoker
6	Female	41.3 [range: 36–45]	28.9 [range: 28.1–29.8]	Abdomen	No	Caucasian	No

**Table 2:** Demographic information for mixed depot white subcutaneous fat tissue derived SL0048 adipose stem cells.

SL0048 Adipose Stem Cell Characteristics							
Donors	Gender	Mean Age (years)	Mean BMI	Location	Diabetic	Ethnicity	Smoker
8	Female	44 [range: 29–51]	26.3 [range: 25.1–29.2]	Abdomen, Breast, Flank, Hip, Knee, Thigh	No	Caucasian	No

### Adipocyte Differentiation Assay

Adipose stem cells were seeded at a density of 40,625 cells/cm^2^. Cells were maintained in maintenance media for 24 hours post-seeding prior to adding differentiation media. For differentiation, maintenance media was supplemented with 10 µg/ml human insulin (Sigma, St. Louis, MO), 200 µM Isobutylmethylxanthine (Sigma, St. Louis, MO), 20 nM Dexamethasone (Sigma, St. Louis, MO), and 1 µM Rosiglitazone (Cayman Chemical, Ann Arbor, MI) for 7 days. Maintenance media supplemented with 10 µg/ml of human insulin was used to replenish media after 7 days.

### siRNA

For assessment of individual gene knockdown, cells were seeded at 7,500 cells/well in 96-well plates and transfected with 25 nM siRNA duplexes (Dharmacon/Thermo Scientific, Waltham, MA) using Lipofectamine RNAiMax (Life Technologies, Carlsbad, CA). Seventy-two hours post-transfection, cells were initiated to differentiate. For endpoint analysis, neutral lipid accumulation was determined by the fluorescence based AdipoRed assay (Lonza, Basel, Switzerland) and data acquired on a Spectramax M3 plate reader (Molecular Devices, Sunnyvale, CA). Lipid accumulation was normalized to relative cell number based on Hoechst 33342 (Sigma, St. Louis, MO) nuclear stain intensity.

For *MYC* siRNA experiments, the following oligos were used: ON-TARGETplus Non-targeting Pool (Thermo; D-001810-10-05), ON-TARGETplus SMARTpool Human PPARG (Thermo; L-003436-00-0005), siGENOME SMART pool Human MYC (Thermo; M-003282-07-0005), siGENOME Human MYC siRNA-Set of 4 (Thermo; MU-020826-01-0002), Silencer Select Human MYC (Ambion; s9129 and s9130, Life Technologies, Calrsbad, CA).

### Quantitative RT-PCR (qRT-PCR)

For qRT-PCR analysis, the following TaqMan probes (Life Technologies, Carlsbad, CA) were used: Hs00234592_m1 (hPPARG), Hs03929097_g1 (hGAPDH), Hs00153408_m1 (hMYC), Hs00160173_m1 (hPLIN1), Hs00269972_s1 (hCEBPA), Hs01086177_m1 (hFABP4), Hs00998133_m1 (hTGFB1), Hs00608224_m1 (hWNT2). Data was collected on an ABI 7900HT thermal cycler and normalized to GAPDH to assess relative gene expression based on the 2(-ΔΔC(T)) method [22].

### Microarray Analysis

ASC were seeded at 28,000 cells/cm^2^ in 6-well plates and transfected with *MYC* siRNA for 72 hours to enable reduction of MYC protein levels and allow the cells to reach confluence. Differentiation was initiated with adipogenic cocktail and maintained for an additional 72 hours. Total RNA was extracted with the Qiagen RNeasy mini kit (Qiagen, Valencia, CA) and quantitated on a NanoDrop spectrophotometer (NanoDrop Tech, Wilmington, DE). A total of 5 µg of RNA was used to generate cDNA with the one-cycle cDNA synthesis kit (Affymetrix Corp., Santa Clara, CA). cRNA generated from the cDNA was biotin labeled with the GeneChip IVT labeling kit (Affymetric Corp.). Fifteen µg of labeled cRNA was hybridized to an Affymetrix Human Genome U133 Plus PM Array for 16 h at 45°C and subsequently washed on a GeneChip Fluidics Station 450. Arrays were scanned on a GeneTitan to generate CEL files with corresponding intensity values. A total of three experimental replicates (n = 3) of the SL0044 donor pool were analyzed for each treatment group. Gene expression data was deposited in Gene Expression Omnibus and is publicly accessible (GEO: GSE57538).

For expression analysis, Affymetrix CEL files were normalized by RMA [23] using the BioConductor (release 2.13) package *affy* [24], log_2_ transformed, and analyzed with the package LIMMA [25]. A single linear model was fit by robust regression with sufficient iterations to ensure the model achieved convergence. Statistical significance was determined using Empirical Bayesian statistics, with separate false discovery rate [26] corrections for each contrast in the linear model design matrix.

### Gene Pathway Analysis

Overrepresented pathways in the up- and down regulated genes were identified using the GeneGo MetaCore minibase v6.14.61644_4580 (Thompson Reuters: http://lsresearch.thomsonreuters.com). To filter for robust responses, only genes that were 1.5-fold up- or down regulated were used for pathway analysis. Pathway Maps overrepresented for differentially expressed genes were determined using a hypergeometric test with the Benjamini-Hochberg false discovery rate controlling procedure. Enrichment of pathways was tested for up- and down regulated genes separately. Categories with a false discovery rate-corrected p≤0.005 were considered significantly enriched.

A network of the relationships between Pathway Maps was created using the Cytoscape javascript library (http://cytoscape.github.io/cytoscape.js), as described previously [27]. The node size reflects the number of genes in the category that are differentially expressed relative to the total number of genes in the pathway. The degree of saturation (red for up regulated; blue for down regulated) reflects the FDR-corrected p-value. Only pathways significantly enriched for up- or down regulated genes are shown.

### Statistical Analysis

GraphPad Prism V6.0 (GraphPad Software, La Jolla, CA) was used to conduct statistical tests. Specific statistical analyses for each experiment are described within the figure legends.

## Results

### *MYC* is induced during adipogenesis

**Figure 1:**
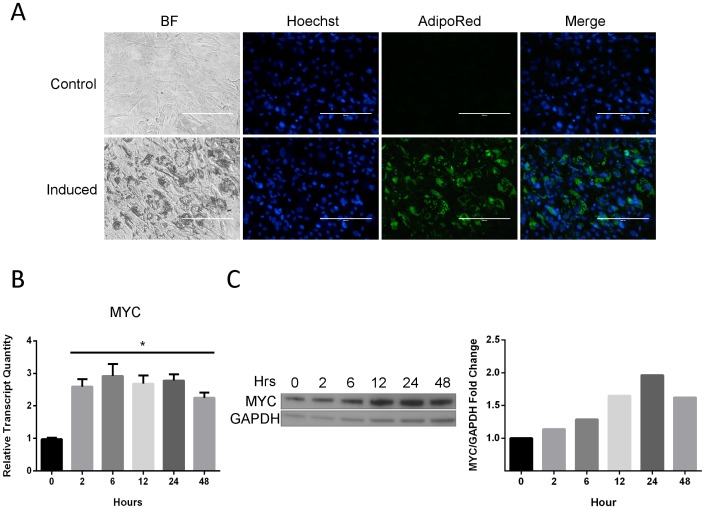
*MYC* transcript and protein levels are induced during adipocyte differentiation. (A) ASC exposed to induction cocktail (Induced) or left untreated (Control) for 7 days. BF: Bright-Field, Hoechst: Nuclear marker, AdipoRed: Neutral lipid marker. Micrographs are at 20X magnification. (B) Relative quantity (RQ) of *MYC* transcript levels in ASC following adipogenic stimulation at 2, 6, 12, 24, and 48 hours. One-way ANOVA with Dunnett posttest was used to calculate significance relative to non-induced (0 hour) control * p<0.001. Bars represent the mean ± SD of 3 experimental replicates. (C) MYC protein levels were determined at the same time points. GAPDH was used as a loading control. Relative expression on Western blots was quantitated and normalized to GAPDH. Fold-change values determined relative to non-induced (0 hour) control. The blot is a representative of 3 experimental replicates.

Primary human ASC were used as a model to systematically investigate the function of the transcription factor MYC during human adipogenesis. The initial ASC population used in this study was derived from six Caucasian female donors, with mean age of 41.3 [range: 36–45] and mean Body Mass Index of 28.9 [range: 28.1–29.8] (Table 1). ASC were positive for cell surface markers CD105 and CD44, and negative for CD31 and CD45. To validate an induction protocol and adipogenic potential of the human-derived ASC population, a cocktail of insulin, dexamethasone, isobutylmethylxanthine (IBMX), and the PPARG agonist rosiglitazone was applied for a period of 7 days to early passage ASC plated out at high density. The induced population exhibited significant peri-nuclear lipid droplet formation, adopted a more rounded morphology, and stained positive for neutral lipid stain (Fig. 1A).To assess any fundamental differences in *MYC* mRNA expression and protein induction during the initial phase of the differentiation protocol, total RNA and protein were harvested at 2, 6, 12, 24, and 48 hours post-induction and the expression of *MYC* was determined. *MYC* transcript levels increased to a mean level of 2.1 fold over non-induced control cells (Fig. 1B). Interestingly, *MYC* transcript levels remained relatively constant at every time point measured. This pattern in *MYC* expression is consistent with the early mitogenic response observed in other cell types, where *MYC* expression is early and maintained even in density-arrested populations [28],[29]; as would be expected for confluent ASC undergoing differentiation. The increase in transcript levels coincided with a progressive accumulation of endogenous protein levels that peaked to a level of 2-fold over non-induced controls at 24 hours (Fig. 1C). Given the half-life of endogenous *MYC* mRNA is on the order of 10 min [30], with similar expectations for shortened protein half-life [31], the results demonstrate that MYC is expressed in ASC and exhibits moderate induction in response to adipogenic stimulus. Such an increase in endogenous transcript and protein levels following adipogenic stimulation supports a putative role for MYC in promotion of adipogenesis, as well as maintenance of a terminal phenotype.

### *MYC* loss-of-function inhibits adipogenesis

**Figure 2:**
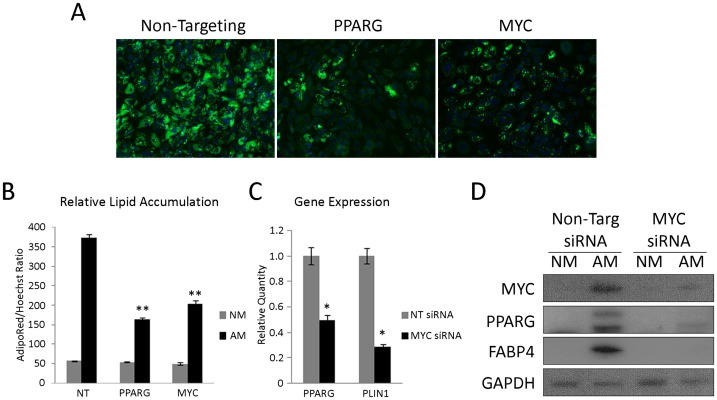
*MYC* loss-of-function inhibits adipogenesis. (A) ASC transfected with non-targeting (NT), *PPARG*, and *MYC* siRNA pools were differentiated and co-stained with AdipoRed (green) and Hoechst (blue). Micrographs at 20X magnification. (B) Relative lipid accumulation was measured for all samples in normal ASC maintenance media (NM) or adipogenic media (AM). 2-way ANOVA with Tukey posttest was used to determine significance relative to NT control; ** p<0.001. Bars represent the mean ± SD. (C) Transcript levels at 7 days of induction for *PPARG* and *PLIN1* was determined by qRT-PCR. Unpaired t-tests were used to determine significance between NT and *MYC* siRNA for each gene; * p<0.05. Bars represent the mean ± SD. (D) Western blot analysis at day 7 of induction was assessed for non-targeting and *MYC* siRNA samples. PPARG and FABP4 were used as terminal biomarkers of mature adipocytes. GAPDH was used as a loading control.

Given the observed increased expression pattern of *MYC* in response to differentiation stimuli, further investigation was initiated to explore the necessity of *MYC* in ASC terminal differentiation. The expression of *MYC* is tightly regulated and controlled on both a transcript and protein level. Loss-of-function analysis was performed by transfecting ASC with pooled siRNA targeting *MYC*. *PPARG* and non-targeting (NT) pooled siRNA duplexes were used as positive and negative controls, respectively. Staining with the neutral lipid stain, AdipoRed, and the nuclear stain Hoechst 33342 enabled quantitative determination of relative lipid accumulation (RLA) in addition to visual inspection. Interestingly, RLA in the *MYC* knockdown sample was qualitatively similar to *PPARG* suppression (Fig. 2A). RLA values determined for *MYC* loss-of-function were also similar to those observed for *PPARG* knockdown (Fig. 2B). To validate that *MYC* knockdown was inhibiting differentiation and not simply affecting lipid accumulation or retention dynamics, transcript levels for *PPARG* and *PLIN1*, a lipid droplet protein expressed in mature adipocytes, were determined following 7 days of differentiation. Relative to non-targeting siRNA controls, *PPARG* and *PLIN1* were reduced 50.0% and 71.4%, respectively (Fig. 2C). Protein levels were also assessed for MYC, PPARG and FABP4, an additional adipogenic marker, at the 7 day termination point. The observed increase in MYC protein expression in the non-targeting siRNA control correlated to elevated levels of both PPARG and FABP4 (Fig. 2D). This expression pattern was largely mitigated in the MYC knockdown samples, where loss of PPARG and FABP4 was evident. The results support the notion that terminal adipose differentiation is inhibited when MYC stabilization is suppressed. In addition, knockdown of MYC protein was confirmed for the pooled siRNA, demonstrating that the duration of siRNA activity could be sustained over the 7 day course of differentiation. Collectively, induced expression of *MYC* during the course of ASC differentiation, coupled with the loss of key adipogenic markers for terminal differentiation under *MYC* suppressive conditions, suggested that *MYC* is essential for human ASC differentiation.

**Figure 3:**
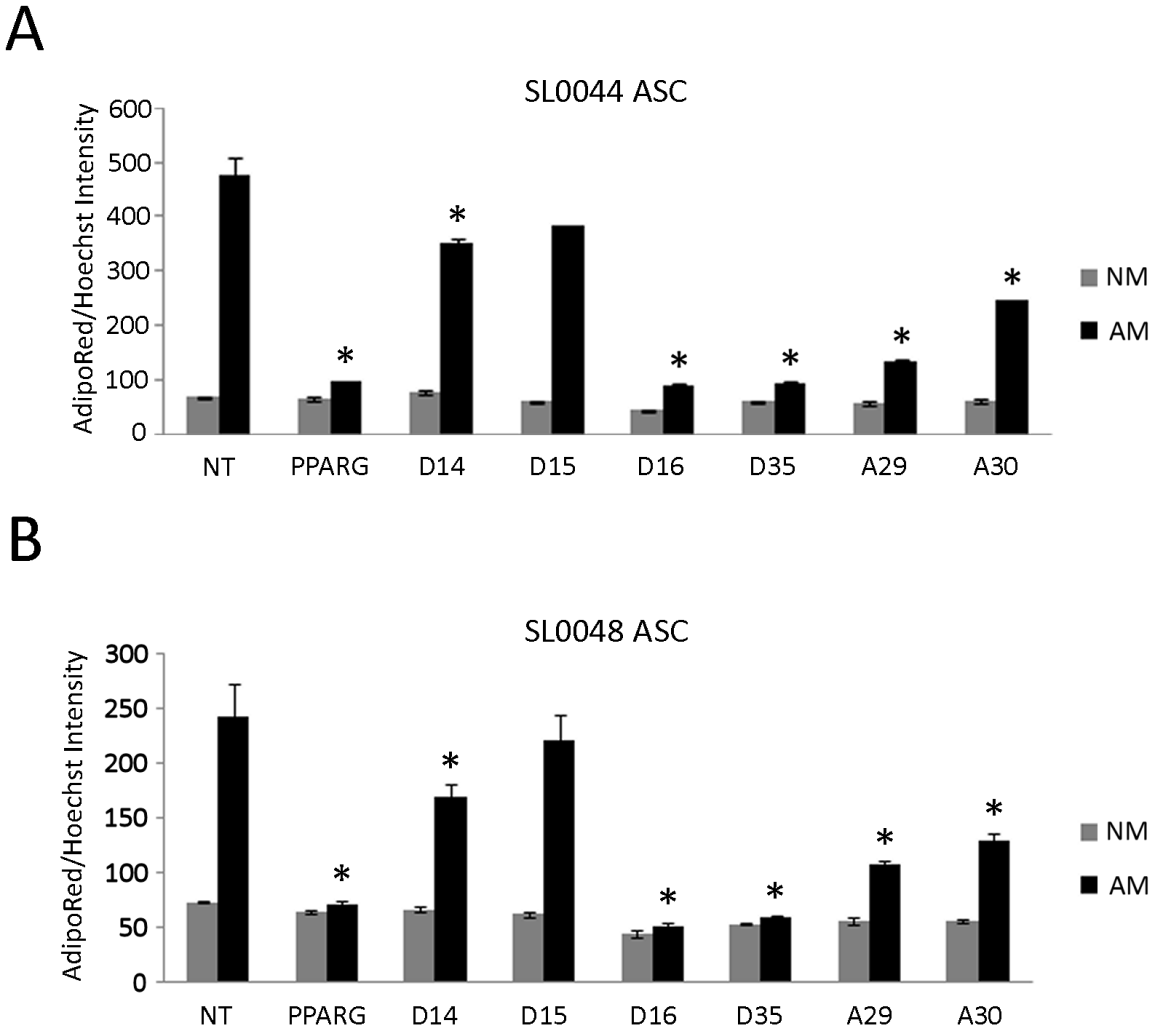
Deconvolution of *MYC* siRNA duplexes and independent ASC donor confirmation. (A) Six independent siRNA duplex sequences were assessed for inhibition of relative lipid accumulation following adipogenesis in primary ASC from a pooled batch of donors (SL0044). (B) Performance of the same six siRNA duplexes were further validated in a second batch of primary ASC from independent donors (SL0048). Two-way ANOVA with Tukey posttest was used to determine significance relative to NT control * p<0.001. NM: Normal ASC maintenance media, AM: Adipogenic media. Bars represent the mean ± SD of 6 experimental replicates.

Since loss-of-function analysis was performed with siRNA pools, deconvolution of the 4 siRNA duplexes enabled assessment of the performance of individual duplexes within the pool (D14, D15, D16, and D35). In addition, 2 untested siRNA duplexes from an alternative manufacturer (A29 and A30) were added, for a total 6, to determine phenotypic replication in primary SL0044 ASC at passage 3. Confirmation of individual *MYC* transcript knockdown at 72 hours by qRT-PCR, prior to adipogenic stimulation, revealed substantial reductions in D14, D16, A29, and A30 siRNA oligos (Fig. S1). Non-targeting and *PPARG* duplexes were used as negative and positive controls, respectively. Following a 72 hour knockdown period, the cells were initiated to differentiate. Of the 6 duplexes tested, D14, D16, D35, A29, and A30 exhibited significant reductions in differentiation (Fig. 3A). D14, D16 and D35 confirm the findings from the original *MYC* siRNA pool, and A29 and A30 provide additional evidence in favor of *MYC* knockdown inhibiting lipid accumulation. Furthermore, the relative cell counts for *MYC* knockdown were indifferent from *PPARG* and non-targeting control siRNA following 72 hour knockdown, just prior to adipogenic stimulation, suggesting that *MYC* knockdown was not indirectly altering adipogenic differentiation by modulating proliferation of the ASC (Fig. S2).

To account for the variability in donor samples and anatomical site of subcutaneous adipose tissue isolation, a second mixed donor pool was screened against the 6 independent *MYC* siRNA duplexes to assess inhibition of RLA. The second primary ASC line (SL0048) was derived from a range of anatomical sites of 8 female Caucasian donors with a mean age of 44 [range: 29–51] and mean BMI of 26.3 [range: 25.1–29.2] (Table 2). The results indicate that the same 5 duplexes (D14, D16, D35, A29, and A30) were able to reproduce the phenotype observed in the previous pooled donor sample (Fig. 3B). The consistent findings of the *MYC* siRNAs across a range of 14 human donors implicates *MYC* in stem cell turnover of human subcutaneous adipose depots in a manner independent of genetic variability.

### *MYC* expression is early and sustained in response to adipogenic stimulus

Two phases encompass differentiation of ASC from multipotency to a defined adipocyte terminal lineage. The first phase is commitment to a preadipocyte whereby a stem cell restricts lineage specification to adipocytes. The second phase is terminal differentiation which involves the requisite transcriptional, epigenetic, and biochemical cascade of events that define the functional characteristics of a terminal adipocyte [32]. The WNT and TGF-β signaling pathways function as key suppressors of the commitment phase. Wnt10b, a secreted glycoprotein that activates the canonical WNT pathway, suppresses adipogenesis in murine cell line models [33],[34]. Reduction of WNT signaling is a key event in the early commitment phase of mesenchymal stem cells to the adipogenic lineage [35],[36]. Similar to *Wnt10b*, *WNT2* also acts through canonical WNT pathway, but has been shown to be decreased even earlier during differentiation of mesenchymal stem cells and was therefore selected as a marker of early commitment [37]. Likewise, *TGFB1* down-regulation is also an early indicator of stem cell commitment and was included in the gene reference set [38]. *CEBPB* and *CEBPD* were included since they have been previously reported as early response factors to adipogenic stimulation, with *CEBPB* coordinating *CEBPA* expression in particular [39]. To define the differentiation phase, *CEBPA*, *PPARG*, *FABP4*, and *PLIN1* were selected as biomarkers. Induction of *CEBPA* is expected to increase prior to downstream activation of *PPARG* with subsequent activation of a positive feedback loop that drives terminal differentiation [40]. Lipid droplet formation and fatty acid metabolism are represented by the lipid droplet protein PLIN1 and fatty acid binding protein FABP4, respectively [41].

**Figure 4:**
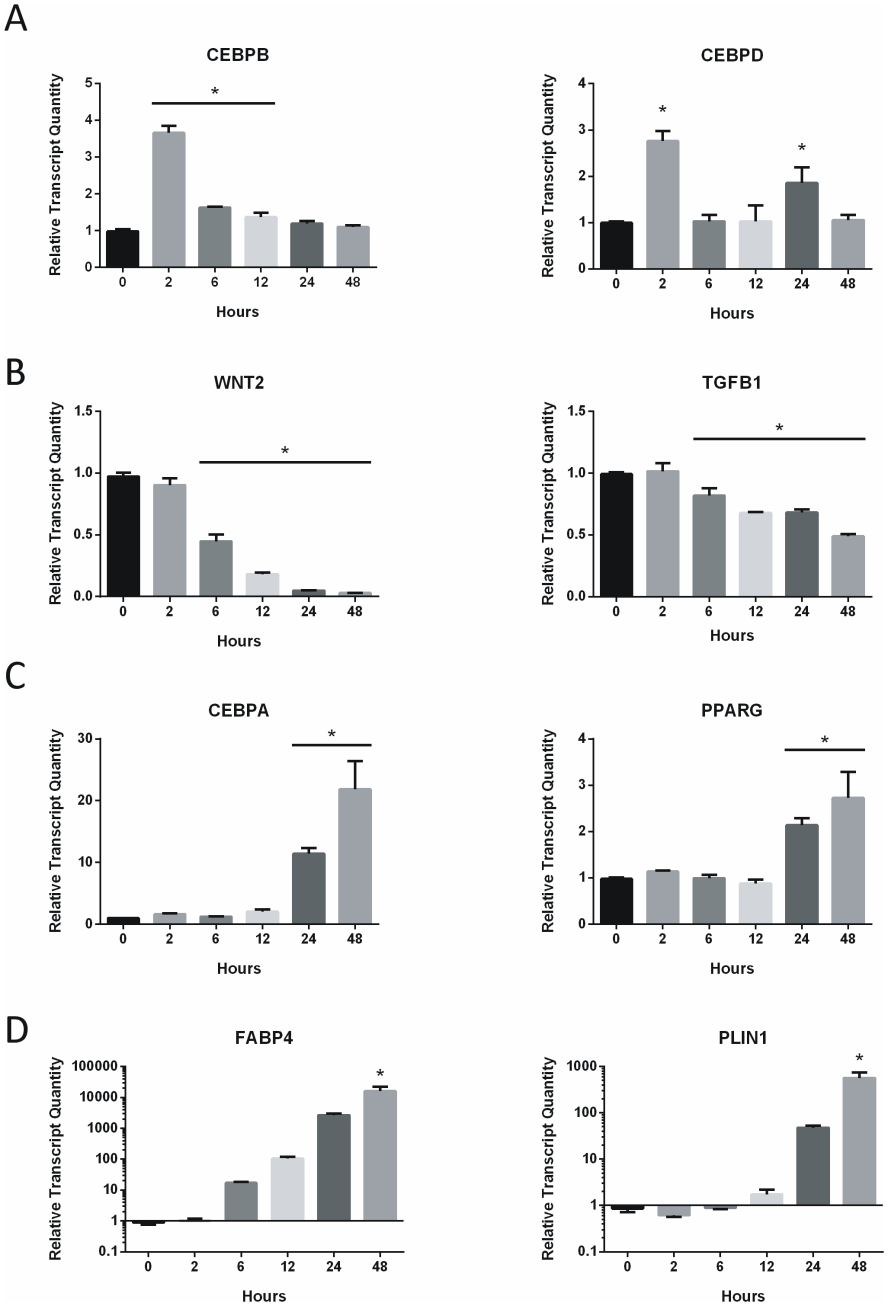
*MYC* expression is early and sustained in response to adipogenic stimulus. 48 hour time course expression of *MYC* and adipogenic markers following induction with adipogenic media was measured using qRT-PCR. After normalization to GAPDH, sample values were normalized to non-targeting reference controls at the 0 hour time point. One-way ANOVA with Dunnett posttest was used to calculate significance relative to 0 hour control * p<0.001. Earliest significant modulation of gene expression post-induction was 2 hours for (A) CEBPB and CEBPD, (B) 6 hours for *WNT2* and *TGFB1*, (C) 24 hours for *CEBPA* and *PPARG*, and (D) 48 hours for *FABP4* and *PLIN1*. Bars represent the mean ± SD of 3 experimental replicates.

Using qRT-PCR measurements, ASC were induced to differentiate and temporal gene expression patterns tracked at 2, 6, 12, 24, and 48 hours. As mentioned previously, increased *MYC* expression was observed at the earliest time point of 2 hours (Fig. 1B), coinciding with peak expression of *CEBPB* and *CEBPD* at this time point (Fig.4A). The expression of *MYC, CEBPB*, and *CEBPD* preceded the down regulation of suppressors *WNT2* and *TGFB1*, which began to decline at 6 hours (Fig. 4B). *CEBPA* and *PPARG* demonstrated increased expression by 24 hours (Fig. 4C), followed by metabolic and structural markers *FABP4* and *PLIN1* at 48 hours (Fig. 4D). Taken together, these data demonstrate that on a population level, the commitment phase may occur within the first 24 hours of adipogenic stimulation with the initial decline in suppressor genes *WNT2* and *TGFB1*, and upregulation of early responders *MYC*, *CEBPB*, and *CEBPD*. This is followed by a secondary terminal differentiation phase with the appearance of *CEBPA* and *PPARG* induction at 24 hours and phenotypic markers *FABP4* and *PLIN1* at 48 hours. Most notably, *MYC* induction is an early event that is sustained throughout the initial course of adipogenesis. The temporal expression pattern supports a role for *MYC* as an early response regulator of adipogenesis.

**Figure 5:**
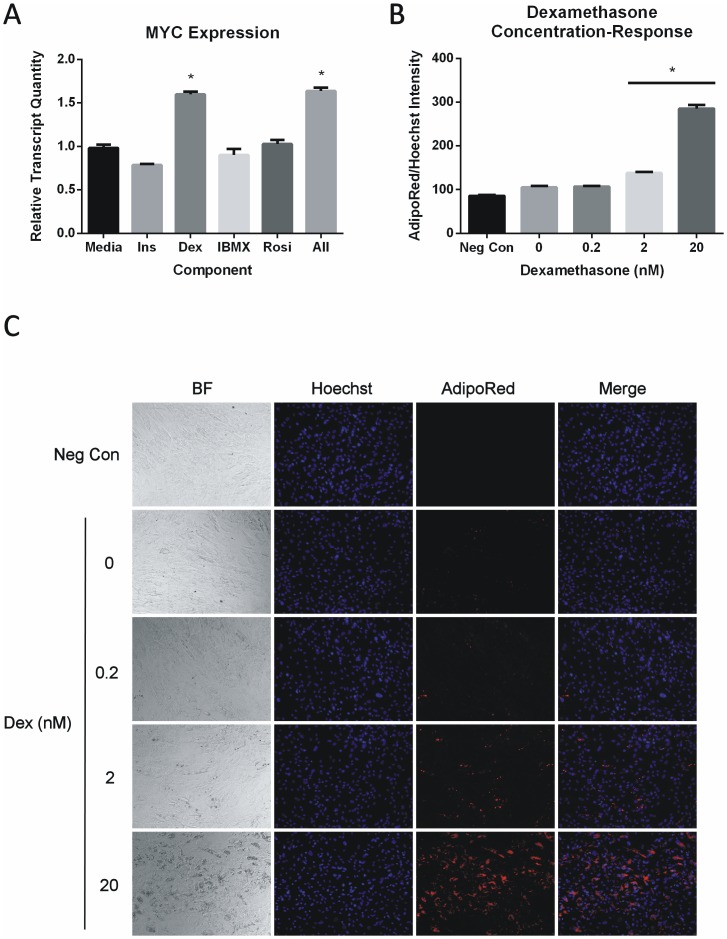
*MYC* is induced by glucocorticoids. (A) ASC were exposed to each individual cocktail component, or the combined mixture, for 24 hours. Transcript expression was determined relative to media treated controls. INS; Insulin, DEX; dexamethasone, IBMX; isobutylmethylxanthine, ROSI; rosiglitazone. (B) ASC exposed to the standard adipogenic cocktail across a dexamethasone dose-curve for 7 days. Negative controls (Neg Con) were left untreated. One-way ANOVA with Dunnett posttest was used to determine significance relative to negative controls * p<0.001. Bars represent the mean ± SD of 3 experimental replicates. (C) Representative images of dexamethasone concentration-response. Micrographs are at 10X magnification.

The adipogenic program is driven by a component cocktail comprised of the synthetic glucorcorticoid dexamethasone, the cAMP agonist isobutylmethylxanthine, the PPARG agonist rosiglitazone, and PI3K/AKT agonist insulin. To determine which component was inducing expression of *MYC*, the component cocktail was uncoupled and exposed to ASC for a period of 24 hours at the same concentrations found in the mixed cocktail. Dexamethasone was the single component inducing *MYC* to comparable levels as the mixed cocktail, with relative quantities of 1.7 and 1.6 over media treated controls, respectively (Fig. 5A). It should be noted that *MYC* transcript levels were likely lower than those previously observed since the cells used for the experiment were at significantly higher passage number. Importantly, the dexamethasone induced transcript levels were comparable to the mixed cocktail control. Glucocorticoid signaling has previously been reported to prime commitment of primary mesenchymal stem cells and C3H10T1/2 cells *in vitro* [42],[43]. To determine if dexamethasone-induced gene expression was essential for adipogenesis, ASC were exposed to the standard adipogenic cocktail containing 0, 0.2, 2, or 20 nM dexamethasone for seven days. RLA was determined and found to demonstrate a positive dose-response relationship with 2 and 20 nM dexamethasone concentrations yielding significant increases in differentiation (Fig. 5B). Moreso, despite the presence of insulin, rosiglitazone, and IBMX, the magnitude of differentiation for samples containing no dexamethasone were comparable to untreated controls (Fig. 5B,C). Based on these observations, early gene expression changes mediated by glucocorticoid signaling may facilitate ASC lineage commitment and differentiation in a *MYC* dependent manner.

### *MYC* siRNA affects key events during human adipogenesis

The knockdown data pointed toward a functional role for *MYC* in driving key events that lead to adipocyte maturation. To gain better insight into the biological processes affected by physiological *MYC* expression, whole genome expression analysis was performed to identify differentially expressed genes between *MYC* knockdown versus non-targeted control ASCs. Cells were again transfected with pooled siRNA for 72 hours prior to initiating differentiation for an additional 72 hours. This time point was selected for analysis since robust expression of key transcriptional drivers *CEBPA* and *PPARG* was previously observed at this point (data not shown). Additionally, given the early expression pattern of *MYC*, this was considered an optimal window to identify functional categories affected by reduced MYC activity.

Of the genes deemed statistically significant in the differential expression analysis, 1149 were up regulated and 966 down regulated relative to siRNA control, for a total of 2115 genes (Table S1). In this context, the up regulated genes are those normally suppressed during the early course of differentiation in a manner dependent on the presence of MYC. Conversely, the down regulated genes are those that are typically induced during differentiation. To achieve a better understanding of the functional relevance of the enriched gene sets, pathway analysis was implemented to define the set of MetaCore Pathway Maps most strongly represented by the gene expression data sets (Table S3). Using a gene fold-change cutoff of 1.5 and a relatively stringent false discovery rate of 0.005, 17 categories were enriched for the down regulated set. Importantly, 2 categories for fat cell differentiation were identified as highly significant and contained classic genes implicated in adipogenesis (e.g. *PPARG*, *CEBPA*, *LPL*, *ADIPOQ*, *LEP*, and *FABP4*), demonstrating that the *MYC* knockdown samples were genuinely inhibiting progression of the adipogenic transcriptional program. The same parameters applied to the up regulated genes enriched for 24 categories. The most notable enriched pathways included processes for development, cell adhesion, and cytoskeletal remodeling.

**Figure 6:**
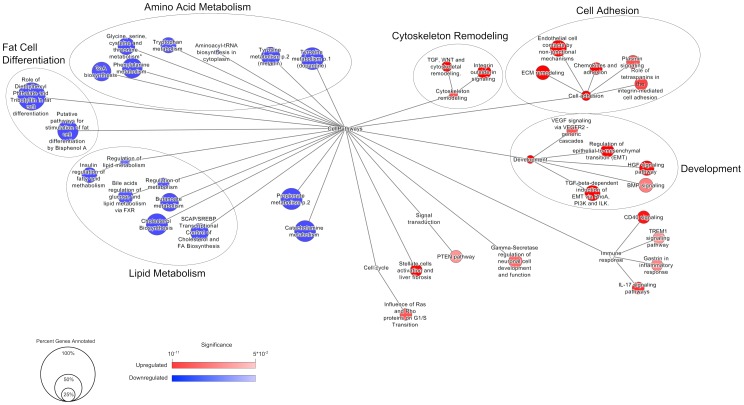
*MYC* loss-of-function alters multiple biological processes during adipogenesis. ASC differentiation was initiated for 72 hours for pooled *MYC* siRNA or non-targeting (NT) control samples and global gene expression patterns determined by mircroarray. A network representation of the MetaCore pathway maps identifies closely related clusters of major biological processes perturbed by *MYC* knockdown. Here, edges reflect the intrinsic structure of the MetaCore ontology, and functional pathways are shown if they are enriched in up regulated (red) or down regulated (blue) genes. The size of the circles corresponds to the percentage of genes identified in each indicated pathway. The color intensity reflects the FDR adjusted p-value assigned to the category.

Using a network representation of functional categories enabled clustering for categories with similar semantic relationships (Fig. 6). Fat cell differentiation, lipid metabolism, and amino acid metabolism were three major clusters enriched in the down regulated gene set. These are highly consistent with the notion that adipogenesis requires induction of genes for transcriptional mediated differentiation, modulation of fatty acid biosynthesis, and increased protein synthesis [4]. More interesting may be the observations of cell adhesion, cytoskeletal remodeling, and developmental processes enriched in the up regulated genes. These are processes that represent inhibitory functions that may be actively suppressed by MYC activity. As an example, with respect to cell adhesion, MYC has been previously reported to suppress N-cadherin (*CDH2*) and integrin expression to drive hematopoietic stem cell differentiation. Here, *CDH2*, and a range of integrins (*ITGA1*, *ITGA4*, *ITGA5*, *ITGA6*, *ITGA9*, *ITGA11*, *ITGB5*, and *ITGBL1*), were all significantly up regulated, suggesting that expression of *MYC* is essential to promote suppression of these genes in response to adipogenic stimulus. Similar functional categorical findings were noted using an independent *MYC* siRNA, s91-29, supporting the observations made with the pooled siRNA sample (Fig. S3; Table S2 and S4). Given these are primary human adipose stem cells, the early events leading to stem cell commitment are less well defined. The findings here highlight a putative functional role for *MYC* in directing key events that lead to altered interaction with the local microenvironment and induction of the transcription dependent gene program integral to ASC adipogenesis.

## Discussion

In this study, the transcription factor *MYC* was identified as a key regulator of human adipogenesis. Unlike the 3T3-L1 and 3T3-F442A mouse clonal cell lines, which are already committed to the adipocyte lineage, the use of low passage, human-derived ASC facilitated investigation into pathways that may regulate commitment of multipotent stem cells to progression through terminal differentiation. Knockdown of *MYC* by multiple siRNA duplexes yielded a functional phenotype of reduced lipid accumulation in two independent donor pools of ASC and was associated with reduced levels of adipogenic markers both at the protein and transcript level. Expression of *MYC* was found to increase over the initial course of differentiation. Increase in *MYC* transcript levels was found to be an early event following adipogenic stimulation, preceding the expression decline of suppressor genes known to be implicated in adipose stem cell commitment [4]. Global gene expression analysis of *MYC* knockdown in ASC revealed perturbations to cell adhesion, cytoskeletal remodeling, developmental signaling, and fat cell differentiation. The data suggest that in the context of ASC maturation, *MYC* may act as an early global regulator of multipotent stem cell commitment and progression to terminal adipocytes.

MYC regulates a diverse array of genes with estimations upwards of 15% of the total transcriptome [44]. Despite repeated attempts to define MYC specific target genes, much of the reported expression profiling appears to define MYC activity as a context-dependent component of the cellular system studied. Several recent studies suggest that MYC functions as a natural enhancer of cell-specific gene expression [45],[46]. In support of the enhancer concept, MYC appears to regulate recruitment of RNA polymerase II to proximal promoters in a manner consistent with regulation of a major portion of the transcriptome [47]. The gene expression data in this study points toward functional roles for MYC in several biological processes during the course of differentiation. For instance, key adipogenic genes (*PPARG*, *CEBPA*, *ADIPOQ*, *LPL*, *LEP*, *PLIN1*, and *FABP4*) were less induced in the absence of normal *MYC* expression, suggesting MYC plays a functional role in initiating and/or maintaining expression of these key genes. In light of these findings, further investigation is necessary to determine if MYC is a general amplifier of the adipogenic program in adipose stem cells. Moreover, the genes bound by MYC in ASC, either directly or indirectly, remain to be determined. Given the evidence for early *MYC* expression, it remains plausible that MYC could accelerate or amplify the progression of these events by recruiting RNA polymerase II to active sites of transcriptional repression or induction in order to drive multipotent stem cells through commitment and terminal differentiation.

Stem cell lineage commitment is a complex process that is influenced by coordinated interactions with the cellular microenvironment including cell-matrix and cell-cell adhesions [48]. Previous investigation into the transcriptome profiles of bovine subcutaneous, omental, and intramuscular adipose tissue depots identified ECM-receptor interactions and focal adhesions as two major developmental processes common among the depot specific cell types [49]. Much like ASC, naïve mesenchymal stem cells direct lineage specification, in part, through responding to the composition of the ECM to impart morphological changes that can influence cellular fate. Indeed, the ability of committed preadipocytes to obtain a rounded morphology is a hallmark feature that distinguishes adipocyte lineage specification from osteogenesis and myogenesis. This suggests that coordinated interactions of ASC with the ECM may be necessary to induce the morphological changes that enable differentiation.

One means of coordinating morphological cues to altered ECM is through modulation of integrin expression; the primary cell surface receptors that mediate the interaction with ECM ligands. ECM-integrin interactions are important for transducing signals through Rho GTPase family members, such as Rac1 and RhoA, to coordinate biological functions like cytoskeletal remodeling and cell motility. One well documented example of a cell-matrix interaction regulating preadipocyte morphology is the role of the fibronectin receptor α5-β1 integrin. Early work in 3T3-L1 cells demonstrated the inhibitory effect of a fibronectin substrate on differentiation and expression of lipogenic markers [50]. Subsequent examination of the α5 integrin subunit showed decreased temporal expression throughout the course of 3T3-L1 differentiation, accompanied by coordinated increase in cell rounding [51]. Through binding to fibronectin, α5 integrin activates Rac1 to promote actin polymerization which inhibits cell rounding [51]. The active suppression of α5 integrin in ASC may be a key event in stem cell differentiation as well. Several previous studies have implicated MYC in suppression of integrins [52],[53], suggesting a direct regulatory connection. Here, several integrins, including α5, and other cell adhesion markers were significantly enriched in the *MYC* knockdown samples, corresponding to an inhibition of differentiation. Suppression of adipose stem cell-matrix interactions by MYC during the course of adipogenesis may be necessary to decrease cytoskeletal tension of the cell in order to promote the subsequent rounded morphology requisite for adipocyte differentiation. Despite the initial observations made here, the potential function of MYC in direct regulation of the genes governing cell-matrix interactions will require additional investigation.

Closely related to cell-matrix interactions is the direct regulation of the cytoskeleton which was shown to be altered in the microarray data in response to *MYC* knockdown. The organization and density of the actin cytoskeleton plays a key role in defining early lineage commitment by altering the structural morphology of mesenchymal stem cells (MSC). Morphometric descriptors of actin cytoskeleton shape, intensity, texture, and spatial distribution, have all been used to accurately profile MSC responsiveness to various substrate matrices that promote osteogenic fate [54]. These findings support the notion that F-actin fiber density functions as an early determinant in driving adipogenic lineage commitment as well. In support of this, morphological changes that occur with mouse 3T3-F442A preadipocyte differentiation are associated with a decrease in synthesis of cytoskeletal associated proteins [55],[56]. Ectopic expression of *MYC* in pig fibroblasts has been reported to depolymerize the F-actin cytoskeleton, leading to reorganization of the cytoskeleton and the subsequent morphological changes reminiscent of an epithelial-to-mesenchymal transition (EMT) [57]. Interestingly, EMT was one pathway enriched in *MYC* knockdown samples as well. This provides additional evidence that MYC may mediate cytoskeletal remodeling in ASC by reducing F-actin polymerization and decreasing expression of cytoskeletal proteins. The requirement for reducing stress fiber formation may be to limit cell-matrix adhesions and cell migration to facilitate “docking” of a committed preadipocyte in the niche where it will terminally differentiate. This is evident in epidermal and hematopoietic cell maturation, where MYC directs exit from the stem cell compartment by altering cell-ECM dynamics to enable terminal differentiation [17],[18],[19],[20],[21]. In addition, F-actin disassembly may provide a more relaxed conformation for differentiating adipocytes to create intracellular space for expanding lipid droplets which will eventually account for the majority of cytosolic space.

The synthetic glucocorticoid hormone, dexamethasone, was identified here as a key component of the adipogenic cocktail and was determined to be responsible for transcriptional induction of *MYC*. Interestingly, dexamethasone treatment of 3T3-L1 preadipocytes was previously associated with regulation of WNT and TGF-β genes and necessary for induction of *CEBPA* and *PPARG* [42]. This suggests a mechanistic link between glucocorticoid signaling and *MYC* expression; a relationship that may contribute toward determination of lineage commitment in adipose stem cells as has been previously suggested for glucocorticoid activation [42],[58]. An increase in central adiposity is a classic long-term consequence associated with prolonged administration of glucocorticoids and is, in part, due to increased hyperplasia within adipose depots [59],[60]. In addition, elevation of the endogenous glucocorticoid cortisol, as in the case of Cushing's syndrome, is also associated with increased rates of obesity. Chronic exposure of adipose stem cells to endogenous, and exogenous, glucocorticoids may increase adipogenesis by progressing stem cells toward committed progenitors, thus sensitizing these cells to adipogenic stimulus. More work is necessary to explore the relationship of glucocorticoids and a MYC dependent gene program in adipogenesis.

In summary, this study establishes the utility of primary adipose stem cells as a model for investigating molecular components of human adipogenesis. Contrary to the inhibitory role of over expressed *MYC* on differentiation in multiple cell systems, the novel finding that physiological *MYC* may actively regulate and promote ASC lineage commitment and progression will contribute to the understanding of human adipose stem cell differentiation. Further investigation is warranted to characterize the significance of *MYC* induction in modulation of ASC cell-matrix interactions, cytoskeletal remodeling, motility, and amplification of the adipogenic program.

## Supporting Information

Figure S1 **MYC siRNA validation.** ASC were transfected with the indicated *MYC* siRNA oligos D14, D15, D16, D35, A29, and A30 and incubated for 72 hours. qRT-PCR analysis was used to evaluate MYC transcript expression in each sample relative to non-targeting (NT) controls. The table indicates the percentage of knockdown relative to NT. Bars represent the mean ± SD of 3 experimental replicates.
(TIF)

Figure S2 **Relative cell counts of MYC siRNA samples.** ASC were transfected with the *PPARG* siRNA, or indicated *MYC* siRNA oligos D14, D15, D16, D35, A29, and A30, or SmartPool (D14-35) and incubated for 72 hours. The cells were fixed with 4% paraformaldehyde and stained with Hoechst 33342. Hoechst intensity was measured for each sample. Bars represent the mean ± SD of 3 experimental replicates. MYC knockdown was not different from PPARG, NT, or untreated controls.
(TIF)

Figure S3 **Independent** ***MYC*** **siRNA alters global gene expression programs during adipogenesis.** ASC differentiation was initiated for 72 hours for s91-29 *MYC* siRNA or non-targeting (NT) control samples and global gene expression patterns determined by mircroarray. A spatial representation of the gene ontology data identifies closely related clusters of major biological processes perturbed by *MYC* knockdown. Up regulated genes indicate enrichment over control, whereas down regulated genes represent depletion. The size of the circles corresponds to the percentage of genes identified in each indicated category. The color intensity correlates to the p-value significance assigned to the category.
(TIF)

Table S1 Gene expression fold-change values for pooled *MYC* siRNA significant genes.
(DOCX)

Table S2 Gene expression fold-change values for s91-29 *MYC* siRNA significant genes.
(DOCX)

Table S3 Gene ontology enrichment for pooled *MYC* siRNA significant genes.
(DOCX)

Table S4 Gene ontology enrichment for s91-29 *MYC* siRNA significant genes.
(DOCX)
