# Supplementary figures and images for: MYC Is an Early Response Regulator of Human Adipogenesis in Adipose Stem Cells
Source: PLoS One. 2014 Dec 1;9(12):e114133. doi: 10.1371/journal.pone.0114133 (PMC4250176; doi:10.1371/journal.pone.0114133)

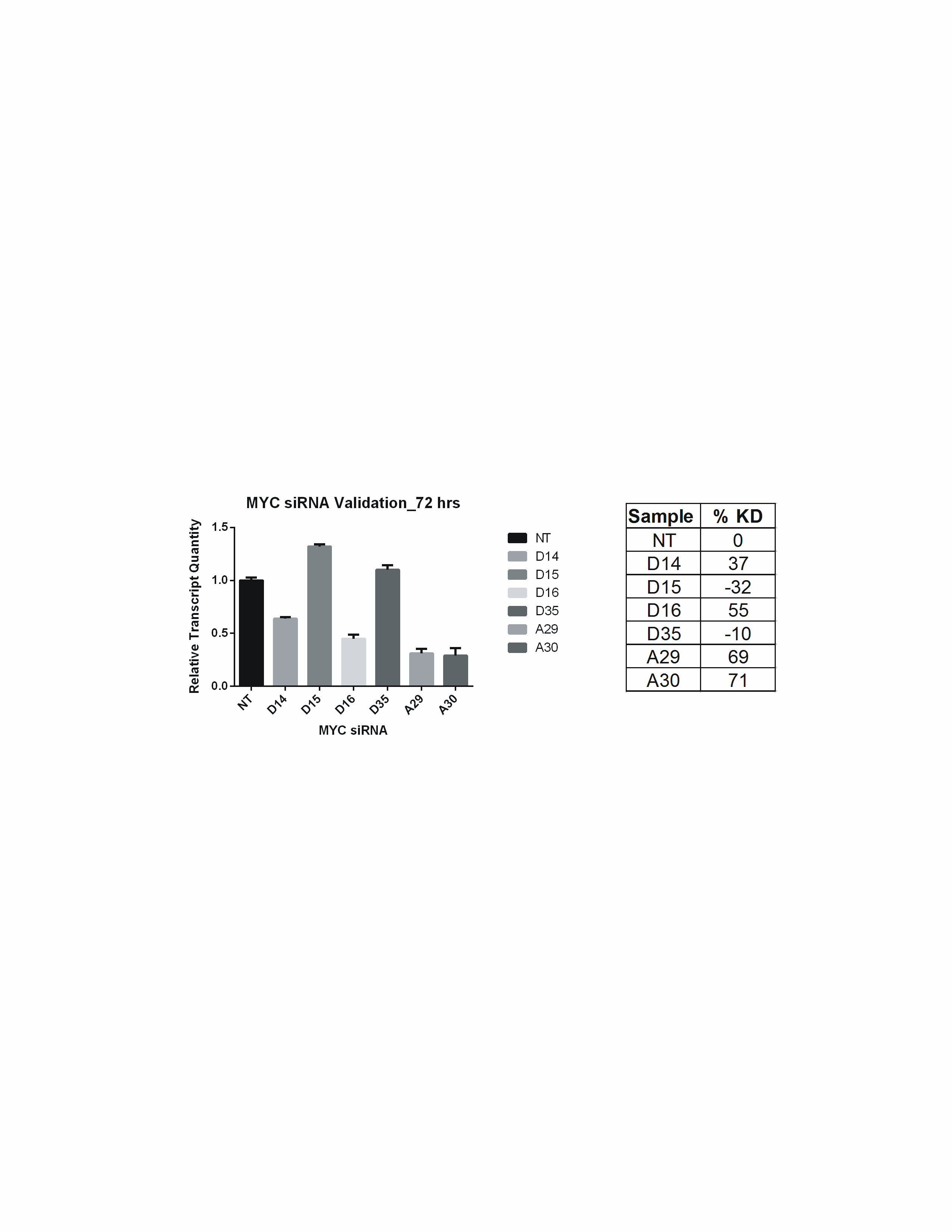

Supplement: Figure S1 — MYC siRNA validation. ASC were transfected with the indicated MYC siRNA oligos D14, D15, D16, D35, A29, and A30 and incubated for 72 hours. qRT-PCR analysis was used to evaluate MYC transcript expression in each sample relative to non-targeting (NT) controls. The table indicates the percentage of knockdown relative to NT. Bars represent the mean ± SD of 3 experimental replicates. (TIF) [file pone.0114133.s001.tif]

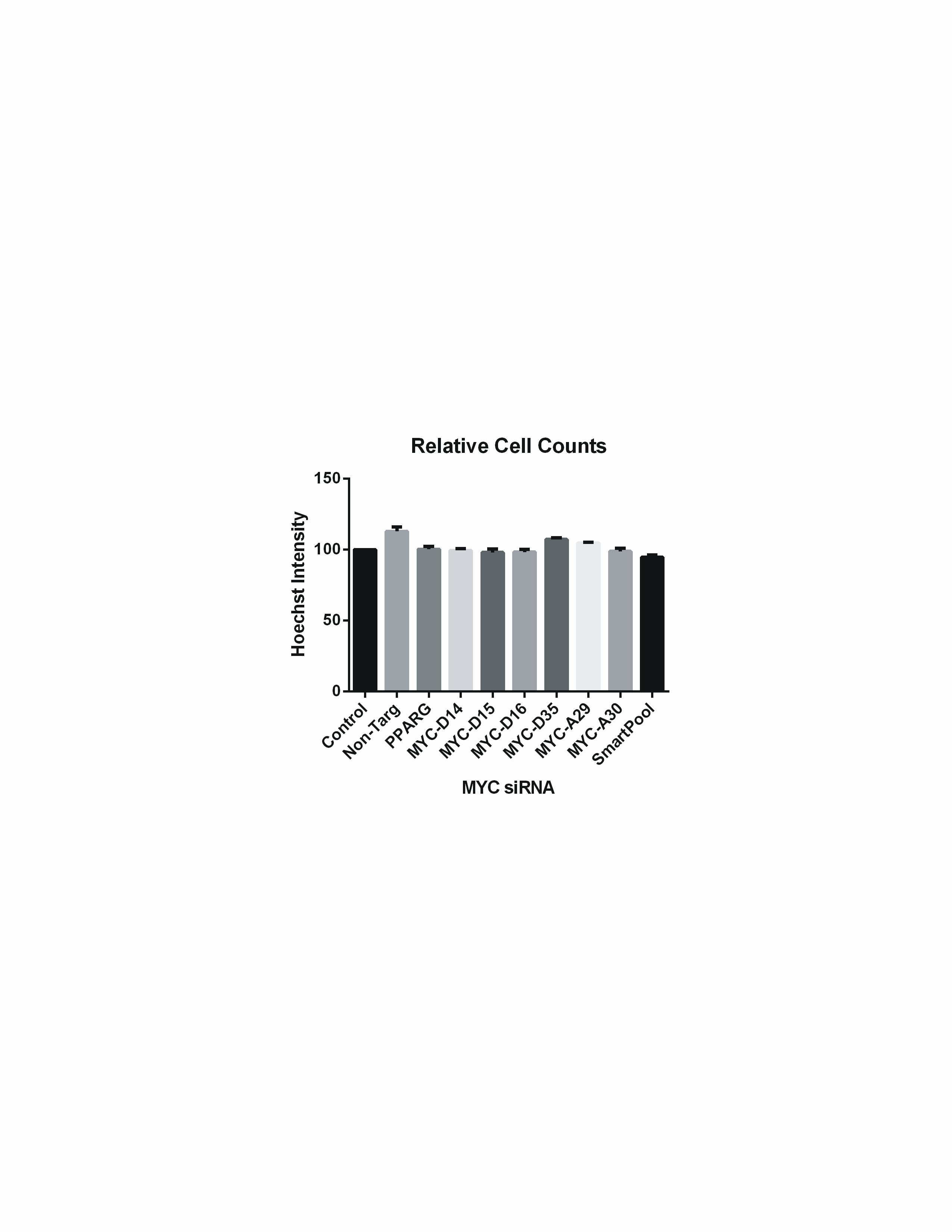

Supplement: Figure S2 — Relative cell counts of MYC siRNA samples. ASC were transfected with the PPARG siRNA, or indicated MYC siRNA oligos D14, D15, D16, D35, A29, and A30, or SmartPool (D14-35) and incubated for 72 hours. The cells were fixed with 4% paraformaldehyde and stained with Hoechst 33342. Hoechst intensity was measured for each sample. Bars represent the mean ± SD of 3 experimental replicates. MYC knockdown was not different from PPARG, NT, or untreated controls. (TIF) [file pone.0114133.s002.tif]

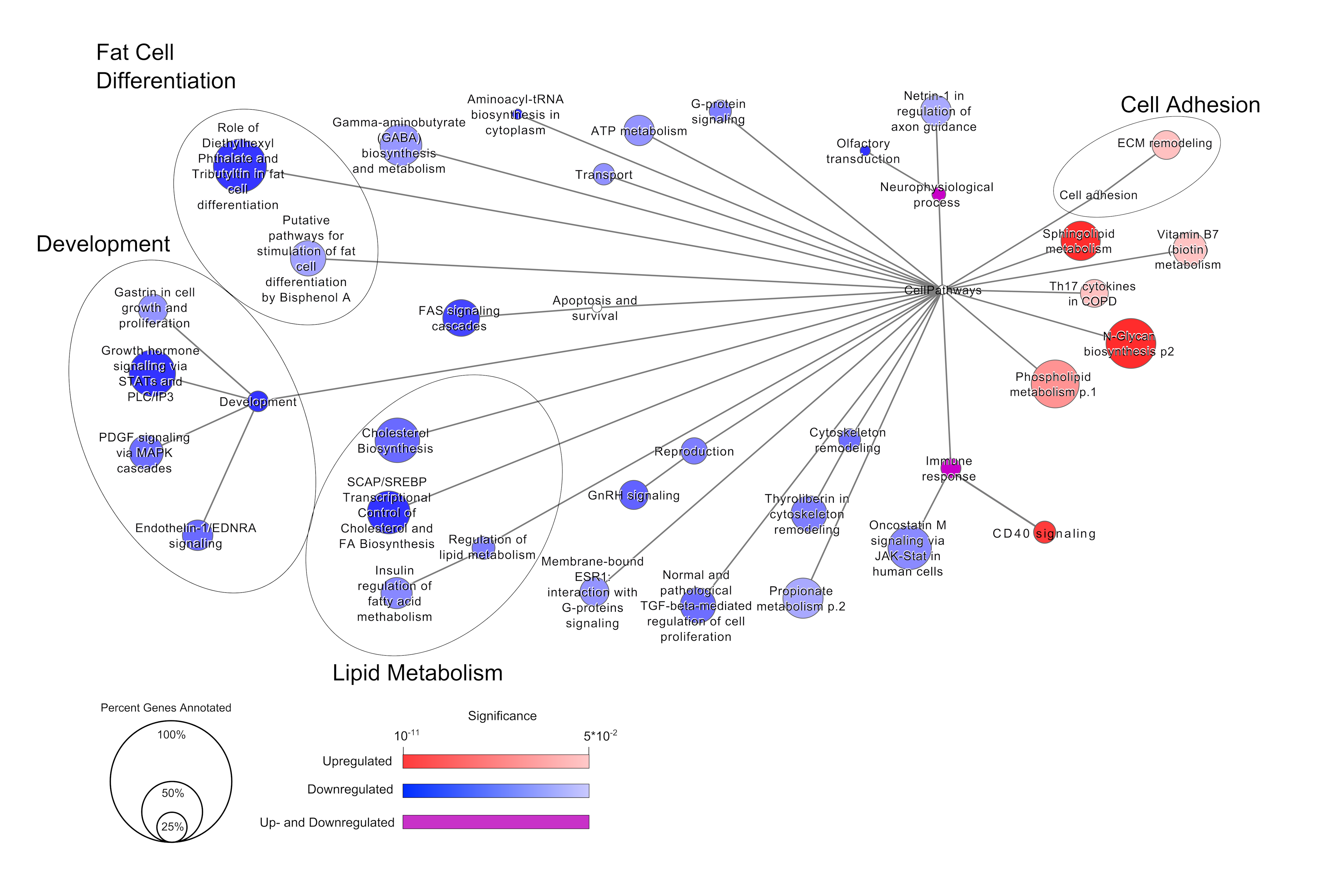

Supplement: Figure S3 — Independent MYC siRNA alters global gene expression programs during adipogenesis. ASC differentiation was initiated for 72 hours for s91-29 MYC siRNA or non-targeting (NT) control samples and global gene expression patterns determined by mircroarray. A spatial representation of the gene ontology data identifies closely related clusters of major biological processes perturbed by MYC knockdown. Up regulated genes indicate enrichment over control, whereas down regulated genes represent depletion. The size of the circles corresponds to the percentage of genes identified in each indicated category. The color intensity correlates to the p-value significance assigned to the category. (TIF) [file pone.0114133.s003.tif]
