# Supplementary material for: MYC Is an Early Response Regulator of Human Adipogenesis in Adipose Stem Cells
Source: PLoS One. 2014 Dec 1;9(12):e114133. doi: 10.1371/journal.pone.0114133 (PMC4250176; doi:10.1371/journal.pone.0114133)
Supplement: Table S1 — Gene expression fold-change values for pooled MYC siRNA significant genes. (DOCX) [file pone.0114133.s004.docx]

**SupplementaryTable 1.** Gene expression fold-change values for pooled *MYC* siRNA significant genes.

| **Pooled down** | **Log2 Fold Change** |  | **Pooled Up** | **Log2 Fold Change** |
| --- | --- | --- | --- | --- |
| PCK1 | -4.766305444 |  | ACTC1 | 3.869669985 |
| ADIPOQ | -4.650777317 |  | OXTR | 2.462913879 |
| LGALS12 | -3.822202624 |  | ANKRD1 | 2.227805205 |
| KLB | -3.601023225 |  | MGARP | 2.219650063 |
| GPI | -3.593651514 |  | SMOC2 | 2.217449691 |
| THRSP | -3.539278596 |  | EFHD1 | 2.174260787 |
| LPL | -3.40584887 |  | DLX2 | 2.169691206 |
| GPD1 | -3.380577388 |  | HTR2B | 2.168147949 |
| PPP1R1A | -3.190233081 |  | RPSA | 2.162929416 |
| PLIN1 | -3.157583552 |  | PAPLN | 2.149694477 |
| FABP3 | -3.068810868 |  | LOC100507165 | 2.142288567 |
| GLDN | -3.065934041 |  | PPP1R14A | 2.079512202 |
| IGFBP5 | -3.045977301 |  | ADRA2A | 2.038573815 |
| IGFBP6 | -2.914833075 |  | PRR15 | 2.007746911 |
| LBP | -2.888185813 |  | MTUS1 | 2.007114137 |
| CYP19A1 | -2.856170834 |  | LDLRAD4 | 1.992081718 |
| PDE3B | -2.679535085 |  | CD200 | 1.986391708 |
| PDLIM2 | -2.606761687 |  | CXCL6 | 1.959195466 |
| AVPR1A | -2.564291984 |  | ITGA9 | 1.91996307 |
| PRKAR2B | -2.550281702 |  | CXCL2 | 1.919291037 |
| RBP4 | -2.521627452 |  | NCEH1 | 1.919149157 |
| HAVCR2 | -2.489952265 |  | DCHS1 | 1.907746341 |
| MRAP | -2.439528087 |  | FAM13C | 1.860576149 |
| PCSK9 | -2.429246162 |  | NME7 | 1.779025903 |
| PALMD | -2.41619024 |  | CXCL1 | 1.772040033 |
| G0S2 | -2.408224148 |  | RERG | 1.762401457 |
| CA2 | -2.393877774 |  | IL8 | 1.716732602 |
| HSD11B1 | -2.328344626 |  | TEK | 1.711070794 |
| CLCA2 | -2.282282775 |  | OMD | 1.691720748 |
| AOC3 | -2.267724489 |  | TNS3 | 1.68878451 |
| KIAA0101 | -2.227338044 |  | NOX4 | 1.688540853 |
| ZNF117 | -2.213473719 |  | IKZF2 | 1.679189192 |
| ITIH5 | -2.210899468 |  | SAMD11 | 1.676281362 |
| OMG | -2.202445575 |  | LIMS2 | 1.660630631 |
| ST6GALNAC5 | -2.158880819 |  | ARHGAP5 | 1.65608315 |
| SCD | -2.121578039 |  | ATP1B1 | 1.640112104 |
| ADH1B | -2.099578161 |  | SIK1 | 1.618412577 |
| FAM134B | -2.093513774 |  | PODXL | 1.615502099 |
| SLC19A3 | -2.072111445 |  | PLAT | 1.60620214 |
| RIMS2 | -2.070688733 |  | PARM1 | 1.591972828 |
| CEBPA | -2.055425456 |  | SLC4A4 | 1.581376641 |
| CLU | -2.048888488 |  | LEPREL1 | 1.581281168 |
| KCND3 | -2.006840072 |  | PCDH10 | 1.577692848 |
| OXCT1 | -1.961066671 |  | TMEM255A | 1.56937746 |
| PLIN4 | -1.935384684 |  | DMD | 1.567131017 |
| IGF1 | -1.933522021 |  | TLR4 | 1.566818526 |
| LNPEP | -1.925074445 |  | FKBP1B | 1.552153175 |
| GAS2L3 | -1.92338648 |  | HMGA2 | 1.549410501 |
| SLC18B1 | -1.921973499 |  | ITGA1 | 1.544094986 |
| C6 | -1.917023689 |  | KCNMB4 | 1.533005098 |
| AGT | -1.902965117 |  | CTGF | 1.519663062 |
| FMO1 | -1.898025378 |  | STEAP4 | 1.514511739 |
| LPIN1 | -1.84222531 |  | COL8A1 | 1.513981974 |
| TSPAN13 | -1.829466966 |  | TPD52L1 | 1.493252053 |
| TLL2 | -1.827487842 |  | BDNF | 1.487920969 |
| DGAT2 | -1.811683338 |  | TNFRSF19 | 1.478275479 |
| MRAS | -1.808090425 |  | PLAU | 1.464109065 |
| CHI3L2 | -1.804955981 |  | EVA1C | 1.462829447 |
| CGREF1 | -1.802728175 |  | LMCD1 | 1.4625142 |
| CA12 | -1.792164107 |  | CADM3 | 1.459685532 |
| SEMA3G | -1.789196347 |  | FMO2 | 1.458742859 |
| ZBED3-AS1 | -1.759701712 |  | LMBR1L | 1.457636282 |
| LEPR | -1.759331519 |  | CORIN | 1.456931931 |
| FABP4 | -1.75787287 |  | IGF2 | 1.449725522 |
| ACVR1C | -1.748895572 |  | WFDC1 | 1.444474215 |
| VIT | -1.733359111 |  | CCL2 | 1.444186821 |
| CLMN | -1.72519251 |  | BMP6 | 1.440026787 |
| RPL22L1 | -1.711975219 |  | A2M | 1.432984923 |
| ZXDB | -1.709989308 |  | LOC728392 | 1.429667994 |
| ADIRF | -1.694368035 |  | KRT18 | 1.426205752 |
| FRMD3 | -1.69401634 |  | B3GALT2 | 1.423176445 |
| STON2 | -1.692085428 |  | EVA1A | 1.416242514 |
| RAB40B | -1.688840053 |  | NEGR1 | 1.414331087 |
| ADCY1 | -1.683612736 |  | TSPAN2 | 1.411685285 |
| LRRN4CL | -1.678577077 |  | FAT3 | 1.411465888 |
| AOC2 | -1.677438841 |  | ARRDC2 | 1.408895692 |
| CA3 | -1.676508501 |  | HAS1 | 1.404665789 |
| PDK4 | -1.671257826 |  | PEAR1 | 1.403562337 |
| HIPK2 | -1.670958835 |  | F2RL2 | 1.394054857 |
| LRRC8B | -1.661280541 |  | SLC16A4 | 1.388780529 |
| TSHR | -1.646333578 |  | PRDM11 | 1.387062206 |
| AOX1 | -1.64262503 |  | SLC46A3 | 1.386931963 |
| RRM2 | -1.642346341 |  | EPHA2 | 1.366952651 |
| TJP2 | -1.637740924 |  | LOC100505573 | 1.365585342 |
| ARSG | -1.635578893 |  | KCNT2 | 1.361525113 |
| GPBAR1 | -1.622440987 |  | PRSS12 | 1.360555357 |
| HRASLS5 | -1.621575715 |  | KCNMA1 | 1.358634279 |
| ADAMTS9 | -1.614659882 |  | MATN3 | 1.351605499 |
| CHST1 | -1.606602415 |  | GDPD1 | 1.350371553 |
| SBSN | -1.60646682 |  | NOG | 1.338208844 |
| OASL | -1.605882247 |  | INHBA | 1.335503829 |
| LAMB3 | -1.604500573 |  | PTPRE | 1.334861599 |
| TPST2 | -1.598357914 |  | PRDM1 | 1.325863365 |
| ACYP2 | -1.592307514 |  | SLC35G1 | 1.319203964 |
| EIF4EBP2 | -1.585284981 |  | RORB | 1.313994895 |
| SHMT1 | -1.582040272 |  | LBH | 1.311535505 |
| ACACB | -1.581553572 |  | SLC9A7 | 1.311102553 |
| FKBP14 | -1.580362929 |  | EGR2 | 1.305381333 |
| YME1L1 | -1.579062413 |  | ENO2 | 1.298182679 |
| C3orf55 | -1.569555749 |  | DDR1 | 1.297030159 |
| UNG | -1.56485894 |  | ZNF204P | 1.291412175 |
| C19orf12 | -1.557236224 |  | CYS1 | 1.285572959 |
| SFXN4 | -1.542658945 |  | PTPRN2 | 1.284038137 |
| CITED1 | -1.533417102 |  | GXYLT1 | 1.278289333 |
| ARHGAP28 | -1.520637253 |  | DNM1 | 1.277866139 |
| PLA2G16 | -1.520058996 |  | SORBS2 | 1.272822659 |
| SUMF2 | -1.519008347 |  | RASL11A | 1.269125843 |
| CTTN | -1.516680622 |  | TNFSF10 | 1.267016218 |
| CHCHD10 | -1.510235237 |  | HOXC13 | 1.253284398 |
| DCLK1 | -1.506131496 |  | PITX2 | 1.252660732 |
| ITGA7 | -1.50373083 |  | ADAMTS6 | 1.251420452 |
| ASPH | -1.502527642 |  | SREK1 | 1.243923879 |
| DMKN | -1.499191222 |  | IMPA2 | 1.23969931 |
| ARHGAP6 | -1.498542193 |  | EMP1 | 1.236007268 |
| BANK1 | -1.485831254 |  | TRIB2 | 1.233324507 |
| RGL1 | -1.482413531 |  | ITGA5 | 1.222504386 |
| HIST2H2BE | -1.474929352 |  | ALOX5AP | 1.214491672 |
| PNPLA3 | -1.470396728 |  | ITGA11 | 1.19536453 |
| MAP2K6 | -1.466324742 |  | RAMP1 | 1.194997332 |
| FNDC4 | -1.465514729 |  | RAPGEF5 | 1.191203879 |
| ABCD2 | -1.443745886 |  | EPHB3 | 1.189790051 |
| ACSL5 | -1.439302784 |  | SERTAD4 | 1.186070689 |
| PGM5-AS1 | -1.432029918 |  | GREM2 | 1.185355985 |
| VEPH1 | -1.430487993 |  | CHAC1 | 1.184025626 |
| CHI3L1 | -1.430362658 |  | FZD6 | 1.1831632 |
| FASN | -1.427071914 |  | SNCAIP | 1.181623156 |
| C2orf88 | -1.41728965 |  | TAGLN | 1.181309083 |
| UCP2 | -1.411566093 |  | IER3 | 1.180574278 |
| OLAH | -1.409457989 |  | TES | 1.168331448 |
| DLAT | -1.40290748 |  | DNER | 1.163715408 |
| CIDEC | -1.400135802 |  | ELN | 1.162593263 |
| CLYBL | -1.387367395 |  | MORN4 | 1.157882694 |
| DUSP6 | -1.387330009 |  | CCDC85A | 1.156133411 |
| MCTP1 | -1.373891382 |  | CBX7 | 1.155989985 |
| MATR3 | -1.373525745 |  | LOC158402 | 1.151691339 |
| MCUR1 | -1.371822211 |  | BEND7 | 1.150397175 |
| TM7SF2 | -1.370773191 |  | ZNF469 | 1.149529155 |
| PTGER3 | -1.358973143 |  | BTN2A2 | 1.148145405 |
| BPNT1 | -1.354170043 |  | TMEM155 | 1.141574695 |
| SDC1 | -1.349485234 |  | TCF21 | 1.140601548 |
| KIAA0226L | -1.338117881 |  | GDNF | 1.140241783 |
| TMEM56 | -1.335729652 |  | SNAI1 | 1.137367532 |
| FGFBP2 | -1.324721434 |  | SYT11 | 1.137002834 |
| TMEM135 | -1.320600263 |  | AUTS2 | 1.136081953 |
| VKORC1L1 | -1.31922294 |  | SLC35D2 | 1.135656269 |
| ZBED3 | -1.316811287 |  | CEP152 | 1.125543988 |
| LOC100505702 | -1.309802055 |  | HOXD1 | 1.123318886 |
| SLC25A18 | -1.309130215 |  | SUSD2 | 1.120460513 |
| LPPR4 | -1.308741312 |  | COL4A4 | 1.109484631 |
| GPER | -1.305706071 |  | MUC1 | 1.108926987 |
| MED8 | -1.305261971 |  | TTLL7 | 1.100873852 |
| HP | -1.305144718 |  | PF4V1 | 1.096858249 |
| RDH5 | -1.301563763 |  | KRT7 | 1.093733477 |
| SMOC1 | -1.298531687 |  | NIPA1 | 1.0936062 |
| LOC339524 | -1.296139236 |  | RAB39B | 1.093118026 |
| ADAMTS15 | -1.29065484 |  | MAGEL2 | 1.092329924 |
| SCARA5 | -1.28804785 |  | CARD10 | 1.092102698 |
| CPB1 | -1.287611547 |  | RAB27B | 1.085702139 |
| ENC1 | -1.287001914 |  | POSTN | 1.085234753 |
| KBTBD11 | -1.273967217 |  | DSTN | 1.08499667 |
| AIFM2 | -1.273893055 |  | ZBTB10 | 1.07923447 |
| ITGA8 | -1.27304973 |  | PTGS2 | 1.077361852 |
| AMPH | -1.271407483 |  | STC2 | 1.0764571 |
| KCTD12 | -1.270812122 |  | SPRY4 | 1.075666804 |
| NPR1 | -1.266970288 |  | KGFLP2 | 1.075147431 |
| LEP | -1.26346286 |  | SYTL2 | 1.074027094 |
| AQP3 | -1.263335694 |  | SSTR1 | 1.073799049 |
| C7orf41 | -1.260451005 |  | NALCN | 1.073647248 |
| ARCN1 | -1.257603159 |  | LIMCH1 | 1.073297022 |
| CDKN2C | -1.255193303 |  | KCNS3 | 1.071641557 |
| SKP2 | -1.255182351 |  | ZFPM2 | 1.070323394 |
| MCF2L | -1.252784076 |  | DCN | 1.067871686 |
| KCNQ3 | -1.249466188 |  | DYX1C1 | 1.065439494 |
| RAB33A | -1.247771485 |  | MPP5 | 1.062791749 |
| RNF125 | -1.247468379 |  | MOB3B | 1.062272708 |
| AKR1C1 | -1.246719756 |  | SYNGR1 | 1.059515038 |
| PRRT3-AS1 | -1.246713268 |  | MCTP2 | 1.059157882 |
| SELENBP1 | -1.244644584 |  | CCNG2 | 1.057961568 |
| CKMT2 | -1.238103826 |  | BEX2 | 1.057861431 |
| WDR12 | -1.236149721 |  | CNN1 | 1.054425974 |
| HIST3H2A | -1.234673181 |  | NEBL | 1.048009722 |
| GUF1 | -1.234585655 |  | ZNF365 | 1.044407427 |
| ENPP2 | -1.232778269 |  | STMN3 | 1.039423033 |
| C20orf27 | -1.230278544 |  | GGT5 | 1.038058891 |
| HTR2A | -1.223279029 |  | SNX24 | 1.034273909 |
| CDK15 | -1.221582965 |  | MECOM | 1.032767925 |
| COL4A6 | -1.220549959 |  | TNFRSF12A | 1.03273612 |
| EMC3 | -1.219631737 |  | BLZF1 | 1.031790316 |
| LPCAT1 | -1.218251139 |  | FAM131B | 1.029032073 |
| INF2 | -1.215011241 |  | TNFRSF10D | 1.027924741 |
| APCDD1 | -1.205649178 |  | F2R | 1.027256951 |
| QPRT | -1.202470056 |  | ITGB5 | 1.025586719 |
| FRMD4A | -1.20044828 |  | MFSD6 | 1.024927632 |
| TMEM30B | -1.200265496 |  | PTPRB | 1.024902543 |
| AFAP1L1 | -1.196540609 |  | STRBP | 1.024303397 |
| AADAC | -1.194359715 |  | TM6SF1 | 1.022202396 |
| ELOVL6 | -1.194212278 |  | THBS3 | 1.021971417 |
| ATPAF2 | -1.191978373 |  | CMKLR1 | 1.019166525 |
| ELOVL3 | -1.191708911 |  | MIR210HG | 1.018267547 |
| CD36 | -1.189592845 |  | EMB | 1.017819931 |
| KCNK3 | -1.188982412 |  | RGCC | 1.016890908 |
| TMEM170B | -1.181736551 |  | NAP1L3 | 1.016093207 |
| PHKA1 | -1.180714657 |  | FAM167A | 1.015832766 |
| PAICS | -1.178537645 |  | ULBP2 | 1.014096003 |
| TMEM38B | -1.175173603 |  | SERPINE1 | 1.012145228 |
| MAFB | -1.173638269 |  | TENM2 | 1.009269391 |
| NOP16 | -1.169668515 |  | NAALAD2 | 1.008928401 |
| CFD | -1.169496113 |  | LOC153682 | 1.007384229 |
| SOS1 | -1.166012087 |  | VAPA | 1.007025762 |
| PPARGC1B | -1.157187823 |  | CYP26B1 | 1.006276742 |
| HS3ST2 | -1.155697335 |  | LCOR | 1.006216978 |
| DHX8 | -1.15568876 |  | TNFRSF1B | 1.005598978 |
| LOC100128822 | -1.15394831 |  | ANKRD33B | 1.003583444 |
| NMB | -1.152361409 |  | PAK1 | 1.000323509 |
| FJX1 | -1.146315819 |  | INPP4B | 1.000154269 |
| CRYAB | -1.142353317 |  | ARSI | 0.995888443 |
| RASD1 | -1.14054415 |  | EGR1 | 0.992700857 |
| DPY19L3 | -1.140139522 |  | IL1RAP | 0.992282849 |
| NLRX1 | -1.133798712 |  | CDH2 | 0.989385509 |
| RCAN1 | -1.13377461 |  | CMTM8 | 0.989260546 |
| PCOLCE2 | -1.130528548 |  | PLK3 | 0.988867841 |
| COASY | -1.129785983 |  | ARSJ | 0.988595004 |
| FERMT1 | -1.122960789 |  | RAB15 | 0.987607065 |
| CDO1 | -1.122142756 |  | EPHA3 | 0.987194174 |
| LOC100507303 | -1.121661731 |  | PIK3CD | 0.986650276 |
| PTPN11 | -1.120826608 |  | DYNLRB2 | 0.982819011 |
| GPR133 | -1.120599847 |  | SLC26A11 | 0.982599797 |
| ADAMTS18 | -1.119347097 |  | KCNK6 | 0.981210953 |
| LOC202781 | -1.117062797 |  | VEGFC | 0.980855991 |
| DUSP4 | -1.11664122 |  | RPS6KA6 | 0.980086315 |
| MGC45800 | -1.1148863 |  | SEPT8 | 0.979229409 |
| THEMIS2 | -1.11229877 |  | FGF7 | 0.977896375 |
| ACSS2 | -1.10777811 |  | KIAA1598 | 0.977755179 |
| GHR | -1.101370385 |  | SLC12A8 | 0.977746282 |
| MARC1 | -1.100750291 |  | SMAD7 | 0.977324121 |
| MDFIC | -1.100067661 |  | EDIL3 | 0.975718641 |
| ALKBH2 | -1.0933398 |  | SYNE1 | 0.974515329 |
| TRIP13 | -1.093182752 |  | AEBP1 | 0.973940933 |
| GLUD2 | -1.091971676 |  | ACTA2 | 0.971447753 |
| SMIM15 | -1.091294355 |  | DSP | 0.970630932 |
| SMIM10 | -1.090015011 |  | CRLF1 | 0.970326169 |
| NR1H3 | -1.08864748 |  | LMOD1 | 0.965585358 |
| NPR3 | -1.088128592 |  | AXL | 0.965164237 |
| SYNPO | -1.088064501 |  | GPRC5A | 0.964708924 |
| PLAC9 | -1.085119455 |  | PDE5A | 0.964246745 |
| POLR1E | -1.08495937 |  | SMAD9 | 0.964240237 |
| LOC100289098 | -1.084706343 |  | MPRIP | 0.963109912 |
| SLCO4C1 | -1.082935339 |  | IL15 | 0.961697614 |
| MBNL3 | -1.082717438 |  | PLCB4 | 0.9612112 |
| TAF4B | -1.078847693 |  | CTDSPL2 | 0.960683111 |
| NOVA1 | -1.078651496 |  | CSRNP1 | 0.960574549 |
| INHBB | -1.077539611 |  | TANC2 | 0.959411128 |
| DOCK11 | -1.077222096 |  | SCUBE3 | 0.957873799 |
| LOC100506325 | -1.073349304 |  | LOC100289187 | 0.955132795 |
| GPAM | -1.072381604 |  | NPNT | 0.954115403 |
| APOC1 | -1.071486136 |  | C10orf12 | 0.953638023 |
| DCXR | -1.070902949 |  | TET1 | 0.953290203 |
| SREBF1 | -1.069946902 |  | FXR1 | 0.951326944 |
| XPNPEP2 | -1.068078896 |  | PPAPDC1A | 0.950066044 |
| FAM83H | -1.066626577 |  | PGM2L1 | 0.949889466 |
| FAM213A | -1.063536776 |  | MRVI1 | 0.948759917 |
| HAS2 | -1.060689435 |  | THBS1 | 0.948264254 |
| GPD1L | -1.06052678 |  | GATS | 0.946442324 |
| DEXI | -1.059523883 |  | HOXD-AS1 | 0.946172122 |
| ACSF2 | -1.058881427 |  | ZBTB46 | 0.944329964 |
| ABHD5 | -1.054824878 |  | ZDBF2 | 0.941982806 |
| TIMM8B | -1.052295948 |  | GFPT1 | 0.94108916 |
| MCAM | -1.050625726 |  | ERP44 | 0.941065769 |
| MKX | -1.050142204 |  | UBE2D3 | 0.94085923 |
| TOP2A | -1.047644856 |  | PFKFB4 | 0.939804376 |
| INSIG1 | -1.046516358 |  | SYNJ2 | 0.938676147 |
| TACC2 | -1.045475729 |  | ARMC9 | 0.938335181 |
| CSNK2A1 | -1.043266486 |  | CARHSP1 | 0.938145087 |
| IL21R | -1.041412768 |  | HMCN1 | 0.937996892 |
| EBP | -1.041406913 |  | LTBP2 | 0.935194046 |
| C11orf71 | -1.039603482 |  | HIP1 | 0.934697041 |
| SDC2 | -1.039522098 |  | IFITM10 | 0.934605529 |
| SLC38A5 | -1.0392038 |  | TMEM2 | 0.934569263 |
| C21orf119 | -1.039005171 |  | ATXN1 | 0.932701917 |
| MSR1 | -1.034610504 |  | HCP5 | 0.930755415 |
| OSR1 | -1.033239413 |  | FLNB | 0.930711935 |
| BBOX1 | -1.032995788 |  | PCDH7 | 0.930258053 |
| PDE8B | -1.031781993 |  | DNAJB4 | 0.929521986 |
| HLA-DMA | -1.030800072 |  | DNAJC10 | 0.92942096 |
| PANK3 | -1.029675133 |  | NR3C2 | 0.928034653 |
| RBBP6 | -1.029086031 |  | JAM2 | 0.927482978 |
| PFKFB3 | -1.026698914 |  | FGF2 | 0.92543815 |
| NDUFA8 | -1.025060092 |  | ATL1 | 0.925070661 |
| APPL1 | -1.024734378 |  | MAP7D2 | 0.92443646 |
| CILP | -1.01880689 |  | RABGAP1L | 0.923760624 |
| LINC00936 | -1.018486411 |  | KIF26B | 0.923244564 |
| PRELP | -1.017546399 |  | NCOA3 | 0.922798354 |
| KLF15 | -1.016235388 |  | NRK | 0.91945726 |
| PNPLA4 | -1.015299229 |  | ADAM19 | 0.918916545 |
| GRB14 | -1.014117983 |  | SHB | 0.917817275 |
| USP18 | -1.013971466 |  | SKIL | 0.917151258 |
| PPL | -1.01390761 |  | C12orf75 | 0.916745006 |
| PPP2R1B | -1.013521789 |  | CBLB | 0.915193554 |
| C12orf39 | -1.009950182 |  | SLC31A2 | 0.914216807 |
| FAH | -1.00981272 |  | ENPP5 | 0.913886345 |
| TPX2 | -1.009655358 |  | FAT4 | 0.913458312 |
| ASS1 | -1.008542717 |  | FAM73A | 0.912313629 |
| ST3GAL4-AS1 | -1.006816226 |  | CITED2 | 0.911616386 |
| PIK3C2B | -1.006666395 |  | C20orf112 | 0.910969496 |
| PPM1L | -1.00572196 |  | SSBP2 | 0.910537064 |
| LINC00341 | -1.005511027 |  | LMO7 | 0.910253102 |
| ZNF436 | -1.00346043 |  | LOC100996381 | 0.910038853 |
| ADAT2 | -1.003199698 |  | QKI | 0.908601521 |
| GLIS3 | -1.001359944 |  | ACTR3 | 0.90773409 |
| MARCH2 | -1.001087717 |  | SRSF12 | 0.907365464 |
| COL21A1 | -0.992505274 |  | ARMC8 | 0.906772456 |
| FAM216A | -0.991468791 |  | TMEM67 | 0.904586407 |
| SPOP | -0.985906328 |  | KLF5 | 0.904081993 |
| PDCD11 | -0.985007489 |  | CDKN2D | 0.903275226 |
| MAOA | -0.984655491 |  | RASA2 | 0.902988404 |
| FEN1 | -0.982287625 |  | CLDN12 | 0.901895862 |
| CSAD | -0.98198857 |  | SPRED1 | 0.900560164 |
| ALDH6A1 | -0.981587726 |  | KCND2 | 0.900325505 |
| ADAMTS3 | -0.980052313 |  | HRH1 | 0.899667294 |
| PTPN22 | -0.977890924 |  | RAB36 | 0.898925926 |
| CTH | -0.976543161 |  | TRMT10A | 0.898457579 |
| FAM195A | -0.975807158 |  | SAMD4A | 0.897777194 |
| LOC100287387 | -0.975237974 |  | CCDC136 | 0.897606518 |
| ABCF2 | -0.975208116 |  | PRRT2 | 0.897110808 |
| CHAC2 | -0.971093456 |  | CCDC68 | 0.895641801 |
| PARVA | -0.966996407 |  | EID3 | 0.895599335 |
| LIMS1 | -0.9656504 |  | TDG | 0.89483569 |
| PRKAG2-AS1 | -0.962328323 |  | USP25 | 0.894392175 |
| ATP5F1 | -0.960842885 |  | LOC283508 | 0.892702312 |
| RDH10 | -0.957950234 |  | ENAH | 0.890929358 |
| STAR | -0.957397637 |  | NEDD4 | 0.890724322 |
| TMEM97 | -0.957155093 |  | TECPR2 | 0.890599644 |
| DHRS9 | -0.956611585 |  | PPP1R36 | 0.890555953 |
| MLXIPL | -0.95248926 |  | KCNJ6 | 0.890339066 |
| FAM60A | -0.952476825 |  | PALM | 0.88956908 |
| RAB42 | -0.952043304 |  | ARNT2 | 0.886181079 |
| PFKM | -0.950448029 |  | FOXQ1 | 0.885908147 |
| ACSM3 | -0.949317904 |  | GABBR1 | 0.885808632 |
| COMT | -0.949285852 |  | MAFF | 0.885580991 |
| FAM168B | -0.946536034 |  | RHEBL1 | 0.884589699 |
| ATP2A2 | -0.945768583 |  | LIMK2 | 0.883627011 |
| GUCY1A3 | -0.945505167 |  | CNTNAP1 | 0.883308704 |
| PANK1 | -0.939705177 |  | GPR68 | 0.882669382 |
| UAP1 | -0.938806562 |  | PSMD11 | 0.882658994 |
| CHL1 | -0.937868974 |  | API5 | 0.882045918 |
| SNX6 | -0.936941585 |  | SNED1 | 0.881892641 |
| ST6GALNAC4 | -0.936913682 |  | REEP4 | 0.87965412 |
| METTL22 | -0.936779403 |  | OSR2 | 0.879563974 |
| EPB41L4B | -0.93263271 |  | AP1S2 | 0.873592848 |
| PSMD5 | -0.932278689 |  | KIAA1147 | 0.87258336 |
| ARHGAP42 | -0.930008364 |  | B3GALNT1 | 0.872300674 |
| PLA2G4A | -0.929961321 |  | HAPLN1 | 0.872266411 |
| MITF | -0.928937977 |  | DLEU2 | 0.871359051 |
| SLC2A10 | -0.927607073 |  | TMEM17 | 0.870770232 |
| FLJ37453 | -0.926805402 |  | IFT88 | 0.870198568 |
| PREB | -0.926696496 |  | MAP1B | 0.868332553 |
| DDX20 | -0.926498175 |  | CYR61 | 0.868286377 |
| LOC100379224 | -0.926372162 |  | SERTAD4-AS1 | 0.868049579 |
| BRE | -0.925081487 |  | TPM3 | 0.864462722 |
| ADAM12 | -0.924689408 |  | MYADM | 0.86423606 |
| LSS | -0.921532076 |  | FBXL2 | 0.863217165 |
| LOC100507312 | -0.921515341 |  | WDR54 | 0.862768189 |
| XPR1 | -0.917189881 |  | TDRD7 | 0.861621811 |
| SLC25A45 | -0.914118322 |  | TIAM1 | 0.860681133 |
| RHOU | -0.91317888 |  | ZC3H6 | 0.860626695 |
| BZW2 | -0.911798614 |  | ZDHHC13 | 0.859820014 |
| EYA3 | -0.91115956 |  | IGDCC4 | 0.859614578 |
| ZNF106 | -0.911021524 |  | FPR1 | 0.858520271 |
| STOM | -0.908527692 |  | MIR31HG | 0.857810825 |
| IWS1 | -0.907302933 |  | DKK1 | 0.855410292 |
| NLE1 | -0.906554818 |  | RANBP2 | 0.852877787 |
| LPCAT3 | -0.905746384 |  | ADAMTS1 | 0.852571027 |
| PPP1R3E | -0.904940376 |  | CCL28 | 0.852292977 |
| WWC1 | -0.901501905 |  | KCNE4 | 0.851600905 |
| STEAP1 | -0.900851579 |  | FBLN5 | 0.851549734 |
| LOC100996578 | -0.900650163 |  | SECISBP2 | 0.849837644 |
| ERV3-1 | -0.899603496 |  | TTC30B | 0.848104035 |
| SOX12 | -0.899436816 |  | C15orf48 | 0.84786634 |
| LOC100507376 | -0.899023003 |  | STXBP6 | 0.846846781 |
| HSPB8 | -0.898435832 |  | SLC16A3 | 0.845792397 |
| DCAF4 | -0.898241036 |  | EML6 | 0.845622492 |
| PAPPA2 | -0.897057951 |  | HOXD8 | 0.844864642 |
| FZD4 | -0.896509263 |  | METTL4 | 0.842145461 |
| DDIT4L | -0.892162323 |  | HLA-F | 0.842034657 |
| TOB2 | -0.891298025 |  | ITSN2 | 0.840696599 |
| MRGPRF | -0.888482614 |  | RNF141 | 0.839940206 |
| HPGD | -0.887790264 |  | FAM169A | 0.839147477 |
| FAM65C | -0.887692502 |  | RORA | 0.838593587 |
| NANOS1 | -0.886523054 |  | SPP1 | 0.836495179 |
| CSTA | -0.885910491 |  | APBA1 | 0.835151096 |
| EXOSC5 | -0.884039499 |  | RPL13 | 0.835002705 |
| PPIF | -0.883996988 |  | PLAG1 | 0.834314766 |
| CALCRL | -0.882799334 |  | YOD1 | 0.834298914 |
| EPB41 | -0.882381823 |  | PAPOLG | 0.834059578 |
| PDSS1 | -0.881762944 |  | C7orf60 | 0.833713961 |
| TRHDE | -0.880765952 |  | KIAA0355 | 0.833407828 |
| PTGFR | -0.879995831 |  | PIFO | 0.833344973 |
| PC | -0.878553796 |  | CRISPLD1 | 0.83207966 |
| FAM107B | -0.878146958 |  | PEG10 | 0.831552586 |
| CCNB2 | -0.877941971 |  | LRCH2 | 0.828320471 |
| ZC3H18 | -0.877411693 |  | NAP1L1 | 0.827094243 |
| PDP2 | -0.876893409 |  | ULK2 | 0.826042363 |
| DHCR24 | -0.876082685 |  | ANKH | 0.823422124 |
| MX2 | -0.875079129 |  | TPD52 | 0.821858251 |
| RAC3 | -0.874980088 |  | GALC | 0.821396499 |
| SLC39A8 | -0.874213061 |  | LRRC15 | 0.820313436 |
| TRAM2-AS1 | -0.872616796 |  | MET | 0.819397182 |
| MRRF | -0.871312492 |  | CEP135 | 0.818421903 |
| APLN | -0.871178179 |  | SNN | 0.817346794 |
| GM2A | -0.870829911 |  | HLA-B | 0.815940156 |
| LOC728819 | -0.87026135 |  | LOC654342 | 0.815215142 |
| HILPDA | -0.869595381 |  | CD274 | 0.814661276 |
| NEFH | -0.869476491 |  | AGTRAP | 0.813815188 |
| PTER | -0.86830994 |  | C18orf54 | 0.813608553 |
| FLVCR2 | -0.868166889 |  | PCM1 | 0.813028277 |
| DPP7 | -0.867670176 |  | COL12A1 | 0.812163286 |
| CCDC69 | -0.866514722 |  | ITM2C | 0.811889824 |
| HSDL2 | -0.86530282 |  | TRPM7 | 0.81171373 |
| LOC100507642 | -0.864445733 |  | FNDC3B | 0.811111524 |
| PLA2G2A | -0.864317178 |  | KCNJ15 | 0.810568901 |
| CTSC | -0.864212819 |  | STK11IP | 0.809897436 |
| TOP1MT | -0.863272843 |  | APH1B | 0.808276603 |
| LOC389906 | -0.862309622 |  | TMEM8A | 0.808177539 |
| TXLNG | -0.861402446 |  | PIP4K2C | 0.808088929 |
| TSPAN14 | -0.860259145 |  | ANTXR1 | 0.807958962 |
| MAP1LC3B2 | -0.858814229 |  | CNR1 | 0.807449602 |
| F11R | -0.857795666 |  | AQP1 | 0.806201687 |
| LINC00472 | -0.857750998 |  | CYB561D1 | 0.805765433 |
| BMPER | -0.857456825 |  | TMEM87B | 0.805429681 |
| NOP2 | -0.856834302 |  | VRK3 | 0.805285201 |
| MTERFD3 | -0.855334326 |  | EPS8L2 | 0.8047077 |
| KDSR | -0.854720544 |  | TRPC4 | 0.804064796 |
| FOXP2 | -0.853865062 |  | MALL | 0.803806037 |
| UFSP1 | -0.853207555 |  | ZFHX4-AS1 | 0.803225669 |
| PIGL | -0.85305075 |  | FN1 | 0.803124906 |
| HSPD1 | -0.852832709 |  | CPT1A | 0.803073706 |
| MRO | -0.852074036 |  | RAB14 | 0.802906944 |
| EIF2AK4 | -0.851500406 |  | OSBPL5 | 0.801644977 |
| CYB5A | -0.851207769 |  | PURG | 0.801244826 |
| DNAJC14 | -0.85087353 |  | HHAT | 0.800389048 |
| FDPS | -0.850335431 |  | RGL2 | 0.799874538 |
| LOC645984 | -0.850240198 |  | KLHL28 | 0.79939181 |
| DYNLT1 | -0.850050266 |  | PARP8 | 0.798218298 |
| PDPN | -0.849491594 |  | SIPA1L2 | 0.798009518 |
| HADH | -0.849183628 |  | IL6 | 0.796918052 |
| LYNX1 | -0.848515374 |  | FAM63B | 0.79583425 |
| GYG2 | -0.848409258 |  | SUCO | 0.794392688 |
| TLE1 | -0.84712235 |  | PLEKHG1 | 0.794293341 |
| XPO7 | -0.846511767 |  | PHLPP2 | 0.793476022 |
| BCKDHB | -0.846457563 |  | DHRSX | 0.793232656 |
| LOC401052 | -0.846160118 |  | ANO3 | 0.793201778 |
| GPR125 | -0.845477007 |  | NFASC | 0.792678151 |
| C1orf85 | -0.845448661 |  | VLDLR | 0.792360358 |
| CENPV | -0.844390069 |  | MTMR3 | 0.791792793 |
| FKBP7 | -0.841514982 |  | COL3A1 | 0.791614615 |
| CMTM7 | -0.841289459 |  | TNRC6C | 0.791491369 |
| DNAJC4 | -0.840596993 |  | FLJ43663 | 0.791357988 |
| SLC25A1 | -0.839822488 |  | TRIP11 | 0.789686063 |
| MTSS1 | -0.839428394 |  | TLE4 | 0.7880392 |
| TFAM | -0.839175668 |  | MAP4K3 | 0.787427187 |
| KIF7 | -0.837956203 |  | PSD3 | 0.787329865 |
| BDH1 | -0.83787453 |  | PVRL2 | 0.787272978 |
| ST6GALNAC3 | -0.837527137 |  | ELOVL4 | 0.785799127 |
| BOP1 | -0.836846665 |  | ITGBL1 | 0.78565546 |
| EBF1 | -0.83626952 |  | TET2 | 0.785511231 |
| LPP-AS2 | -0.835720842 |  | MARCH4 | 0.785473361 |
| PTGIS | -0.835133161 |  | LAMA1 | 0.783432795 |
| SNX10 | -0.834693062 |  | KIAA1429 | 0.782676178 |
| KIAA0930 | -0.834116757 |  | ZBTB20 | 0.782212975 |
| ENY2 | -0.833842395 |  | LINC00312 | 0.782160971 |
| CEP192 | -0.832371859 |  | PRRG4 | 0.781700664 |
| VANGL1 | -0.831200483 |  | NFKBIE | 0.781462395 |
| TRIM25 | -0.830899341 |  | GPR85 | 0.781440078 |
| LOC100506054 | -0.830647203 |  | SLC6A6 | 0.780783365 |
| C7orf55 | -0.830484034 |  | WIPF2 | 0.779380368 |
| FAM149A | -0.829833584 |  | STX3 | 0.779011163 |
| MOSPD2 | -0.829799224 |  | OPN3 | 0.77853818 |
| SERPINB2 | -0.827113045 |  | DNAJC18 | 0.778391574 |
| HIVEP3 | -0.827086971 |  | SGPL1 | 0.778274137 |
| CLDN23 | -0.826920491 |  | IFITM1 | 0.777306804 |
| IDH1-AS1 | -0.826637125 |  | HSPB7 | 0.777165458 |
| C9orf41 | -0.825674333 |  | WDR78 | 0.776430015 |
| AKAP1 | -0.825419455 |  | DKK3 | 0.776345607 |
| CLOCK | -0.825381127 |  | ERBB2 | 0.775901912 |
| HNRNPA1 | -0.825107867 |  | PML | 0.775767118 |
| IL15RA | -0.822562899 |  | PDGFRL | 0.775295623 |
| ALDH1B1 | -0.821265478 |  | FMN2 | 0.774926753 |
| DHTKD1 | -0.820916299 |  | STON1 | 0.774847717 |
| PCYT2 | -0.820523251 |  | ANKRD50 | 0.774752878 |
| ERAP1 | -0.820361888 |  | PELO | 0.774498683 |
| PTTG1 | -0.820157371 |  | HEXIM1 | 0.773499609 |
| ZDHHC2 | -0.820041022 |  | SENP5 | 0.773312753 |
| EVI2A | -0.818277694 |  | ANKRD52 | 0.773175113 |
| HEATR1 | -0.817672914 |  | SENP2 | 0.772552974 |
| SLC6A15 | -0.815114132 |  | WAC | 0.770880711 |
| FITM2 | -0.814847753 |  | ICAM1 | 0.770558915 |
| APOL6 | -0.81429919 |  | FBLIM1 | 0.770059725 |
| CAT | -0.813961307 |  | FAM161B | 0.769790183 |
| LOC729970 | -0.813665003 |  | TMEM59L | 0.76876486 |
| PLIN5 | -0.81333874 |  | PNISR | 0.768075812 |
| SLC25A23 | -0.812620125 |  | SALL2 | 0.767299329 |
| SLC25A44 | -0.812312762 |  | GNB5 | 0.766498191 |
| USP46-AS1 | -0.80910444 |  | TM7SF3 | 0.765924274 |
| DHCR7 | -0.808093664 |  | COL8A2 | 0.765728522 |
| TMEM35 | -0.807875299 |  | TMEM87A | 0.765076637 |
| ACAA2 | -0.807809235 |  | ZNF709 | 0.765007355 |
| ENO1 | -0.807433956 |  | NF1 | 0.763853837 |
| AIM1 | -0.807083533 |  | MIR503HG | 0.763669379 |
| B3GALT6 | -0.806589859 |  | SPRYD7 | 0.763003633 |
| MESP1 | -0.804427139 |  | DISP2 | 0.762455085 |
| RABEPK | -0.803514114 |  | BTN2A1 | 0.761280521 |
| ADCK3 | -0.801880484 |  | MCPH1 | 0.761216971 |
| MYO1D | -0.800678005 |  | MBOAT2 | 0.760214645 |
| C12orf45 | -0.800004988 |  | TRERF1 | 0.759435366 |
| LOC100288675 | -0.797227844 |  | FZD2 | 0.759242377 |
| ACAT2 | -0.794541426 |  | EDN1 | 0.75922668 |
| SLC41A2 | -0.792457999 |  | NRXN3 | 0.759184961 |
| P2RX4 | -0.792210853 |  | UBE2QL1 | 0.758902296 |
| ENSA | -0.791468025 |  | USP51 | 0.758067176 |
| SLC2A6 | -0.789522608 |  | PHF10 | 0.756853072 |
| LOC153546 | -0.788586944 |  | ALCAM | 0.756174666 |
| EPT1 | -0.788554732 |  | COL14A1 | 0.755788574 |
| SCARB1 | -0.78847673 |  | CYFIP2 | 0.754763112 |
| GEMIN4 | -0.78822239 |  | DIO2 | 0.753852598 |
| NXT1 | -0.786587108 |  | UACA | 0.753703078 |
| SORT1 | -0.785620422 |  | SLC20A2 | 0.753422464 |
| CHP1 | -0.78517768 |  | IRAK1BP1 | 0.752525849 |
| LOC100049716 | -0.784946757 |  | KIAA1211 | 0.752348801 |
| SFRP4 | -0.783796096 |  | AKT3 | 0.752315302 |
| ZNF124 | -0.781890845 |  | ATP10A | 0.752305092 |
| PPARGC1A | -0.779812022 |  | MICAL2 | 0.751875941 |
| DIRC3 | -0.779769179 |  | MTMR9 | 0.751736939 |
| SNHG17 | -0.779539757 |  | TTBK2 | 0.751713998 |
| ILF3-AS1 | -0.779460668 |  | PTPRJ | 0.751498254 |
| TOMM34 | -0.779258864 |  | MMP16 | 0.751480182 |
| GRIA3 | -0.778688595 |  | SVIP | 0.751478035 |
| NOLC1 | -0.777825684 |  | SH3BP4 | 0.751135467 |
| ZMAT3 | -0.776799166 |  | HMGB2 | 0.751047529 |
| BIRC5 | -0.774765117 |  | LAMA5 | 0.750991815 |
| BYSL | -0.774115322 |  | C2orf68 | 0.749132895 |
| RNF41 | -0.774066065 |  | MICB | 0.748765935 |
| IMPDH2 | -0.773984673 |  | PTPN12 | 0.748485721 |
| NEFL | -0.773786851 |  | ID1 | 0.748207535 |
| SAMM50 | -0.771902169 |  | ITPRIP | 0.748081112 |
| BPHL | -0.771312573 |  | FHL2 | 0.747882116 |
| ANKRD46 | -0.770754462 |  | FGF18 | 0.747151146 |
| IVNS1ABP | -0.770065684 |  | LEPROTL1 | 0.746691702 |
| WDR3 | -0.768845282 |  | ATPAF1 | 0.746435867 |
| RAP2C | -0.768573393 |  | CHIC2 | 0.746345147 |
| PPARG | -0.767706783 |  | MAGI1 | 0.746315327 |
| SSH2 | -0.76721701 |  | MOV10 | 0.745884101 |
| FGF14-AS2 | -0.766685063 |  | KIF3A | 0.744733647 |
| TM4SF1 | -0.766653343 |  | LOC401320 | 0.744040696 |
| TK2 | -0.765720713 |  | MGEA5 | 0.743860523 |
| TRAP1 | -0.7652564 |  | NLRC5 | 0.743408391 |
| ALG9 | -0.763676706 |  | HGF | 0.743264627 |
| ZNF485 | -0.763549441 |  | ADAM22 | 0.741764012 |
| IL1RN | -0.763067206 |  | GAS7 | 0.741500599 |
| CTPS1 | -0.763057351 |  | CHN2 | 0.741409869 |
| CDADC1 | -0.762885418 |  | ELL2 | 0.741348214 |
| FKBP11 | -0.762820755 |  | RAB3B | 0.741096009 |
| FBXO9 | -0.762548562 |  | LOXL1-AS1 | 0.740902856 |
| PCCB | -0.761645312 |  | KIAA0226 | 0.740487577 |
| CCDC169 | -0.761638757 |  | CFH | 0.740297128 |
| TRIM2 | -0.759929311 |  | PTK2 | 0.73972331 |
| GUCY1B3 | -0.759694579 |  | DENND5B | 0.739677758 |
| VMA21 | -0.758368558 |  | LAMP3 | 0.737970683 |
| RFXAP | -0.757892885 |  | TNFAIP1 | 0.737580202 |
| HLA-F-AS1 | -0.757370232 |  | GBP3 | 0.736769044 |
| PRDX6 | -0.757211716 |  | LOC284454 | 0.736752473 |
| SHPRH | -0.75618929 |  | ZNF439 | 0.736732472 |
| IMP4 | -0.75581333 |  | MIR100HG | 0.736177837 |
| ANK2 | -0.755325787 |  | EPG5 | 0.735933026 |
| SUV39H2 | -0.75434621 |  | MAPK7 | 0.734967407 |
| MEF2C | -0.754004456 |  | FGD4 | 0.734710087 |
| MRPL2 | -0.7534735 |  | EOGT | 0.734428391 |
| TBL1X | -0.75346225 |  | GALNT7 | 0.733781258 |
| MANEA | -0.75042702 |  | C20orf194 | 0.732533165 |
| MMD | -0.750393746 |  | GPR107 | 0.731693658 |
| CDH12 | -0.750267933 |  | VPS33A | 0.731502689 |
| AQPEP | -0.750070222 |  | LOC283357 | 0.730742987 |
| NETO2 | -0.749335234 |  | FST | 0.730396892 |
| FMOD | -0.749153515 |  | NEK3 | 0.730204231 |
| COG5 | -0.747903367 |  | SGK223 | 0.730151272 |
| DBI | -0.74637859 |  | NUFIP2 | 0.729919703 |
| ADAM32 | -0.746262974 |  | PDLIM7 | 0.728893409 |
| DPM3 | -0.745021262 |  | TECPR1 | 0.728888573 |
| CD320 | -0.745018101 |  | PHLDA1 | 0.728744435 |
| FZD7 | -0.743540068 |  | PBX3 | 0.728627259 |
| CPED1 | -0.742613612 |  | ETNK1 | 0.727662119 |
| SLC25A20 | -0.741953799 |  | NDNF | 0.727229709 |
| HIST1H1C | -0.741808664 |  | NRIP3 | 0.726866946 |
| XPNPEP3 | -0.741504057 |  | TAP1 | 0.725229584 |
| FBLN1 | -0.741132796 |  | SLC25A37 | 0.725169189 |
| REPS2 | -0.741023644 |  | AGPAT9 | 0.725075243 |
| CRABP2 | -0.740919898 |  | FRA10AC1 | 0.724507767 |
| ITPK1 | -0.74084937 |  | AP3M1 | 0.724454045 |
| TUG1 | -0.74052652 |  | CLEC2B | 0.724259084 |
| SHMT2 | -0.740196938 |  | NFATC1 | 0.724028281 |
| NDUFA7 | -0.739847992 |  | ITGA6 | 0.723628435 |
| MAOB | -0.739093389 |  | RHOBTB2 | 0.723544343 |
| EYA1 | -0.736709005 |  | NAGPA | 0.723033811 |
| CYTL1 | -0.735715627 |  | BRD7 | 0.721971515 |
| ERI1 | -0.735031801 |  | GRAMD1B | 0.721383292 |
| ADHFE1 | -0.734844667 |  | SFXN1 | 0.720899195 |
| ACTR3B | -0.734153784 |  | HOXC4 | 0.720443371 |
| ZCCHC14 | -0.732653337 |  | DOCK10 | 0.719881571 |
| MAST4 | -0.732599208 |  | CTIF | 0.719103529 |
| ERN1 | -0.732130178 |  | LOC100507463 | 0.719090242 |
| GALNT12 | -0.732078012 |  | SEC11C | 0.71862486 |
| NSDHL | -0.731965417 |  | ALDH1A1 | 0.718571321 |
| PINLYP | -0.731379569 |  | BCAS4 | 0.718113779 |
| C3orf37 | -0.73135074 |  | RASAL2 | 0.718058485 |
| MARC2 | -0.730850364 |  | PLEKHA8 | 0.717857816 |
| FOXN3 | -0.729550197 |  | RGMB | 0.717473712 |
| MGC12916 | -0.728555885 |  | STXBP5 | 0.717152153 |
| C14orf159 | -0.728513542 |  | CXCL5 | 0.717095333 |
| SETMAR | -0.728475807 |  | DUSP18 | 0.717047537 |
| G3BP1 | -0.727493512 |  | ZFYVE26 | 0.716800816 |
| TRMT5 | -0.727477086 |  | PFKP | 0.716349729 |
| METTL10 | -0.727475839 |  | ZFP36 | 0.715926993 |
| CBS | -0.726866752 |  | FAS | 0.715887294 |
| ACOX2 | -0.726011772 |  | LOC374443 | 0.715519331 |
| LARS | -0.725321563 |  | PRL | 0.71546213 |
| GPATCH4 | -0.725103398 |  | AMPD3 | 0.714656711 |
| CLIC6 | -0.724864892 |  | IL18R1 | 0.714543936 |
| VTI1A | -0.72445813 |  | MCOLN3 | 0.713672091 |
| CAMK1 | -0.723651867 |  | AHR | 0.713240826 |
| MSTO1 | -0.723589337 |  | FUT4 | 0.712480588 |
| FNBP1 | -0.72311028 |  | SERPINI1 | 0.712336415 |
| SLC47A1 | -0.722625965 |  | HEG1 | 0.712132706 |
| C4orf46 | -0.722338081 |  | ALG10B | 0.711809992 |
| FLVCR1-AS1 | -0.721686255 |  | NXPE3 | 0.711048657 |
| TMEM9B | -0.721160406 |  | B3GNT1 | 0.710947999 |
| SERPINA3 | -0.720998781 |  | PPFIBP1 | 0.710770064 |
| CDCA7L | -0.720940036 |  | KANK4 | 0.710748397 |
| C19orf60 | -0.720506726 |  | MLLT10 | 0.710164429 |
| PEMT | -0.720413809 |  | FZD1 | 0.70986811 |
| NFIB | -0.720329128 |  | FAM217B | 0.70914477 |
| LINC00476 | -0.719470627 |  | SLC38A9 | 0.708302062 |
| ARHGEF9 | -0.719053107 |  | P4HTM | 0.708243037 |
| HSPB6 | -0.717374052 |  | WNT5B | 0.708226538 |
| SEC16B | -0.716930822 |  | TUBD1 | 0.708201002 |
| TPT1-AS1 | -0.716758135 |  | PCSK1 | 0.708069771 |
| KIF21A | -0.716180683 |  | CREBRF | 0.707832641 |
| SNX5 | -0.715314708 |  | SAMD9 | 0.707709606 |
| ACN9 | -0.714449805 |  | PCTP | 0.707615093 |
| TMEM133 | -0.714317636 |  | C8orf37 | 0.707429852 |
| CDC6 | -0.713912908 |  | SPRY2 | 0.707403299 |
| DEAF1 | -0.713272615 |  | OSTM1 | 0.706987596 |
| UBE3D | -0.713237801 |  | NR2F2 | 0.706967395 |
| LOC100506469 | -0.712810182 |  | BAZ2B | 0.70691735 |
| GABPB1-AS1 | -0.712204563 |  | DUSP1 | 0.706598347 |
| PRMT3 | -0.710707311 |  | CELF1 | 0.70616464 |
| FBXO28 | -0.710145112 |  | FRYL | 0.706082035 |
| NIPSNAP1 | -0.709997975 |  | SMYD2 | 0.705503595 |
| PTPRD | -0.708985653 |  | AHRR | 0.705147071 |
| ABHD14A | -0.707280138 |  | FAT1 | 0.704627798 |
| ZNF883 | -0.706479895 |  | KHNYN | 0.704016268 |
| MMAB | -0.706210873 |  | APCDD1L | 0.703698679 |
| FGF13 | -0.70593811 |  | PPP1R3C | 0.703698138 |
| NME1 | -0.705664093 |  | PHF17 | 0.703177856 |
| GPC4 | -0.704330284 |  | BACH2 | 0.703065562 |
| MED10 | -0.703635329 |  | MAFG | 0.702539844 |
| F8 | -0.702954442 |  | PASK | 0.702272286 |
| SESN3 | -0.700758875 |  | SPTLC2 | 0.702008348 |
| ASPA | -0.700213243 |  | MED28 | 0.701791209 |
| LTV1 | -0.699837343 |  | PPP1R13L | 0.701131242 |
| OLFML2B | -0.699542489 |  | SLC44A2 | 0.700834808 |
| INTS7 | -0.699506362 |  | TFPI | 0.700545666 |
| SIX1 | -0.699099508 |  | HLA-J | 0.700299248 |
| C11orf73 | -0.698876348 |  | LACTB | 0.700225468 |
| NPM3 | -0.698541677 |  | MIER3 | 0.698997748 |
| APRT | -0.698229106 |  | KHDC1 | 0.698459896 |
| ARNT | -0.698125699 |  | CSRP1 | 0.697992691 |
| TIGD1 | -0.697946089 |  | CAMK2N2 | 0.697627419 |
| LMNB1 | -0.697313549 |  | LRRC6 | 0.696724985 |
| SCFD2 | -0.697232795 |  | ARID3A | 0.695625873 |
| SQLE | -0.696910179 |  | ADC | 0.695292529 |
| IMPAD1 | -0.696474807 |  | AGO4 | 0.69461222 |
| SORD | -0.695305203 |  | WDR34 | 0.694416854 |
| NHLRC3 | -0.695274917 |  | TAS2R14 | 0.693980443 |
| HSPB2 | -0.694590095 |  | RCAN3 | 0.69379505 |
| RSL1D1 | -0.694130427 |  | SFR1 | 0.693659916 |
| SLC35F5 | -0.693966186 |  | COL11A1 | 0.692788003 |
| LOC100652768 | -0.692713191 |  | MAP3K2 | 0.692455024 |
| MGP | -0.692398677 |  | ZBTB5 | 0.691733182 |
| TRHDE-AS1 | -0.692207405 |  | KCNS2 | 0.691730083 |
| SCRN2 | -0.691462102 |  | DSG2 | 0.691655948 |
| TOR1B | -0.691455718 |  | HTATIP2 | 0.691537539 |
| SLC25A51 | -0.690492678 |  | SHC4 | 0.691415237 |
| IP6K2 | -0.689945042 |  | SGTB | 0.69099655 |
| FARSB | -0.689746045 |  | HOXB7 | 0.690688327 |
| CTSH | -0.688907016 |  | CABLES2 | 0.689825596 |
| LOC283075 | -0.688727417 |  | SKIDA1 | 0.689153232 |
| HK2 | -0.687537914 |  | ASAP2 | 0.688865974 |
| ST3GAL1 | -0.687116872 |  | DNM3OS | 0.688416753 |
| RAB20 | -0.687109882 |  | CRIP2 | 0.688358548 |
| PIGW | -0.686979451 |  | SCAMP1 | 0.688210062 |
| ISOC2 | -0.686813682 |  | KMT2C | 0.68783386 |
| SIAE | -0.686410306 |  | FEM1B | 0.6878134 |
| NTNG1 | -0.685958422 |  | PHF15 | 0.687320946 |
| OPLAH | -0.684603885 |  | ABCA8 | 0.687320235 |
| VPS26A | -0.684373235 |  | SH3RF1 | 0.686694034 |
| ROR2 | -0.684263997 |  | LRP6 | 0.686111575 |
| AZGP1 | -0.682276268 |  | SEC22C | 0.685986259 |
| ZFAS1 | -0.681791129 |  | STAMBPL1 | 0.685871366 |
| PALM2 | -0.680455837 |  | IL6R | 0.685786411 |
| MLH3 | -0.679023043 |  | TMEM50A | 0.685750766 |
| LOC100289361 | -0.678833117 |  | RB1 | 0.685606606 |
| TOMM20 | -0.677877849 |  | RIT1 | 0.685569064 |
| RBM15B | -0.677679413 |  | ERG | 0.684140748 |
| PNPO | -0.677532545 |  | LCA5 | 0.682809923 |
| HS2ST1 | -0.677208485 |  | B3GALTL | 0.681188869 |
| FGFR1OP | -0.67664578 |  | ANKRD28 | 0.68099171 |
| ATP1A2 | -0.676456694 |  | TSPAN5 | 0.680582949 |
| GPHN | -0.676353644 |  | PLCE1 | 0.680424899 |
| CYC1 | -0.67620475 |  | PSTPIP2 | 0.680219069 |
| PTGFRN | -0.675992858 |  | USP12 | 0.679917553 |
| CXorf24 | -0.675112737 |  | TUSC2 | 0.679304197 |
| EVI2B | -0.675010362 |  | TBC1D22B | 0.678941615 |
| ENGASE | -0.674842754 |  | AGAP1 | 0.678409365 |
| OAS1 | -0.674816529 |  | TAPT1 | 0.677713679 |
| TMEM53 | -0.674191634 |  | DACT1 | 0.677668338 |
| AGMAT | -0.673975979 |  | MAPKAP1 | 0.677535298 |
| TOMM22 | -0.673469155 |  | KBTBD2 | 0.677245952 |
| SLC7A11 | -0.673147464 |  | KIF1B | 0.677208048 |
| COL10A1 | -0.671734963 |  | BBX | 0.677131397 |
| PHYH | -0.671446499 |  | USP9X | 0.676799885 |
| CENPF | -0.671264362 |  | PAMR1 | 0.676333691 |
| IPO4 | -0.671057769 |  | RASSF8-AS1 | 0.676287381 |
| CNTNAP4 | -0.670937108 |  | FCHO2 | 0.676279146 |
| SMAP1 | -0.67052343 |  | GIPC2 | 0.675681245 |
| LOC100509635 | -0.669831845 |  | EFHC1 | 0.675613419 |
| APBB1IP | -0.669740881 |  | FGF5 | 0.67544329 |
| TIGD7 | -0.669066472 |  | AREL1 | 0.675360557 |
| TWIST2 | -0.668896128 |  | EHD3 | 0.674930423 |
| NCBP2-AS2 | -0.66862508 |  | AGPAT4 | 0.674910878 |
| RBFA | -0.668033526 |  | IL11 | 0.674759311 |
| ARRB1 | -0.667956634 |  | GLI2 | 0.674530402 |
| HMBS | -0.667566723 |  | ZC3H12C | 0.674295531 |
| ANP32A | -0.66672068 |  | NEDD9 | 0.674016 |
| MRPS16 | -0.664952293 |  | CDKL1 | 0.673693143 |
| DANCR | -0.664671556 |  | PDLIM5 | 0.673433685 |
| TOMM70A | -0.664451234 |  | KLHDC9 | 0.673335126 |
| EEA1 | -0.663782851 |  | CILP2 | 0.672937139 |
| SLFN5 | -0.663597355 |  | TNXB | 0.672752988 |
| RAB31 | -0.663504801 |  | GALNT15 | 0.672587502 |
| GLRB | -0.663399316 |  | CDKN1B | 0.672384267 |
| TP53RK | -0.662497222 |  | KREMEN1 | 0.672207115 |
| ZC3H8 | -0.662227051 |  | IP6K1 | 0.672141741 |
| DNAJC12 | -0.662196542 |  | UNC5B | 0.672021149 |
| REV3L | -0.661641626 |  | VAPB | 0.671694576 |
| C5orf55 | -0.660767249 |  | PLA2R1 | 0.671636174 |
| GJD3 | -0.660663096 |  | CLDND1 | 0.67141429 |
| ACSL1 | -0.660119334 |  | IGFBP7 | 0.670915386 |
| SMIM4 | -0.660007024 |  | OCIAD2 | 0.670480598 |
| ETFB | -0.659211582 |  | GINS3 | 0.670305523 |
| COMMD8 | -0.658802292 |  | PLXNA2 | 0.669714007 |
| PSMB2 | -0.658774245 |  | HLA-C | 0.669681709 |
| CACNB2 | -0.658419964 |  | ZFP37 | 0.669174302 |
| SLC27A5 | -0.65790026 |  | BCL11A | 0.669171236 |
| TMEM150C | -0.656180111 |  | TMEM107 | 0.669170241 |
| ILF3 | -0.65568289 |  | TNNT3 | 0.668841311 |
| KIAA0141 | -0.655644171 |  | C7orf63 | 0.66870543 |
| EPHX1 | -0.655053201 |  | GPRASP1 | 0.668661604 |
| AIFM1 | -0.655016563 |  | GPR180 | 0.668487119 |
| MAPKAPK5-AS1 | -0.654189553 |  | C9orf72 | 0.668278609 |
| BRAF | -0.65408606 |  | FANK1 | 0.668000094 |
| AASS | -0.653997796 |  | CDS2 | 0.667805624 |
| LOC100134229 | -0.653755754 |  | WNK1 | 0.667345274 |
| SPIN3 | -0.653384244 |  | C7orf31 | 0.667283757 |
| POLD2 | -0.652823498 |  | AP4E1 | 0.66704526 |
| SHCBP1 | -0.652006201 |  | SMURF2 | 0.667038252 |
| AHI1 | -0.651615859 |  | TEX9 | 0.666890252 |
| GART | -0.651529363 |  | LPCAT2 | 0.666884603 |
| NOB1 | -0.651499912 |  | ZNF81 | 0.666458322 |
| MAD2L2 | -0.651247239 |  | MIRLET7BHG | 0.666439389 |
| MYO1F | -0.65013748 |  | MB21D2 | 0.666350727 |
| TTF2 | -0.649269481 |  | B4GALT4 | 0.666054725 |
| HPD | -0.648729342 |  | SLC26A6 | 0.665965364 |
| EIF5A | -0.648708599 |  | PTPN14 | 0.665911738 |
| FAR2 | -0.648647715 |  | DOK4 | 0.665802699 |
| DNPH1 | -0.648483264 |  | PELI2 | 0.665700914 |
| PCNX | -0.646868541 |  | NFAT5 | 0.665570957 |
| IQCH-AS1 | -0.64677904 |  | TGFB1I1 | 0.664842178 |
| NDUFAF4 | -0.646726475 |  | ATP6V1C1 | 0.664776235 |
| RPAIN | -0.646512972 |  | PTPN21 | 0.66445848 |
| C8orf82 | -0.64622481 |  | LOC100506844 | 0.664316794 |
| PUS1 | -0.646073155 |  | PAG1 | 0.663625997 |
| TBC1D16 | -0.64593987 |  | COX17 | 0.663594145 |
| EFS | -0.64564803 |  | TGFB2 | 0.663400463 |
| RFX5 | -0.645606992 |  | OSBPL3 | 0.663192368 |
| C10orf2 | -0.645413274 |  | INO80C | 0.662989153 |
| HELLS | -0.645206827 |  | CCNT2 | 0.662982591 |
| MME | -0.644667007 |  | PABPC4L | 0.662920872 |
| IFNGR1 | -0.642984508 |  | APBA2 | 0.66276767 |
| SGCG | -0.642606833 |  | BCOR | 0.662445625 |
| PRPS2 | -0.642488077 |  | CNOT6 | 0.661950997 |
| ACADS | -0.642464435 |  | CERS6 | 0.661848602 |
| NFIC | -0.641613656 |  | SLC8A1 | 0.661455786 |
| CCT4 | -0.641474091 |  | CBFB | 0.660737141 |
| FAM13A | -0.641077127 |  | CEP85L | 0.660270762 |
| MID1IP1 | -0.641057757 |  | PPM1K | 0.660239258 |
| CS | -0.641016634 |  | C1orf198 | 0.659814831 |
| TMCC1 | -0.639999207 |  | EFEMP1 | 0.659517889 |
| ZNF385B | -0.639925693 |  | WNT5A | 0.659024448 |
| PRKCA | -0.639855268 |  | KPNA5 | 0.658969792 |
| HIST1H2AC | -0.639538448 |  | LRRC1 | 0.65889296 |
| TMEM8B | -0.639523039 |  | NAPB | 0.658227773 |
| GCAT | -0.63951584 |  | SMIM7 | 0.657176807 |
| LOC100507395 | -0.639446533 |  | LYPLA2 | 0.657035986 |
| MRTO4 | -0.639358551 |  | FNBP1L | 0.65702969 |
| HCG11 | -0.6389203 |  | ARL10 | 0.656904408 |
| RTN4RL1 | -0.638226345 |  | BVES | 0.656131872 |
| ANKS6 | -0.637539469 |  | TRIM23 | 0.656128469 |
| LOC728769 | -0.636316922 |  | AKAP12 | 0.656118444 |
| RBPMS | -0.635354365 |  | AJUBA | 0.655952422 |
| LOC284926 | -0.63516014 |  | SEC14L2 | 0.655881506 |
| CRELD1 | -0.635020238 |  | APCDD1L-AS1 | 0.655570263 |
| ABR | -0.634828898 |  | WIPF1 | 0.655444144 |
| CMBL | -0.634613608 |  | PLEKHH2 | 0.655231853 |
| ANG | -0.633968682 |  | PLEKHA2 | 0.655188634 |
| SEPP1 | -0.633636122 |  | TAPBP | 0.654994218 |
| PGBD4 | -0.633362649 |  | PTPRF | 0.654433239 |
| LOC100287590 | -0.633210266 |  | FBXO32 | 0.654372 |
| MYD88 | -0.632694037 |  | SRSF11 | 0.65436081 |
| RPP25L | -0.632120698 |  | LOC100134259 | 0.654202287 |
| C16orf46 | -0.631629354 |  | ANKRD6 | 0.654182172 |
| CCT5 | -0.631535588 |  | CMTM4 | 0.653175134 |
| DCAF17 | -0.631494206 |  | MED13L | 0.653142202 |
| NUP153 | -0.631312496 |  | ABL2 | 0.65277238 |
| QARS | -0.631126398 |  | DEPTOR | 0.652669754 |
| DPT | -0.631098344 |  | HIVEP2 | 0.652132853 |
| AGPAT2 | -0.6308146 |  | CYP2U1 | 0.651974256 |
| NR4A3 | -0.630584319 |  | AKT2 | 0.651779173 |
| HEBP2 | -0.630025939 |  | ZFHX4 | 0.651538838 |
| SOBP | -0.629949184 |  | MEAF6 | 0.651348378 |
| CDC42EP4 | -0.629557355 |  | STK17A | 0.651143428 |
| ARHGAP5-AS1 | -0.629271535 |  | PAWR | 0.6508974 |
| DHDDS | -0.629094801 |  | CAB39L | 0.650801821 |
| CCDC113 | -0.628780925 |  | MGLL | 0.650440368 |
| PPA1 | -0.628724086 |  | LRP10 | 0.650095512 |
| MYBBP1A | -0.628645691 |  | LAMA2 | 0.649894792 |
| NDUFV3 | -0.62799947 |  | CEP97 | 0.649194646 |
| UTP14A | -0.62785351 |  | SECTM1 | 0.649100033 |
| IREB2 | -0.626859201 |  | PDSS2 | 0.647748989 |
| RASSF4 | -0.626363036 |  | FAM134C | 0.647515243 |
| STK32A | -0.626256456 |  | PMAIP1 | 0.647451179 |
| XPNPEP1 | -0.626204459 |  | SP110 | 0.646729724 |
| LOC441528 | -0.625878337 |  | ZXDC | 0.646633217 |
| ARL9 | -0.625710511 |  | ELAVL1 | 0.646379016 |
| TIPIN | -0.625583913 |  | LONRF2 | 0.646281431 |
| C6orf226 | -0.62546573 |  | DYNC1I1 | 0.645664088 |
| ME1 | -0.624886484 |  | GOLGB1 | 0.645364442 |
| FTCDNL1 | -0.624382674 |  | TMEM237 | 0.645124299 |
| KIF2C | -0.623095361 |  | SLC2A3 | 0.644466073 |
| DMRT2 | -0.622678781 |  | FAM199X | 0.643781845 |
| C9orf85 | -0.622342718 |  | ASPHD2 | 0.643325557 |
| RPS24 | -0.622152667 |  | CREM | 0.643244819 |
| RAPGEF1 | -0.621823584 |  | MTF2 | 0.642984422 |
| GLUL | -0.621768506 |  | MUM1 | 0.642788515 |
| ICAM3 | -0.620792314 |  | CRY2 | 0.64246012 |
| VASH2 | -0.620719931 |  | DST | 0.642353285 |
| LOC729680 | -0.620312648 |  | TSPY26P | 0.642348071 |
| HMGCS1 | -0.619141853 |  | SEMA3E | 0.642219638 |
| PRRT3 | -0.618671026 |  | BMPR1B | 0.642088723 |
| CIAO1 | -0.618377876 |  | MXRA5 | 0.642000754 |
| MRS2 | -0.617785248 |  | ETV1 | 0.641633637 |
| ISOC1 | -0.617736977 |  | MTR | 0.640718717 |
| PBK | -0.617537436 |  | PPP1R15A | 0.640681269 |
| PCAT6 | -0.617030381 |  | LRPAP1 | 0.640568397 |
| PDZRN3 | -0.617009463 |  | ATP6V0B | 0.640417093 |
| MTHFD1 | -0.616189866 |  | NASP | 0.640387207 |
| BBIP1 | -0.615082478 |  | CCP110 | 0.640262396 |
| LOC100506691 | -0.614940313 |  | CAND1 | 0.640010145 |
| PDCD5 | -0.614188169 |  | KIAA0247 | 0.639965546 |
| LIPA | -0.614113511 |  | F2RL1 | 0.639883011 |
| ST6GAL1 | -0.614087815 |  | TMEM170A | 0.639438619 |
| ALDH1L1 | -0.613834243 |  | GPSM2 | 0.639342926 |
| GALM | -0.613588847 |  | MVB12B | 0.639173223 |
| SPIRE1 | -0.613273298 |  | LMO4 | 0.639030706 |
| FOXM1 | -0.613207252 |  | MREG | 0.638974135 |
| RNASEH1 | -0.61264631 |  | IL4R | 0.638941576 |
| SNTB2 | -0.612509501 |  | TLE2 | 0.638554293 |
| RRS1 | -0.612207273 |  | NFKBIZ | 0.63851668 |
| MPP6 | -0.610998752 |  | CDC42BPB | 0.638325759 |
| PIK3R3 | -0.610987181 |  | CHD6 | 0.638004351 |
| ABCE1 | -0.610418814 |  | PAPPA | 0.637545797 |
| ACOX1 | -0.610238254 |  | THAP5 | 0.637099131 |
| FAM117B | -0.610163723 |  | IER5 | 0.63698267 |
| MPHOSPH9 | -0.609614239 |  | CDC37L1 | 0.636630493 |
| EXOC5 | -0.609503664 |  | TMEM41B | 0.636566626 |
| SEPHS1 | -0.608560626 |  | TCF4 | 0.636423898 |
| TARBP1 | -0.608493058 |  | TPM1 | 0.635899301 |
| SLC27A3 | -0.607719391 |  | TNC | 0.635508449 |
| TATDN2 | -0.607698557 |  | DDX58 | 0.635312336 |
| UBASH3B | -0.607621215 |  | RAI14 | 0.635168536 |
| SLC7A6 | -0.607538478 |  | DCAF7 | 0.634932897 |
| BHMT2 | -0.607194204 |  | NRAS | 0.634459619 |
| MLLT1 | -0.607159644 |  | EIF5A2 | 0.63425967 |
| DUS3L | -0.607045552 |  | PREX1 | 0.634054649 |
| TENM4 | -0.606986297 |  | ITFG1 | 0.633800795 |
| MRPS12 | -0.606819405 |  | MTMR6 | 0.63341519 |
| MCCC1 | -0.606656995 |  | PODNL1 | 0.632892902 |
| TTC28-AS1 | -0.606528387 |  | ZCCHC8 | 0.632755075 |
| MINA | -0.606273083 |  | DDAH1 | 0.632740435 |
| HSP90B1 | -0.605990222 |  | GPM6B | 0.63262795 |
| OGG1 | -0.60571581 |  | UBXN2A | 0.63260905 |
| TCTN3 | -0.605272163 |  | APPL2 | 0.632514961 |
| POLR1D | -0.605218485 |  | FBN2 | 0.632351833 |
| TBC1D2B | -0.60496542 |  | SPECC1 | 0.632246105 |
| CXCL12 | -0.604720667 |  | AVL9 | 0.632029283 |
| SOX9 | -0.604586842 |  | BHLHE41 | 0.632004497 |
| PDK1 | -0.604191057 |  | GADD45B | 0.631914378 |
| LINC00899 | -0.603328913 |  | CTDSPL | 0.63146107 |
| NR2F1-AS1 | -0.603042264 |  | GSK3B | 0.631237617 |
| LOC285812 | -0.602595342 |  | GALNT10 | 0.630697997 |
| C11orf57 | -0.602349773 |  | SEMA4F | 0.630526507 |
| THTPA | -0.602344883 |  | SLC35A2 | 0.63039543 |
| MBLAC2 | -0.602084561 |  | EXT1 | 0.630099181 |
| AMD1 | -0.601701625 |  | TCEAL3 | 0.629861344 |
| NNAT | -0.601274587 |  | CDC25B | 0.629545084 |
| TBX6 | -0.60107413 |  | STC1 | 0.629516198 |
| EMILIN1 | -0.600510276 |  | MAP2 | 0.629264488 |
| SLC5A6 | -0.600423622 |  | GSC | 0.628777829 |
| LYRM7 | -0.600095134 |  | AMOTL1 | 0.628770198 |
| SERPINB9 | -0.599287615 |  | ADAM10 | 0.628703066 |
| C7orf13 | -0.599271268 |  | PCNXL3 | 0.628127181 |
| RNF19A | -0.599227407 |  | FMO3 | 0.627712707 |
| ECSIT | -0.599193444 |  | CMPK2 | 0.627457959 |
| RUSC1-AS1 | -0.598878672 |  | TBX5 | 0.627262362 |
| SCYL2 | -0.597082098 |  | INSR | 0.627067926 |
| ETFA | -0.59689677 |  | SIGLEC17P | 0.626925437 |
| LRRC2 | -0.596879573 |  | MIOS | 0.626855732 |
| RAB9A | -0.596848571 |  | CDS1 | 0.626539966 |
| SSFA2 | -0.596652903 |  | PHIP | 0.626409256 |
| MSX1 | -0.596638013 |  | RUNX2 | 0.626221853 |
| PHKG1 | -0.596367518 |  | ZZZ3 | 0.626045234 |
| FRAS1 | -0.596081672 |  | SRRT | 0.626002344 |
| RUVBL1 | -0.595516069 |  | CDK14 | 0.625155513 |
| TSPAN15 | -0.595492969 |  | CTSS | 0.624846163 |
| RPS6KA5 | -0.595204428 |  | ETV5 | 0.624683108 |
| ZEB2 | -0.594977066 |  | CPEB4 | 0.624648588 |
| METTL2B | -0.592396671 |  | USP47 | 0.624271416 |
| SYNCRIP | -0.591780421 |  | HAND2 | 0.62418572 |
| RBMXL1 | -0.591695977 |  | ARID1B | 0.624166875 |
| DCUN1D4 | -0.591615175 |  | PCDH9 | 0.623144995 |
| CAMKMT | -0.591479118 |  | DBC1 | 0.622982645 |
| MRPL34 | -0.590715478 |  | ST5 | 0.622927258 |
| AZIN1 | -0.590006866 |  | CHMP1B | 0.622822673 |
| AGTR1 | -0.589924443 |  | VMP1 | 0.622764369 |
| ACACA | -0.589897141 |  | NYNRIN | 0.622595913 |
| EXOSC4 | -0.589830831 |  | DOCK9 | 0.622475161 |
| OGN | -0.589713845 |  | MYO16 | 0.622427241 |
| SLC19A1 | -0.589398634 |  | DTX3 | 0.622267431 |
| EMC7 | -0.589345553 |  | FLJ35024 | 0.622257912 |
| POLD3 | -0.589178317 |  | UNK | 0.622224685 |
| MEG3 | -0.588565524 |  | XAF1 | 0.622201046 |
| C10orf11 | -0.588328501 |  | ZNF44 | 0.62207565 |
| FHL1 | -0.586808687 |  | CRIM1 | 0.62197452 |
| RERE | -0.585851472 |  | KIF5C | 0.621886113 |
| C11orf1 | -0.585453511 |  | LOC202181 | 0.621790447 |
| RAD51AP1 | -0.585307715 |  | FAM43A | 0.621594801 |
| IRF4 | -0.58518063 |  | CDK6 | 0.6215374 |
| LOC100134361 | -0.585049617 |  | RHBDD2 | 0.621528184 |
|  |  |  | OTUD7B | 0.621446107 |
|  |  |  | ZNF805 | 0.621444253 |
|  |  |  | ATG5 | 0.621350582 |
|  |  |  | ZNF711 | 0.621190004 |
|  |  |  | PAGR1 | 0.62077526 |
|  |  |  | CTAGE5 | 0.62061786 |
|  |  |  | ALS2 | 0.620476242 |
|  |  |  | NKX3-1 | 0.619402778 |
|  |  |  | SRGN | 0.619387429 |
|  |  |  | ZCCHC6 | 0.618991554 |
|  |  |  | OSGIN2 | 0.618920137 |
|  |  |  | CALD1 | 0.618657089 |
|  |  |  | MFSD8 | 0.618422962 |
|  |  |  | ANKRD44 | 0.618377022 |
|  |  |  | EML2 | 0.618351835 |
|  |  |  | CSNK1G1 | 0.618319464 |
|  |  |  | UPP1 | 0.618169918 |
|  |  |  | CACFD1 | 0.618055473 |
|  |  |  | RICTOR | 0.617875294 |
|  |  |  | KLHL5 | 0.617627807 |
|  |  |  | PDCD1LG2 | 0.617386609 |
|  |  |  | SPA17 | 0.617351533 |
|  |  |  | NPTXR | 0.617229921 |
|  |  |  | KLF7 | 0.617166493 |
|  |  |  | SLC38A4 | 0.617150946 |
|  |  |  | ZNF37BP | 0.61707335 |
|  |  |  | GPATCH8 | 0.616809277 |
|  |  |  | DCUN1D2 | 0.61614014 |
|  |  |  | MERTK | 0.616110748 |
|  |  |  | DLX1 | 0.615967396 |
|  |  |  | PHACTR2 | 0.615470029 |
|  |  |  | LRP4 | 0.61546999 |
|  |  |  | IDS | 0.615436981 |
|  |  |  | UBL3 | 0.615297333 |
|  |  |  | KLF10 | 0.615266667 |
|  |  |  | TMF1 | 0.614997337 |
|  |  |  | FBN1 | 0.614831739 |
|  |  |  | TRAF6 | 0.614271155 |
|  |  |  | BTBD3 | 0.613811338 |
|  |  |  | STAM2 | 0.612957488 |
|  |  |  | PPP6C | 0.612600443 |
|  |  |  | LOC338620 | 0.612442803 |
|  |  |  | KIAA0319L | 0.612350707 |
|  |  |  | BCAP29 | 0.612166322 |
|  |  |  | MFAP2 | 0.612129346 |
|  |  |  | ENG | 0.612015689 |
|  |  |  | ASAP1 | 0.611865121 |
|  |  |  | SIPA1L1 | 0.611848248 |
|  |  |  | WDFY3 | 0.611612368 |
|  |  |  | SSH1 | 0.611437818 |
|  |  |  | TMEM99 | 0.611437442 |
|  |  |  | C19orf55 | 0.610538989 |
|  |  |  | CYB561D2 | 0.610415107 |
|  |  |  | RC3H2 | 0.609833619 |
|  |  |  | KCMF1 | 0.609726751 |
|  |  |  | NR0B1 | 0.60939911 |
|  |  |  | TMEM234 | 0.609333877 |
|  |  |  | TMEM41A | 0.60916133 |
|  |  |  | SLC12A6 | 0.608366674 |
|  |  |  | ZNF853 | 0.608231856 |
|  |  |  | SSX2IP | 0.60822765 |
|  |  |  | COL4A2 | 0.608084489 |
|  |  |  | CBL | 0.608018389 |
|  |  |  | SLC38A7 | 0.607894877 |
|  |  |  | DNAJC16 | 0.607827137 |
|  |  |  | CTNNB1 | 0.607746561 |
|  |  |  | LOC100506609 | 0.607267055 |
|  |  |  | PLXNB2 | 0.606824545 |
|  |  |  | SBNO1 | 0.606312198 |
|  |  |  | LCLAT1 | 0.6061646 |
|  |  |  | ERF | 0.606156993 |
|  |  |  | TMEM98 | 0.606053869 |
|  |  |  | CDKN1C | 0.606031377 |
|  |  |  | ENKD1 | 0.605484986 |
|  |  |  | ZNF540 | 0.605366481 |
|  |  |  | PCGF5 | 0.605362405 |
|  |  |  | EFEMP2 | 0.605324852 |
|  |  |  | SP3 | 0.605156243 |
|  |  |  | IQCK | 0.604460533 |
|  |  |  | TMOD3 | 0.603978529 |
|  |  |  | HERC5 | 0.603678806 |
|  |  |  | GPR137B | 0.603512059 |
|  |  |  | AQP9 | 0.603349412 |
|  |  |  | ATG2B | 0.60291928 |
|  |  |  | SNAPC3 | 0.602889147 |
|  |  |  | OPN1SW | 0.602419053 |
|  |  |  | EP300 | 0.602249385 |
|  |  |  | TMEM206 | 0.602239601 |
|  |  |  | ITGA4 | 0.601774745 |
|  |  |  | GTF2A1 | 0.6016489 |
|  |  |  | LOX | 0.60160502 |
|  |  |  | KIF3C | 0.601577687 |
|  |  |  | GLS | 0.601496461 |
|  |  |  | ITM2A | 0.601390338 |
|  |  |  | KIAA1217 | 0.601297004 |
|  |  |  | B4GALT3 | 0.601236633 |
|  |  |  | NEXN | 0.601031006 |
|  |  |  | CTDSP2 | 0.600958326 |
|  |  |  | TMEM245 | 0.600903362 |
|  |  |  | TAF9B | 0.600616094 |
|  |  |  | SUV420H1 | 0.600543558 |
|  |  |  | NAA40 | 0.600425509 |
|  |  |  | ZSCAN30 | 0.600421207 |
|  |  |  | FIBIN | 0.600396189 |
|  |  |  | SEC63 | 0.600209218 |
|  |  |  | PRRC2C | 0.599951164 |
|  |  |  | GPRC5B | 0.599691047 |
|  |  |  | SLK | 0.599214545 |
|  |  |  | CLCN5 | 0.599202872 |
|  |  |  | ZNF251 | 0.599084191 |
|  |  |  | HAGH | 0.598907341 |
|  |  |  | MAP4K5 | 0.598725948 |
|  |  |  | LATS2 | 0.598713305 |
|  |  |  | RAPH1 | 0.598563415 |
|  |  |  | IPO9 | 0.598489089 |
|  |  |  | GALNT2 | 0.597631771 |
|  |  |  | ZNF148 | 0.597557861 |
|  |  |  | ADAMTSL1 | 0.597465069 |
|  |  |  | SLC7A8 | 0.597408969 |
|  |  |  | TRO | 0.597287854 |
|  |  |  | MXRA7 | 0.597003553 |
|  |  |  | PLA2G4C | 0.596913799 |
|  |  |  | SCYL3 | 0.596834948 |
|  |  |  | TSPAN6 | 0.596825308 |
|  |  |  | LRRC16A | 0.596444523 |
|  |  |  | HECTD4 | 0.595995617 |
|  |  |  | RBBP4 | 0.595936823 |
|  |  |  | DAAM1 | 0.595725882 |
|  |  |  | MFAP3 | 0.595710015 |
|  |  |  | PALLD | 0.595643126 |
|  |  |  | ROR1 | 0.594940502 |
|  |  |  | NOTCH2 | 0.594734216 |
|  |  |  | SMCR8 | 0.594449531 |
|  |  |  | PPP2R5B | 0.594405216 |
|  |  |  | BRWD1 | 0.594392312 |
|  |  |  | COL6A1 | 0.594092712 |
|  |  |  | MAFK | 0.593937398 |
|  |  |  | ZNF396 | 0.593376076 |
|  |  |  | LZTFL1 | 0.593284501 |
|  |  |  | SYNJ1 | 0.593282125 |
|  |  |  | GABARAPL1 | 0.593184719 |
|  |  |  | TET3 | 0.592835163 |
|  |  |  | PCGF2 | 0.592685136 |
|  |  |  | CNPY4 | 0.592302484 |
|  |  |  | KIAA1644 | 0.592295931 |
|  |  |  | PMEPA1 | 0.591042769 |
|  |  |  | LSM14A | 0.590957642 |
|  |  |  | TRIO | 0.590892366 |
|  |  |  | COQ7 | 0.590706397 |
|  |  |  | VPS37B | 0.590679613 |
|  |  |  | KMT2A | 0.590573705 |
|  |  |  | CXCL3 | 0.590500618 |
|  |  |  | TMEM154 | 0.590262844 |
|  |  |  | IGF1R | 0.59018122 |
|  |  |  | C19orf26 | 0.589934285 |
|  |  |  | TBX5-AS1 | 0.589813741 |
|  |  |  | SUPT6H | 0.589744687 |
|  |  |  | MALT1 | 0.589401527 |
|  |  |  | NDUFB2 | 0.589379208 |
|  |  |  | HEY1 | 0.589334812 |
|  |  |  | ANKRD36B | 0.589317983 |
|  |  |  | CYTH3 | 0.589302673 |
|  |  |  | MPZL1 | 0.58880226 |
|  |  |  | MCFD2 | 0.588471301 |
|  |  |  | LOC100996615 | 0.587859125 |
|  |  |  | BRWD3 | 0.587801705 |
|  |  |  | MAP6 | 0.587545411 |
|  |  |  | TMEM160 | 0.587465551 |
|  |  |  | DCBLD2 | 0.587352454 |
|  |  |  | STARD10 | 0.58707438 |
|  |  |  | RUFY3 | 0.586932125 |
|  |  |  | COL4A5 | 0.586819326 |
|  |  |  | CYLD | 0.586563716 |
|  |  |  | PRKAA1 | 0.586483622 |
|  |  |  | NFATC2IP | 0.586379742 |
|  |  |  | PRSS23 | 0.586071195 |
|  |  |  | TMED5 | 0.585944367 |
|  |  |  | PTPN9 | 0.585801813 |
|  |  |  | DPYSL3 | 0.585513733 |
|  |  |  | ATP13A2 | 0.585372931 |
|  |  |  | DGKE | 0.585201709 |
|  |  |  | SLC7A5 | 0.585177165 |
|  |  |  | SLC38A2 | 0.585027685 |
