# Supplementary material for: MYC Is an Early Response Regulator of Human Adipogenesis in Adipose Stem Cells
Source: PLoS One. 2014 Dec 1;9(12):e114133. doi: 10.1371/journal.pone.0114133 (PMC4250176; doi:10.1371/journal.pone.0114133)
Supplement: Table S2 — Gene expression fold-change values for s91-29 MYC siRNA significant genes. (DOCX) [file pone.0114133.s005.docx]

**Supplementary Table 2.** Gene expression fold-change values for s91-29 *MYC* siRNA significant genes.

| s91-29 Down | Log2 Fold Change |  | s91-29 Up | Log2 Fold Change |
| --- | --- | --- | --- | --- |
| ASPN | -3.513080404 |  | TMEM255A | 4.451133488 |
| GPD1 | -3.441630551 |  | KRTAP1-5 | 3.886418043 |
| LGALS12 | -3.141750089 |  | HCP5 | 3.76266179 |
| PCK1 | -3.029396028 |  | BEX1 | 3.32671887 |
| LPL | -2.953261031 |  | NRK | 3.114021656 |
| THRSP | -2.910129102 |  | CPA4 | 3.060401377 |
| LOC100509635 | -2.827655322 |  | PCDH10 | 3.049415955 |
| IGF1 | -2.764809298 |  | SST | 2.812428078 |
| ARPC1B | -2.701278922 |  | IFI27 | 2.769867424 |
| MT1M | -2.556252125 |  | IL7R | 2.768788765 |
| ABAT | -2.529024906 |  | TMEFF2 | 2.582941477 |
| ADIPOQ | -2.510150431 |  | TMEM2 | 2.452882197 |
| MAMDC2 | -2.501706555 |  | AGTRAP | 2.379696486 |
| GPNMB | -2.46710573 |  | OLAH | 2.354141101 |
| ELOVL6 | -2.444378868 |  | SLC12A8 | 2.32763165 |
| UCP2 | -2.341444995 |  | CECR1 | 2.293722194 |
| SMOC2 | -2.312032739 |  | PAQR5 | 2.289025801 |
| VASH2 | -2.287595919 |  | LPXN | 2.270394307 |
| PLIN1 | -2.284919215 |  | ANTXR1 | 2.265857775 |
| EVI2A | -2.277022677 |  | CLDND1 | 2.260434094 |
| OMD | -2.226276655 |  | RRAGD | 2.239745221 |
| LIMS1 | -2.154286029 |  | CYFIP2 | 2.235255066 |
| RPUSD3 | -2.153932803 |  | HIST3H2A | 2.20652812 |
| CA2 | -2.144838745 |  | PFKFB4 | 2.203152 |
| PLXDC2 | -2.13266033 |  | FPR1 | 2.178796742 |
| OMG | -2.10323945 |  | LOC100507463 | 2.178240066 |
| PTGFR | -2.100976392 |  | HIST1H3H | 2.137765441 |
| KCND3 | -2.095088449 |  | GPR39 | 2.062060888 |
| ITIH5 | -2.073904574 |  | BEX2 | 2.041581628 |
| LIPG | -2.050301127 |  | SLC7A7 | 2.01427909 |
| EVI2B | -2.035057489 |  | RELN | 2.007537202 |
| TMEM150C | -2.029168906 |  | GGH | 2.00636824 |
| PLA2G16 | -2.013119914 |  | MLLT11 | 2.005704201 |
| REPS2 | -2.012749626 |  | MCOLN3 | 1.962486482 |
| BLOC1S6 | -2.009599516 |  | SSTR1 | 1.95708204 |
| KDSR | -2.00543458 |  | SEC11C | 1.940517074 |
| STAU2 | -2.003518671 |  | SPINK6 | 1.940403225 |
| IP6K2 | -1.985338241 |  | PTPLAD2 | 1.939946562 |
| UQCR10 | -1.977479244 |  | ANKRD33B | 1.934944699 |
| RASD1 | -1.962311092 |  | SPP1 | 1.934511249 |
| BCL2 | -1.956851906 |  | LONRF3 | 1.929341799 |
| IL13RA1 | -1.938337715 |  | QPCT | 1.903441699 |
| SESN3 | -1.920848888 |  | HTATIP2 | 1.900366986 |
| BDH1 | -1.89808895 |  | IL1B | 1.898739167 |
| OGN | -1.893185896 |  | BST2 | 1.897757448 |
| DUSP4 | -1.869520439 |  | ENO2 | 1.894727751 |
| CEBPA | -1.863575015 |  | PAMR1 | 1.888027805 |
| AASS | -1.855881343 |  | C21orf2 | 1.883031067 |
| EDNRB | -1.847910566 |  | FKBP1B | 1.882686556 |
| ZNF117 | -1.847593352 |  | LEPREL1 | 1.87633419 |
| ITGA7 | -1.838822397 |  | ZNF789 | 1.874974576 |
| CPSF2 | -1.83868744 |  | HLA-DPA1 | 1.872470638 |
| TSPAN15 | -1.834326384 |  | LRRC15 | 1.86910953 |
| ALDH6A1 | -1.83386407 |  | MAP2 | 1.867982518 |
| MBNL3 | -1.829838805 |  | OLFML1 | 1.861810595 |
| NEBL | -1.821543543 |  | BEX5 | 1.860382672 |
| WDR35 | -1.813835134 |  | OCIAD2 | 1.844889648 |
| MAP2K6 | -1.800369898 |  | PMAIP1 | 1.842542018 |
| MARCKSL1 | -1.791318371 |  | CD1D | 1.835134406 |
| SOX9 | -1.783812806 |  | RAB39B | 1.827411839 |
| MXD1 | -1.782291249 |  | LYN | 1.819081677 |
| ITGA2 | -1.777297211 |  | TREM1 | 1.814905705 |
| MTSS1 | -1.773928223 |  | KIAA1598 | 1.812294399 |
| C4orf32 | -1.768622452 |  | MICB | 1.804968124 |
| ERGIC2 | -1.75798802 |  | BHMT2 | 1.803705477 |
| DHTKD1 | -1.757594516 |  | ATP6V0E2 | 1.797033362 |
| COMP | -1.756559335 |  | CXCL1 | 1.795208538 |
| NPR1 | -1.756472868 |  | MX1 | 1.79229839 |
| ZNF704 | -1.756366822 |  | LOC100288911 | 1.773797837 |
| PLIN4 | -1.755240755 |  | NHLRC3 | 1.753417211 |
| RHOU | -1.741209673 |  | ARSI | 1.739716219 |
| CYB5A | -1.741146163 |  | KCNQ3 | 1.733658606 |
| SCRG1 | -1.741119461 |  | ALDH1A1 | 1.725310623 |
| IGFBP5 | -1.734511514 |  | CNR1 | 1.719021708 |
| RIMS2 | -1.727329799 |  | LINC00857 | 1.718674112 |
| SLU7 | -1.726896663 |  | GIMAP2 | 1.712812568 |
| FAM65B | -1.724620411 |  | TSPAN12 | 1.704570111 |
| MCF2L | -1.723700921 |  | ADAM19 | 1.704027481 |
| ANKDD1A | -1.715993997 |  | BTN3A2 | 1.701545211 |
| CXCL5 | -1.715296325 |  | SHISA2 | 1.700888644 |
| ARG2 | -1.715155597 |  | CLGN | 1.691445241 |
| SLMO2 | -1.707092685 |  | NUPL1 | 1.68638755 |
| TMEM170B | -1.706166036 |  | SUSD1 | 1.684647165 |
| MAFB | -1.703354426 |  | GDNF | 1.679928656 |
| KCNK2 | -1.702840574 |  | MGARP | 1.678991878 |
| CA3 | -1.699189719 |  | ZEB2 | 1.676116396 |
| PRSS23 | -1.69447035 |  | ZNF273 | 1.675154869 |
| RUSC1-AS1 | -1.688729731 |  | C17orf100 | 1.664081547 |
| LBR | -1.684405548 |  | IL8 | 1.649184611 |
| CLCN4 | -1.677760911 |  | TSPY26P | 1.639179197 |
| PLSCR4 | -1.672319682 |  | STAMBPL1 | 1.635977761 |
| PCYT2 | -1.670503986 |  | COLEC12 | 1.63579275 |
| CILP | -1.655451955 |  | TMEM8A | 1.632826936 |
| PDE1A | -1.65208322 |  | ABCC3 | 1.618026073 |
| COL6A2 | -1.649799245 |  | PLA2G15 | 1.615799329 |
| PDE3B | -1.648946508 |  | PF4V1 | 1.60609932 |
| TPR | -1.646563831 |  | PSG5 | 1.60522427 |
| GTF3A | -1.644055999 |  | HERC6 | 1.605021275 |
| RASL11B | -1.643760123 |  | LOC285835 | 1.602429594 |
| CMKLR1 | -1.642411742 |  | ALDH3B1 | 1.599789056 |
| SMOC1 | -1.640244642 |  | HINT1 | 1.597257857 |
| C2CD2 | -1.631279834 |  | RRAD | 1.595024049 |
| C2orf88 | -1.62647292 |  | AKR1C1 | 1.586372187 |
| ZNF436 | -1.623088872 |  | SERTAD4-AS1 | 1.585297941 |
| TLCD2 | -1.621314103 |  | PPAP2C | 1.58455221 |
| LAMA2 | -1.618930427 |  | IKZF2 | 1.584447305 |
| DPYSL2 | -1.614158072 |  | C15orf48 | 1.583992206 |
| NANOS1 | -1.613394355 |  | DCP2 | 1.581300162 |
| SNX10 | -1.610094747 |  | DDIT4L | 1.576702377 |
| DESI1 | -1.609590302 |  | ULBP2 | 1.576308734 |
| LRRN4CL | -1.609118331 |  | OXR1 | 1.568939961 |
| ROR1 | -1.605255414 |  | CPVL | 1.56577851 |
| PAPOLA | -1.605063431 |  | TBX5-AS1 | 1.560923397 |
| RPA1 | -1.602455907 |  | NAAA | 1.549828529 |
| IAH1 | -1.594965486 |  | TGOLN2 | 1.548932291 |
| ATG4A | -1.591586205 |  | LRP10 | 1.54845692 |
| GLUD2 | -1.591318814 |  | SCG5 | 1.540893226 |
| LPIN1 | -1.586788263 |  | NEFM | 1.537230543 |
| TMEM30B | -1.580320431 |  | SSU72 | 1.528856986 |
| CITED1 | -1.580033588 |  | LOC158402 | 1.528277602 |
| AMMECR1 | -1.579998915 |  | LY6K | 1.524675779 |
| KLF15 | -1.5781333 |  | PPA2 | 1.522481484 |
| LPHN1 | -1.572299755 |  | CD99P1 | 1.520520614 |
| SFRP2 | -1.571992713 |  | ARL6IP6 | 1.516454391 |
| CAMK2D | -1.561603689 |  | ERN1 | 1.513940054 |
| SEPP1 | -1.560407738 |  | TRERF1 | 1.512686037 |
| PCSK9 | -1.559716797 |  | MIR210HG | 1.511846617 |
| GCAT | -1.557736911 |  | HTRA3 | 1.507851346 |
| PAPPA2 | -1.54962842 |  | NEU1 | 1.503280316 |
| PCSK1 | -1.548771187 |  | ZMIZ1-AS1 | 1.499104527 |
| ADAMTS12 | -1.548285612 |  | STAR | 1.496830515 |
| AKAP7 | -1.545776224 |  | DUSP5 | 1.496100352 |
| MYH10 | -1.54572634 |  | NALCN | 1.495952832 |
| ADAM17 | -1.545725247 |  | GCA | 1.495358767 |
| SORT1 | -1.536106137 |  | LOC100287015 | 1.493426076 |
| DNMT3A | -1.535669792 |  | LOC100129550 | 1.492689922 |
| DBT | -1.535644942 |  | ZNF600 | 1.489349315 |
| DOCK11 | -1.533212756 |  | LOC642852 | 1.488775642 |
| ABCD2 | -1.521230671 |  | SEL1L3 | 1.487459804 |
| TOB2 | -1.518245512 |  | PSG4 | 1.483660769 |
| CACNB2 | -1.514768009 |  | PFKFB2 | 1.479257962 |
| IL21R | -1.514132838 |  | HRCT1 | 1.478405112 |
| SHMT1 | -1.513026587 |  | SBF2-AS1 | 1.477139033 |
| ANK2 | -1.51064543 |  | RAB3D | 1.47462002 |
| EMX2OS | -1.506498289 |  | RIT1 | 1.472402641 |
| NFIC | -1.506028109 |  | LAPTM5 | 1.465460947 |
| SCD | -1.502003992 |  | LOC100507507 | 1.460793576 |
| GXYLT2 | -1.500357175 |  | TSLP | 1.46040497 |
| FAM134B | -1.494866386 |  | BEND7 | 1.45181014 |
| CYP19A1 | -1.490375316 |  | SGPL1 | 1.448882453 |
| SHPRH | -1.484989122 |  | PAN3-AS1 | 1.446695146 |
| MTMR6 | -1.482431905 |  | C9orf72 | 1.445749727 |
| MGP | -1.480308159 |  | CYP4V2 | 1.445642689 |
| RNF125 | -1.475841727 |  | NCEH1 | 1.441648635 |
| NEXN | -1.475824203 |  | LOC100130938 | 1.438773108 |
| CDON | -1.475164915 |  | FBXO32 | 1.434573798 |
| HMGCS1 | -1.472598745 |  | THBS1 | 1.432691459 |
| NPEPPS | -1.472434509 |  | BTN3A3 | 1.430070166 |
| GPAM | -1.470413553 |  | LEPREL2 | 1.429452064 |
| HAVCR2 | -1.468164048 |  | SAP30L | 1.429277998 |
| STYX | -1.465795178 |  | PIP4K2C | 1.426467185 |
| LOC729680 | -1.46424443 |  | C10orf114 | 1.425956602 |
| GLDN | -1.458702448 |  | GRIA1 | 1.424656847 |
| CDKN1C | -1.458523398 |  | LOC100128822 | 1.4200899 |
| UHRF1BP1 | -1.454851744 |  | CTSS | 1.419922556 |
| RAB33A | -1.453486569 |  | ATOH8 | 1.417812805 |
| AVPR1A | -1.45130007 |  | HLA-DPB1 | 1.41773566 |
| SLCO4C1 | -1.450657061 |  | CXCL2 | 1.415313372 |
| PKIA | -1.449698449 |  | LOC728769 | 1.413697342 |
| NFIB | -1.445493747 |  | ZNF451 | 1.411484472 |
| MTR | -1.445410984 |  | C2orf68 | 1.409959043 |
| FAF2 | -1.44235679 |  | STEAP4 | 1.407449047 |
| CELF2 | -1.437829113 |  | ADAMDEC1 | 1.40587119 |
| ACSS2 | -1.437521257 |  | ANKRD1 | 1.405824011 |
| TMEM97 | -1.435838291 |  | ATPAF1 | 1.403812862 |
| HRASLS5 | -1.419248526 |  | SEMA7A | 1.402342707 |
| PAPSS2 | -1.414780582 |  | TUFT1 | 1.402027259 |
| C16orf46 | -1.413347119 |  | HHAT | 1.399913169 |
| ACAT2 | -1.409714642 |  | IFI6 | 1.396570001 |
| ICK | -1.408009134 |  | HIST1H1C | 1.394555597 |
| EGR3 | -1.405910282 |  | IL13RA2 | 1.394084396 |
| HS3ST2 | -1.401375243 |  | HERC5 | 1.392433508 |
| GK5 | -1.399789415 |  | TACSTD2 | 1.391112637 |
| CBS | -1.395798635 |  | CNTNAP1 | 1.390414331 |
| CREBRF | -1.394616274 |  | ATL1 | 1.389849079 |
| NETO2 | -1.39067792 |  | CHEK2 | 1.382770781 |
| SLC18B1 | -1.38827941 |  | BLVRB | 1.378277014 |
| SAMHD1 | -1.386182327 |  | PSTPIP2 | 1.378182914 |
| LSAMP | -1.382972237 |  | PPAPDC1A | 1.375578912 |
| ACVR1C | -1.382868825 |  | FRMD5 | 1.369175285 |
| CACNA2D1 | -1.381688558 |  | SLC16A2 | 1.366488493 |
| LRP3 | -1.381554778 |  | MAGEL2 | 1.365648897 |
| SYNC | -1.372043986 |  | FKBP11 | 1.352594265 |
| DDX18 | -1.371192304 |  | AADAC | 1.351769433 |
| BAIAP2-AS1 | -1.37072535 |  | MMP3 | 1.35044226 |
| PPP2R5E | -1.370466107 |  | JAM2 | 1.349120768 |
| RERG | -1.369725749 |  | DPP7 | 1.347070787 |
| IFNAR1 | -1.36874914 |  | PNKD | 1.346719189 |
| KANSL1L | -1.359840822 |  | NAA38 | 1.343723967 |
| TXLNG | -1.358447364 |  | LY96 | 1.340271369 |
| NFIA | -1.357667713 |  | TSPAN13 | 1.335556796 |
| KLHL20 | -1.353788163 |  | KIAA1967 | 1.335073984 |
| DCUN1D1 | -1.352344675 |  | CCL2 | 1.334468915 |
| PLEKHA3 | -1.348607686 |  | TGFB3 | 1.333682679 |
| CSAD | -1.346877282 |  | TAP1 | 1.332334561 |
| RPSA | -1.345869736 |  | PLAUR | 1.332129709 |
| AHCTF1 | -1.345747247 |  | TMEM106A | 1.33108844 |
| OIP5-AS1 | -1.344870669 |  | LOC100506377 | 1.330370311 |
| LYPLAL1 | -1.341243435 |  | SLC38A5 | 1.330220814 |
| RFK | -1.340727286 |  | SLC46A3 | 1.32976107 |
| FAM117B | -1.339714927 |  | INPP5F | 1.329504215 |
| XPNPEP2 | -1.336931007 |  | ANKRD6 | 1.32815054 |
| RAB40B | -1.331661864 |  | HPGD | 1.327775403 |
| LNPEP | -1.331539934 |  | ZNF738 | 1.327401485 |
| PDK4 | -1.330316645 |  | OAS1 | 1.325569054 |
| COQ3 | -1.330167652 |  | CEP41 | 1.322374594 |
| CDCA7L | -1.329585299 |  | TOLLIP-AS1 | 1.322364966 |
| ADAM12 | -1.328448088 |  | VAMP8 | 1.319705523 |
| LMO4 | -1.327848178 |  | CARD16 | 1.318542101 |
| PDP2 | -1.327807839 |  | LOC100287387 | 1.315292132 |
| VSIG10 | -1.327082712 |  | CXCL6 | 1.314891717 |
| COL21A1 | -1.322529863 |  | LINC00537 | 1.312108568 |
| TMTC3 | -1.322410626 |  | C7orf10 | 1.311134011 |
| BASP1 | -1.321770337 |  | TMEM205 | 1.309970654 |
| PHF20L1 | -1.318074443 |  | PLCB1 | 1.307232508 |
| FKBP14 | -1.31790343 |  | MIB2 | 1.305476148 |
| ISM1 | -1.317569605 |  | LOC100506713 | 1.304884497 |
| VBP1 | -1.317509935 |  | RABL6 | 1.304098093 |
| PTP4A2 | -1.316529079 |  | ARL10 | 1.302667353 |
| MFAP2 | -1.315122828 |  | RNF213 | 1.299075025 |
| GPER | -1.313781332 |  | PDK1 | 1.297844753 |
| MLEC | -1.313517129 |  | SLC36A4 | 1.295357181 |
| CDK14 | -1.312663427 |  | BRD7 | 1.294996484 |
| SIX1 | -1.309982218 |  | TMEM182 | 1.292642002 |
| LOC389906 | -1.306258987 |  | APOB | 1.291703728 |
| GABPA | -1.30622087 |  | SPAG4 | 1.29140453 |
| MT1E | -1.30491664 |  | C3orf55 | 1.286344338 |
| C12orf4 | -1.301611912 |  | MPZL3 | 1.28581561 |
| KIAA0226L | -1.300724456 |  | MFI2 | 1.285006263 |
| SCARA3 | -1.30034129 |  | LAMC2 | 1.28337588 |
| PALMD | -1.300150329 |  | ADAMTS5 | 1.281870524 |
| FKBP5 | -1.296481318 |  | TMEM25 | 1.27355431 |
| P4HA3 | -1.296036689 |  | KIAA1024 | 1.272194503 |
| ACACB | -1.295590599 |  | CACNA2D3 | 1.271271806 |
| PARD6G | -1.292532958 |  | TNFRSF1B | 1.270884631 |
| RAC3 | -1.284163331 |  | STAT4 | 1.269420597 |
| GPC4 | -1.28369445 |  | TNFSF4 | 1.266486405 |
| SLC25A18 | -1.283152178 |  | SDSL | 1.265854455 |
| MOB1A | -1.282730474 |  | GSAP | 1.259152805 |
| RNF144A | -1.279914812 |  | LOC729970 | 1.25843125 |
| HTR2A | -1.279299584 |  | C5orf46 | 1.257897718 |
| C5orf51 | -1.277794608 |  | FLJ32255 | 1.257664523 |
| HSPB6 | -1.27720274 |  | FAM24B | 1.255910751 |
| PIBF1 | -1.274541353 |  | LOXL1-AS1 | 1.255655497 |
| ATG10 | -1.271383526 |  | OAS3 | 1.253661998 |
| SDF2 | -1.27009593 |  | SLC35A2 | 1.251647391 |
| HLTF | -1.269860143 |  | LMCD1 | 1.250710967 |
| SPAG1 | -1.269210249 |  | MTHFD2L | 1.247449689 |
| STAG2 | -1.267166684 |  | EPSTI1 | 1.247431376 |
| CHI3L2 | -1.265112055 |  | SSX2IP | 1.247012619 |
| TNS1 | -1.263380582 |  | IL15 | 1.24348629 |
| PPP1R1A | -1.262717509 |  | FAM179A | 1.243007877 |
| PPP5D1 | -1.259538768 |  | SEC22C | 1.240556854 |
| TLE2 | -1.253019957 |  | CCL28 | 1.240190141 |
| KBTBD11 | -1.252021024 |  | APOL1 | 1.237642628 |
| ZNF44 | -1.250242364 |  | GLI3 | 1.234478616 |
| CKAP2 | -1.250112961 |  | CHD9 | 1.234393462 |
| FGFBP2 | -1.248661501 |  | C20orf112 | 1.23276453 |
| GBAS | -1.248417337 |  | TRABD2A | 1.228974457 |
| UTP23 | -1.246392853 |  | STX3 | 1.228950415 |
| INHBB | -1.246040587 |  | PPM1H | 1.228924259 |
| CA12 | -1.244869146 |  | TTLL7 | 1.228517439 |
| C6 | -1.244297182 |  | KYNU | 1.224506119 |
| IVD | -1.243822658 |  | ZDHHC2 | 1.221750485 |
| FYN | -1.241456811 |  | RAB9B | 1.216968743 |
| GPD1L | -1.238510636 |  | GCHFR | 1.215920077 |
| ANKFY1 | -1.237538884 |  | SDC1 | 1.214153377 |
| HDAC4 | -1.234006883 |  | MGC45800 | 1.213579451 |
| LOC728730 | -1.232336209 |  | TAP2 | 1.213333971 |
| LOC441528 | -1.230909381 |  | BAALC | 1.211585015 |
| TOR1B | -1.228116491 |  | P2RX4 | 1.211102922 |
| FKBP7 | -1.228047553 |  | HIST2H2BE | 1.210217043 |
| PDE4B | -1.227177529 |  | LMBR1L | 1.20278941 |
| MRPL19 | -1.226684244 |  | GPR157 | 1.201597363 |
| B3GALNT1 | -1.226145673 |  | PSMB9 | 1.201146885 |
| MIB1 | -1.223727506 |  | RMND5A | 1.197062902 |
| CDNF | -1.219968965 |  | FOXQ1 | 1.196828114 |
| KPNB1 | -1.219057553 |  | CD40 | 1.196491024 |
| CADM3 | -1.218009971 |  | EPG5 | 1.19563329 |
| LAMB1 | -1.217160478 |  | IL6R | 1.193519606 |
| KIF7 | -1.216470805 |  | KIAA0101 | 1.191090084 |
| PODN | -1.214833824 |  | CHCHD7 | 1.190129073 |
| ANKIB1 | -1.213963358 |  | HNRNPU | 1.189427578 |
| ADAM9 | -1.213926562 |  | CADPS2 | 1.186519028 |
| SPATA9 | -1.213782323 |  | HMGCS2 | 1.186511184 |
| PC | -1.208614383 |  | PPT1 | 1.186320618 |
| SOX4 | -1.206223586 |  | TLN1 | 1.18575223 |
| COX7B | -1.205258665 |  | SV2A | 1.185684852 |
| CSGALNACT1 | -1.203692024 |  | FGF1 | 1.18432133 |
| PTGER3 | -1.203189465 |  | PINLYP | 1.183759854 |
| TENM4 | -1.203104671 |  | UQCRB | 1.183434327 |
| ZBED3 | -1.200518833 |  | MYEF2 | 1.183177718 |
| GYG2 | -1.200350396 |  | DLGAP1-AS2 | 1.183054039 |
| SLC22A3 | -1.197728716 |  | LINC00865 | 1.182562699 |
| B4GALT5 | -1.196450064 |  | P4HTM | 1.181978616 |
| ZNRF3 | -1.19359265 |  | PCDH19 | 1.180868516 |
| FAM206A | -1.192687362 |  | ASPH | 1.177910544 |
| KIF26B | -1.192670858 |  | SYNGR2 | 1.175229781 |
| VPS4B | -1.191894317 |  | LPCAT2 | 1.173529826 |
| MRAP | -1.191270854 |  | WDR86 | 1.173366062 |
| MESP1 | -1.188660334 |  | ASH1L-AS1 | 1.171770714 |
| SORBS1 | -1.184038382 |  | CRELD1 | 1.171486875 |
| CDH12 | -1.183197936 |  | TTC13 | 1.170593228 |
| MPHOSPH9 | -1.181814154 |  | CH25H | 1.168219912 |
| SLMAP | -1.181482013 |  | TBXA2R | 1.167262299 |
| RWDD4 | -1.17904329 |  | PLD6 | 1.166353477 |
| BEND6 | -1.177067311 |  | PVRL2 | 1.162838497 |
| C22orf39 | -1.176963494 |  | FLVCR1 | 1.162715546 |
| F3 | -1.176810054 |  | SYCE1L | 1.159995622 |
| STMN2 | -1.175607441 |  | P4HA1 | 1.159875388 |
| OSBPL8 | -1.175249362 |  | SLC17A5 | 1.157633279 |
| TRDMT1 | -1.173094873 |  | C20orf194 | 1.15676796 |
| ALCAM | -1.17213803 |  | ZDHHC12 | 1.156552462 |
| GNAQ | -1.171875307 |  | ITGB3 | 1.156472861 |
| SATB1 | -1.16978021 |  | TMEM51 | 1.155510315 |
| MFAP4 | -1.169148188 |  | C3orf58 | 1.155187585 |
| C16orf87 | -1.169061173 |  | ZNF630 | 1.153459839 |
| RNF44 | -1.168404592 |  | RARRES1 | 1.152563626 |
| FBLN1 | -1.167701456 |  | CYB5R2 | 1.151570916 |
| DSG2 | -1.167298944 |  | PRSS35 | 1.149584007 |
| IREB2 | -1.166869561 |  | DCHS1 | 1.149335423 |
| TXNDC12 | -1.165929175 |  | TNXB | 1.147982548 |
| FAM173B | -1.165865776 |  | LOC730101 | 1.14749953 |
| SDC3 | -1.163630093 |  | PHLDA1 | 1.146611678 |
| ST6GALNAC5 | -1.159382482 |  | SEPT6 | 1.144051161 |
| EIF3H | -1.156399233 |  | LOC93622 | 1.143678029 |
| XPO7 | -1.155711783 |  | PSMB8 | 1.143108544 |
| PRKAR2B | -1.153690148 |  | MOXD1 | 1.141072727 |
| LYRM2 | -1.15251994 |  | C7orf31 | 1.138549537 |
| TAF9B | -1.151792769 |  | ENTPD6 | 1.136377847 |
| ABHD3 | -1.151059736 |  | MALL | 1.135474303 |
| CPB1 | -1.149525959 |  | LOC389834 | 1.135238018 |
| FBN1 | -1.149511551 |  | DMRT2 | 1.131138132 |
| VIT | -1.147367741 |  | ISG15 | 1.130496655 |
| ZFP36L2 | -1.146076362 |  | DNAJC12 | 1.130227861 |
| RBMS3 | -1.143498502 |  | SYTL2 | 1.130161009 |
| FASN | -1.141978697 |  | PDIA5 | 1.129175331 |
| QKI | -1.141463039 |  | HDAC9 | 1.128450175 |
| MRO | -1.141413595 |  | CPEB2 | 1.127831995 |
| HCFC2 | -1.1407491 |  | VRK3 | 1.127496592 |
| STON2 | -1.140504595 |  | HERC4 | 1.127459924 |
| PRICKLE1 | -1.138599925 |  | SERPINB2 | 1.120148393 |
| ZNF462 | -1.138016851 |  | SPOCD1 | 1.117337638 |
| A2M | -1.137117942 |  | CD163 | 1.116683481 |
| ABLIM1 | -1.13580804 |  | C15orf52 | 1.116607243 |
| SLFN5 | -1.135409944 |  | BNIP3 | 1.115983663 |
| SORCS2 | -1.134137698 |  | LOC100129455 | 1.115648866 |
| TNFRSF19 | -1.133214771 |  | GAS6-AS1 | 1.114778316 |
| MMP7 | -1.132569001 |  | LOC100288152 | 1.114473577 |
| IFT80 | -1.132478965 |  | HLA-F | 1.114380214 |
| DCXR | -1.127987964 |  | TNFSF13 | 1.113483225 |
| UBE2R2 | -1.126589066 |  | HHIP-AS1 | 1.113201778 |
| VKORC1L1 | -1.125189649 |  | ZDHHC18 | 1.111126859 |
| TOR1AIP2 | -1.124723635 |  | SLC46A1 | 1.111056958 |
| SPOP | -1.123225123 |  | STMN3 | 1.109939877 |
| LINC00341 | -1.122853515 |  | TCEA3 | 1.107587396 |
| LARP7 | -1.120904688 |  | NOTCH2 | 1.107531439 |
| APOL4 | -1.119800483 |  | AXL | 1.106532022 |
| COL4A6 | -1.119664512 |  | FSTL3 | 1.106281755 |
| TAF5L | -1.118479842 |  | BTN3A1 | 1.105774822 |
| ZMYND11 | -1.118356468 |  | NEK7 | 1.105495379 |
| IQCH-AS1 | -1.117747145 |  | LARP1B | 1.10524791 |
| BMPER | -1.117312451 |  | EID2B | 1.104609847 |
| NR4A3 | -1.115722188 |  | ACYP1 | 1.104340464 |
| PRRC2B | -1.115033326 |  | KIAA1467 | 1.103998629 |
| GPBP1 | -1.111981329 |  | TRMT2B | 1.103836628 |
| PRR11 | -1.111704767 |  | EHD3 | 1.10203918 |
| RBP4 | -1.110722146 |  | GNL1 | 1.101840702 |
| TRIB2 | -1.110540671 |  | DNALI1 | 1.097916704 |
| LBP | -1.110204228 |  | JDP2 | 1.09682431 |
| HP | -1.109517145 |  | FLJ37453 | 1.093228999 |
| RARA | -1.107731288 |  | C4orf47 | 1.092267786 |
| IL1R1 | -1.106539453 |  | RASL11A | 1.090909328 |
| ANKH | -1.103394466 |  | KCNJ2 | 1.08933978 |
| ZHX1 | -1.102452218 |  | GALC | 1.087843562 |
| TENM2 | -1.102141512 |  | CLCN5 | 1.087447218 |
| AFAP1L1 | -1.101706976 |  | PEG10 | 1.086551094 |
| STK38L | -1.100456992 |  | CPXM2 | 1.085240473 |
| POLD2 | -1.099386543 |  | EVA1A | 1.084829965 |
| TNFAIP6 | -1.09922578 |  | LINC00888 | 1.083664981 |
| GHR | -1.098556467 |  | LSM11 | 1.083273808 |
| CCRL1 | -1.098428234 |  | GPR56 | 1.082469911 |
| CPED1 | -1.097581419 |  | YOD1 | 1.082407595 |
| LOC153577 | -1.095787849 |  | GM2A | 1.082159755 |
| SSPN | -1.094901535 |  | MRS2 | 1.078500203 |
| YPEL2 | -1.094461834 |  | KIAA1147 | 1.077237962 |
| TANK | -1.092976366 |  | IMMP1L | 1.076453226 |
| MEX3B | -1.092554398 |  | ZNF385B | 1.075872288 |
| TBL1XR1 | -1.091112191 |  | TM2D1 | 1.073776177 |
| TBL3 | -1.089588699 |  | GLIPR1 | 1.073706298 |
| MDFIC | -1.088368751 |  | LOC100507303 | 1.073052828 |
| ZNF106 | -1.087205995 |  | VMO1 | 1.072343821 |
| EEA1 | -1.087024881 |  | MEF2BNB | 1.070355047 |
| CCND2 | -1.086668803 |  | TESK2 | 1.070092268 |
| NAA15 | -1.084617935 |  | PIGB | 1.068888945 |
| TSFM | -1.082399057 |  | ATPIF1 | 1.067008688 |
| USP15 | -1.082163696 |  | VSIG10L | 1.066668686 |
| DDA1 | -1.081921107 |  | TAPBP | 1.065999251 |
| YME1L1 | -1.081832716 |  | GAL3ST4 | 1.063977619 |
| AKIRIN1 | -1.080737285 |  | ARMCX4 | 1.063465107 |
| PPP2R1B | -1.080483606 |  | PELI2 | 1.061730139 |
| PTN | -1.079094562 |  | GAB3 | 1.061228302 |
| SLC2A12 | -1.078955417 |  | AMN1 | 1.061064503 |
| CPM | -1.078915492 |  | MGLL | 1.060183609 |
| ZNF326 | -1.075621089 |  | TLR3 | 1.060071105 |
| PTMA | -1.07441435 |  | HLA-B | 1.060011945 |
| SPRY1 | -1.071830837 |  | MIR3682 | 1.056983066 |
| PPM1L | -1.071717953 |  | HPSE | 1.056017216 |
| SPCS3 | -1.070950338 |  | CXorf57 | 1.055800465 |
| ARHGAP42 | -1.070752399 |  | CYP26B1 | 1.05355542 |
| NASP | -1.069322541 |  | CITED2 | 1.052277101 |
| PALM2 | -1.067524075 |  | FLJ39739 | 1.052086676 |
| SGMS2 | -1.067380558 |  | ETNK1 | 1.051148245 |
| GCDH | -1.067124182 |  | PSKH1 | 1.050744585 |
| AQP3 | -1.066501472 |  | ZDHHC13 | 1.050120705 |
| ITSN1 | -1.066154159 |  | BTN2A2 | 1.048912015 |
| TM7SF2 | -1.065208374 |  | ABLIM3 | 1.047627307 |
| LEPR | -1.064407589 |  | GALNT5 | 1.046235006 |
| ITFG1 | -1.062806457 |  | PLEKHB2 | 1.04484533 |
| LOC100506748 | -1.062549073 |  | CPT1C | 1.044664195 |
| ZAK | -1.061115124 |  | KRT7 | 1.043954391 |
| GNG12 | -1.06045665 |  | SHB | 1.043531903 |
| KCTD1 | -1.060118312 |  | LOC338620 | 1.042529042 |
| MXI1 | -1.0596019 |  | GCNT3 | 1.041361355 |
| TMEM123 | -1.058551818 |  | PODXL | 1.04006009 |
| DLAT | -1.058147395 |  | ITGA5 | 1.040029392 |
| NIPBL | -1.056635069 |  | AFF3 | 1.038940897 |
| CUTC | -1.055575267 |  | C10orf54 | 1.038094752 |
| TRAF3 | -1.055253438 |  | SYNJ2 | 1.037730602 |
| TAF4B | -1.054822681 |  | TDRD7 | 1.037299671 |
| ABHD5 | -1.054146704 |  | DERL2 | 1.037131378 |
| CHURC1 | -1.049077059 |  | SNHG10 | 1.036895724 |
| SERPINA3 | -1.049072367 |  | DKK3 | 1.036801007 |
| ZNF880 | -1.047846553 |  | LINC00938 | 1.036792724 |
| BOC | -1.046902976 |  | MMP1 | 1.035581622 |
| DACT1 | -1.046552588 |  | ST7-AS1 | 1.035459822 |
| F2R | -1.045625015 |  | RENBP | 1.035293267 |
| GEM | -1.04560947 |  | PIFO | 1.03404111 |
| NR2F1 | -1.044004704 |  | LOC100507634 | 1.033210254 |
| GPBAR1 | -1.043444691 |  | NIPAL3 | 1.03285833 |
| ADAMTS9 | -1.043439999 |  | SLC35D1 | 1.032732132 |
| RAB1A | -1.042630706 |  | COL4A4 | 1.03229087 |
| MTMR4 | -1.040127134 |  | LINC00968 | 1.030978672 |
| HINT3 | -1.038617754 |  | BCL2L12 | 1.027161745 |
| LAMP2 | -1.038366646 |  | COL6A6 | 1.026261273 |
| MTIF2 | -1.037043836 |  | PLD1 | 1.025365811 |
| FRMD3 | -1.03636974 |  | RCAN2 | 1.021599688 |
| ETV5 | -1.034464035 |  | SPATA2 | 1.020101204 |
| SNX2 | -1.034250728 |  | NDFIP2 | 1.019828805 |
| ABCC9 | -1.030666502 |  | CD2BP2 | 1.019078488 |
| PSPH | -1.029949952 |  | SLC16A3 | 1.01852353 |
| MFSD12 | -1.029698866 |  | LOC100507637 | 1.018319534 |
| PDGFRL | -1.029347898 |  | TRIM29 | 1.01793657 |
| FKBP2 | -1.028149421 |  | QSOX1 | 1.017934364 |
| FKTN | -1.027391373 |  | C10orf85 | 1.01753247 |
| FZD8 | -1.027173972 |  | SLC16A4 | 1.017524082 |
| GALNT1 | -1.026758986 |  | WHSC1 | 1.017327477 |
| HOXB7 | -1.02631888 |  | TRIOBP | 1.016745129 |
| SNX9 | -1.025429363 |  | SLC25A51 | 1.016168994 |
| NUDT16 | -1.024988266 |  | LMBR1 | 1.016048799 |
| FABP4 | -1.024812212 |  | PCAT6 | 1.014068752 |
| SLC27A3 | -1.024039884 |  | BCL2A1 | 1.013764975 |
| G0S2 | -1.023578555 |  | UQCC | 1.011036584 |
| SERPINI1 | -1.021679973 |  | COL9A2 | 1.01076009 |
| ZCCHC9 | -1.021023742 |  | PHLPP2 | 1.009726511 |
| CEP57L1 | -1.020155169 |  | CLSTN2 | 1.009045566 |
| MITF | -1.020122451 |  | SLC2A3 | 1.008583912 |
| TMEM56 | -1.019547374 |  | SLC36A1 | 1.00829302 |
| KDM1B | -1.016085713 |  | FUCA1 | 1.008023976 |
| FBXW7 | -1.015266542 |  | VAMP5 | 1.007974211 |
| MYO10 | -1.013809898 |  | SORBS2 | 1.007823346 |
| MCAM | -1.01350712 |  | OLR1 | 1.007631311 |
| FKBP1A | -1.013142691 |  | SETD5-AS1 | 1.007211566 |
| PPAP2B | -1.011874135 |  | DOK3 | 1.00471524 |
| ADIPOR2 | -1.010198286 |  | HCLS1 | 1.002753253 |
| SPTBN1 | -1.008128723 |  | KRTAP1-3 | 1.001881644 |
| NEO1 | -1.007979402 |  | RPL13 | 1.000917479 |
| CMTM4 | -1.007903175 |  | MARCH2 | 1.000814722 |
| SETMAR | -1.007399818 |  | BST1 | 0.999573324 |
| PTH1R | -1.006923435 |  | KCNK1 | 0.999399356 |
| VANGL1 | -1.006797395 |  | MLPH | 0.99794521 |
| WWC1 | -1.006611867 |  | MRPL35 | 0.997037914 |
| RBMXL1 | -1.00548972 |  | LOC728819 | 0.995655685 |
| GRB14 | -1.004872496 |  | PARP14 | 0.99560554 |
| NRDE2 | -1.004481776 |  | DIDO1 | 0.995208622 |
| CISH | -1.004460245 |  | NIPSNAP3A | 0.995172118 |
| ECM2 | -1.003039724 |  | APOLD1 | 0.994868224 |
| LRRC8B | -1.002804434 |  | PLP2 | 0.99399359 |
| ITGB5 | -1.00160998 |  | GLA | 0.993856418 |
| RB1CC1 | -1.000198152 |  | UXS1 | 0.993810125 |
| MYO1B | -0.999977458 |  | SLC35E4 | 0.993137667 |
| RBM6 | -0.99909077 |  | PCTP | 0.992512978 |
| FAM155A | -0.998607176 |  | CAMK4 | 0.992509092 |
| MBTD1 | -0.997045779 |  | C5orf56 | 0.992054801 |
| RDX | -0.996049519 |  | C1orf85 | 0.990379569 |
| KIAA0232 | -0.996029383 |  | MYL12A | 0.989939371 |
| NUCKS1 | -0.994996783 |  | PSG3 | 0.989914455 |
| ZNF25 | -0.99470427 |  | HCG4 | 0.989603013 |
| CLUAP1 | -0.994094746 |  | FLJ10038 | 0.989017911 |
| ING3 | -0.994006447 |  | ABCG1 | 0.988512251 |
| FAM149A | -0.993532228 |  | PSG9 | 0.988373629 |
| MACROD1 | -0.993276375 |  | EML6 | 0.98789928 |
| CHI3L1 | -0.991398722 |  | FUT4 | 0.986235783 |
| C7 | -0.98984233 |  | FGL2 | 0.9841865 |
| C7orf63 | -0.989052919 |  | TFPI | 0.980109868 |
| FOXP2 | -0.988788564 |  | KPNA1 | 0.979806145 |
| CLMN | -0.988105975 |  | SERPINB9 | 0.979244269 |
| FCHSD2 | -0.985473546 |  | ZC3HAV1 | 0.978007689 |
| DYNC1I2 | -0.983397949 |  | GSTM5 | 0.976874527 |
| KDM5B | -0.983374726 |  | LINC00476 | 0.976549225 |
| ARMC1 | -0.982235132 |  | FLVCR2 | 0.9763011 |
| KCNMA1 | -0.981505338 |  | DTNA | 0.976158411 |
| LRRC58 | -0.981052634 |  | STK40 | 0.97256885 |
| C6orf62 | -0.978605374 |  | IFIT1 | 0.972543597 |
| ALDH1B1 | -0.976993351 |  | RCAN3 | 0.972397089 |
| TFDP2 | -0.976378518 |  | ZNF571-AS1 | 0.971685145 |
| HSD11B1 | -0.976319779 |  | KPNA4 | 0.970779119 |
| CDADC1 | -0.974658647 |  | IRF1 | 0.970671061 |
| RHOQ | -0.974612012 |  | PLOD1 | 0.9703407 |
| SUSD5 | -0.974442779 |  | C18orf56 | 0.97016398 |
| CUL4B | -0.973885868 |  | LOC100505812 | 0.969859634 |
| CLCA2 | -0.972560767 |  | ARSG | 0.966836721 |
| CIDEC | -0.971429976 |  | H2BFS | 0.966629917 |
| HOXB6 | -0.969207325 |  | FOLR3 | 0.9654819 |
| TMEM68 | -0.969003421 |  | PAIP1 | 0.965069586 |
| HMG20B | -0.967175325 |  | THAP10 | 0.964272116 |
| FAM210B | -0.966211752 |  | BCL2L1 | 0.96356335 |
| IVNS1ABP | -0.965160246 |  | KCNS3 | 0.962778085 |
| LRRC2 | -0.965003281 |  | HNRNPU-AS1 | 0.962748042 |
| NT5DC1 | -0.964979914 |  | PSMD11 | 0.962354455 |
| EYA2 | -0.964590102 |  | ACSS3 | 0.961712119 |
| RGCC | -0.964068227 |  | KIF1C | 0.961634644 |
| YAF2 | -0.963601179 |  | CHL1 | 0.96128539 |
| SLC2A6 | -0.963461113 |  | DDHD2 | 0.961223347 |
| STEAP2 | -0.963215327 |  | RAB11FIP3 | 0.961174538 |
| MEDAG | -0.963161592 |  | JMJD1C-AS1 | 0.960907819 |
| AGMAT | -0.962484796 |  | TSPYL4 | 0.959520331 |
| COL18A1 | -0.962353564 |  | TMEM106C | 0.957830938 |
| RAB11FIP2 | -0.962062751 |  | THAP2 | 0.957186035 |
| AOC2 | -0.960783889 |  | AP1G2 | 0.955954769 |
| ITGBL1 | -0.959774276 |  | NRIP3 | 0.955862005 |
| TCF4 | -0.959162042 |  | TRIM14 | 0.955805744 |
| ELK3 | -0.958035568 |  | PLEKHG4 | 0.955743387 |
| HIP1 | -0.957737087 |  | TMEM170A | 0.955551975 |
| IGF2BP3 | -0.957499564 |  | SUCO | 0.952538089 |
| TWISTNB | -0.957357002 |  | JMJD6 | 0.951986604 |
| ST6GAL1 | -0.956958911 |  | ZNF557 | 0.95117763 |
| TMEM106B | -0.955557296 |  | EIF2AK1 | 0.950773337 |
| HSPA14 | -0.955311289 |  | LAMB2 | 0.95076792 |
| TRAPPC10 | -0.954951613 |  | A2M-AS1 | 0.949849563 |
| CAV2 | -0.954771343 |  | TIGD7 | 0.947716831 |
| GOLIM4 | -0.954106089 |  | MYCL1 | 0.947147614 |
| CC2D2A | -0.953686842 |  | GINS1 | 0.947081548 |
| ELOVL3 | -0.951965647 |  | PPP1R26 | 0.946081979 |
| SSBP2 | -0.951832217 |  | ITM2C | 0.945706736 |
| ARHGAP6 | -0.950901281 |  | OAS2 | 0.945334028 |
| SVIL | -0.949772491 |  | PSG6 | 0.944643219 |
| PDXK | -0.946706367 |  | AK2 | 0.941834118 |
| GIN1 | -0.946540262 |  | RNF41 | 0.941808563 |
| COL13A1 | -0.946524978 |  | LOC100134937 | 0.941757022 |
| ATP2B1 | -0.946427149 |  | ACP5 | 0.941755797 |
| TTL | -0.945818223 |  | FIBIN | 0.941128176 |
| MMACHC | -0.944998193 |  | TFPT | 0.93924926 |
| NUDT5 | -0.944947882 |  | KIAA0319L | 0.939111462 |
| EMC4 | -0.944945445 |  | GDE1 | 0.935853974 |
| KLF13 | -0.94430043 |  | SERPINB7 | 0.935818832 |
| CNTLN | -0.943959834 |  | NIPA1 | 0.935796658 |
| DAP3 | -0.942946427 |  | GSTM3 | 0.934975865 |
| HAS2 | -0.942882901 |  | DAPK1 | 0.934450139 |
| RAB30 | -0.941568979 |  | ESR1 | 0.934031427 |
| PDGFRB | -0.941075953 |  | ZNF804A | 0.933165606 |
| TUBB2B | -0.940009506 |  | ARRDC4 | 0.933108012 |
| RDH5 | -0.938315124 |  | TMED9 | 0.933029191 |
| ASS1 | -0.938149755 |  | PNLIPRP3 | 0.932271086 |
| WDR5 | -0.937844089 |  | BCAS4 | 0.931063245 |
| HMCN1 | -0.937454494 |  | PENK | 0.930493502 |
| AR | -0.937210605 |  | DOK4 | 0.929551952 |
| STRBP | -0.936292704 |  | ST3GAL6 | 0.929516913 |
| ITPR2 | -0.935977125 |  | UPP1 | 0.92942038 |
| PLEKHF1 | -0.934532039 |  | TAPBPL | 0.929211278 |
| MAP4K5 | -0.932626907 |  | AJUBA | 0.928473824 |
| FAM45A | -0.932623114 |  | ABHD6 | 0.928240078 |
| GAREM | -0.931348542 |  | AP1S2 | 0.926123511 |
| DCLK1 | -0.930983971 |  | C8orf58 | 0.926009206 |
| TMEM154 | -0.92879028 |  | TTC17 | 0.925422687 |
| USP46 | -0.928610003 |  | SLC16A5 | 0.924386793 |
| ACSL1 | -0.928419684 |  | CKAP4 | 0.923659021 |
| IRAK1BP1 | -0.92637819 |  | EXT1 | 0.922944496 |
| MTHFD1 | -0.923738266 |  | GPR180 | 0.922637682 |
| APOOL | -0.923712765 |  | CYP3A7 | 0.922128897 |
| ARHGAP28 | -0.923293751 |  | PRL | 0.921039754 |
| RNF170 | -0.923131521 |  | NT5E | 0.918568479 |
| MCU | -0.922824501 |  | DONSON | 0.91829364 |
| BMPR2 | -0.922417075 |  | WDR55 | 0.916577325 |
| NCK1 | -0.922304988 |  | SLC22A4 | 0.916064429 |
| PHACTR2 | -0.922252057 |  | ZNF578 | 0.915433078 |
| HMGCR | -0.921836156 |  | OSR1 | 0.914385708 |
| CCDC41 | -0.921000842 |  | GSTO2 | 0.914086366 |
| MAU2 | -0.920874418 |  | LEPROT | 0.91331557 |
| UACA | -0.92060436 |  | PTPRN2 | 0.912317729 |
| MYO1D | -0.920169925 |  | ORAI3 | 0.911935883 |
| FDPS | -0.917455813 |  | PSEN2 | 0.911155274 |
| HNRNPA3 | -0.917046684 |  | NOL3 | 0.910922295 |
| GATA6 | -0.916002718 |  | ZNF674-AS1 | 0.910314968 |
| LOC100506965 | -0.91566733 |  | FNBP1 | 0.910229811 |
| BOP1 | -0.914626012 |  | SPRYD7 | 0.910042876 |
| ZNF608 | -0.913586207 |  | NFASC | 0.909760241 |
| LOC283788 | -0.913439953 |  | LINC00957 | 0.90938824 |
| RBFOX2 | -0.912902618 |  | TMEM206 | 0.909246044 |
| SERPING1 | -0.91163107 |  | MOB3B | 0.908777514 |
| GLYR1 | -0.911537753 |  | TTC28-AS1 | 0.908504738 |
| TMEM164 | -0.910130034 |  | ACYP2 | 0.90830153 |
| SMAD5 | -0.908445094 |  | DNASE2 | 0.907834898 |
| CTH | -0.908221229 |  | PSG1 | 0.907635255 |
| MVD | -0.9079229 |  | PRORSD1P | 0.907530869 |
| ANO6 | -0.907305395 |  | C7orf73 | 0.906904983 |
| BCAP29 | -0.906843189 |  | KCND2 | 0.906579246 |
| EVA1C | -0.906665871 |  | PRSS12 | 0.906290432 |
| CYLD | -0.906319758 |  | DNAJB4 | 0.905566064 |
| FAM83H | -0.906299922 |  | HSD17B11 | 0.905493147 |
| MKRN1 | -0.90582364 |  | SMS | 0.905481553 |
| ABCC2 | -0.90571746 |  | LOC100996592 | 0.904426884 |
| PDZRN3 | -0.905223738 |  | SEPW1 | 0.904397482 |
| IRX3 | -0.903746619 |  | EDIL3 | 0.904088444 |
| METTL21A | -0.903108111 |  | RNF130 | 0.903782821 |
| DCUN1D4 | -0.902856804 |  | DRP2 | 0.903496312 |
| INSR | -0.902481234 |  | RGS10 | 0.90323214 |
| CASP7 | -0.902313752 |  | AGPAT5 | 0.903090301 |
| CCSAP | -0.901328316 |  | ENTPD7 | 0.903058333 |
| KIAA1199 | -0.900885488 |  | IFITM1 | 0.901798451 |
| ZNF385D | -0.900666982 |  | TDG | 0.901221117 |
| IL18R1 | -0.899703112 |  | GNAS-AS1 | 0.90110084 |
| MKX | -0.899176912 |  | FLRT3 | 0.900772237 |
| ZBTB20 | -0.899019826 |  | LOC100127983 | 0.900002987 |
| BMPR1B | -0.898750845 |  | NUAK1 | 0.898962791 |
| C10orf10 | -0.897824929 |  | SLC39A9 | 0.898825526 |
| DENND4A | -0.897782608 |  | ERCC2 | 0.897666606 |
| LOC100506119 | -0.897777008 |  | PLCXD2 | 0.897371573 |
| ZSWIM7 | -0.897347026 |  | CCL26 | 0.896971137 |
| HADH | -0.896228129 |  | HAS1 | 0.896781934 |
| SYNE2 | -0.895934507 |  | DENND1B | 0.894934694 |
| PDE3A | -0.895744379 |  | SMIM10 | 0.894715936 |
| NIP7 | -0.895408099 |  | NCAM2 | 0.894224107 |
| MED11 | -0.894788208 |  | EPT1 | 0.893956098 |
| LOC100506365 | -0.893710345 |  | TNFRSF12A | 0.893755269 |
| UBE2H | -0.893483479 |  | FAM111A | 0.893688266 |
| NPR3 | -0.89337291 |  | HTR7P1 | 0.893286826 |
| PIGH | -0.892033856 |  | ATP6V0A1 | 0.891557585 |
| TCF12 | -0.890603484 |  | PARM1 | 0.890889833 |
| PRUNE2 | -0.889032189 |  | B3GAT3 | 0.890087692 |
| HTR2B | -0.88818531 |  | ABCC10 | 0.889617079 |
| AOC3 | -0.88782936 |  | HGF | 0.889601558 |
| UNC5B | -0.886941303 |  | HIST1H2AC | 0.889465945 |
| SCRN2 | -0.886584488 |  | LOXL4 | 0.889411737 |
| PDPN | -0.886499219 |  | PRMT2 | 0.888194557 |
| BTBD7 | -0.886091605 |  | C7orf50 | 0.888169998 |
| STRN | -0.88601905 |  | RGS4 | 0.888115297 |
| MEX3D | -0.885991056 |  | UBE2J1 | 0.887875358 |
| EIF2S1 | -0.885574433 |  | COTL1 | 0.887296849 |
| ASAP3 | -0.885436231 |  | GREB1 | 0.886549508 |
| ETV1 | -0.884564111 |  | SEC61A2 | 0.885086732 |
| ATP5L | -0.884163056 |  | CPNE2 | 0.884192687 |
| CENPN | -0.883908544 |  | PLA2G4C | 0.883779491 |
| ERAP1 | -0.88232209 |  | TMEM101 | 0.883445161 |
| NME5 | -0.882292067 |  | MFSD6 | 0.883160048 |
| LSS | -0.882218726 |  | DIRC2 | 0.883099787 |
| API5 | -0.881387954 |  | PSPC1 | 0.882876834 |
| RPL7L1 | -0.880345175 |  | CD9 | 0.882829697 |
| TSHR | -0.8797132 |  | TCEB2 | 0.882552421 |
| GPX3 | -0.879040726 |  | SLC22A5 | 0.882414023 |
| MAGI1 | -0.878888222 |  | ODC1 | 0.881375412 |
| CCDC50 | -0.878761868 |  | GPR19 | 0.88118193 |
| RTN4RL1 | -0.878041123 |  | C1orf54 | 0.880856512 |
| EIF3F | -0.876993017 |  | DCP1A | 0.880335315 |
| ERI2 | -0.876518411 |  | PARP12 | 0.879830735 |
| PLA2R1 | -0.875597133 |  | PLEK2 | 0.879746536 |
| UBALD2 | -0.87547631 |  | MAP3K8 | 0.879280804 |
| B3GNT5 | -0.874644045 |  | CFH | 0.878094183 |
| UBXN2A | -0.873611787 |  | LOC440028 | 0.877132511 |
| SSSCA1 | -0.873168588 |  | SVIP | 0.87605611 |
| GART | -0.872973216 |  | KIAA2013 | 0.87583698 |
| QSER1 | -0.872893443 |  | FAM49B | 0.87583007 |
| PREB | -0.872602843 |  | ATP6V0B | 0.875481062 |
| CCNYL1 | -0.872457404 |  | TMEM62 | 0.874951449 |
| TGIF2 | -0.872445777 |  | SLC2A11 | 0.874209123 |
| SYNM | -0.872152292 |  | PLBD1 | 0.873647808 |
| ADRA2A | -0.87139257 |  | ECM1 | 0.873458197 |
| CD36 | -0.870264858 |  | DNASE1L1 | 0.872834267 |
| ITGA1 | -0.868977684 |  | TMF1 | 0.87278124 |
| ZDHHC21 | -0.868944711 |  | ASPHD2 | 0.872444216 |
| AGGF1 | -0.868732469 |  | CDK15 | 0.872362428 |
| SNED1 | -0.868328645 |  | ZNF542 | 0.872266579 |
| KIF1B | -0.86779178 |  | MSRA | 0.872226254 |
| USP13 | -0.867651746 |  | LOC100505806 | 0.87197594 |
| RGL1 | -0.866628144 |  | RASSF8-AS1 | 0.871666315 |
| WNK4 | -0.865013846 |  | N4BP2L1 | 0.871404712 |
| EVI5 | -0.863573052 |  | LINC00525 | 0.871193412 |
| LIPA | -0.862517942 |  | CDH13 | 0.870497665 |
| ARAF | -0.862463125 |  | SGPP1 | 0.870378754 |
| C1R | -0.861862186 |  | WDR37 | 0.869968455 |
| CCDC66 | -0.861490604 |  | ZC3HAV1L | 0.869187245 |
| EIF3M | -0.860831152 |  | CERS2 | 0.868678465 |
| CLIC6 | -0.860226703 |  | CD97 | 0.867516981 |
| MBNL2 | -0.85985558 |  | SFXN1 | 0.866262916 |
| YPEL5 | -0.859419983 |  | CRABP2 | 0.86555234 |
| ACLY | -0.858657457 |  | HLA-C | 0.86468795 |
| XPOT | -0.858385357 |  | TCTE3 | 0.864453055 |
| MRPL16 | -0.857888202 |  | BHLHE40 | 0.862809128 |
| CINP | -0.857167996 |  | AGA | 0.860282949 |
| ZMYM2 | -0.856998529 |  | SESTD1 | 0.860227769 |
| ARHGAP5 | -0.856984604 |  | CTSZ | 0.85994774 |
| TBC1D5 | -0.856880702 |  | LOC284023 | 0.859862946 |
| GPHN | -0.854751728 |  | ERCC1 | 0.859158995 |
| PNPLA3 | -0.854215401 |  | MFSD11 | 0.858863446 |
| ACSF2 | -0.853821195 |  | TMEM179B | 0.855860452 |
| EOGT | -0.853716889 |  | PTPN18 | 0.85554264 |
| ZNF438 | -0.853443366 |  | SYNPO | 0.855449069 |
| PLA2G12A | -0.852865766 |  | GSTT1 | 0.855351687 |
| KIAA1644 | -0.852752881 |  | ZNF469 | 0.854816232 |
| CORO1C | -0.852589567 |  | FLOT2 | 0.854814663 |
| FUBP3 | -0.852528506 |  | MEI1 | 0.854742126 |
| PALLD | -0.852046642 |  | MESDC1 | 0.854654418 |
| MMD | -0.851883844 |  | DDO | 0.853470614 |
| DDX27 | -0.85176675 |  | TMTC1 | 0.853235992 |
| BMP2K | -0.850750521 |  | C12orf73 | 0.853141304 |
| SLC25A29 | -0.849718953 |  | CRIP1 | 0.852738199 |
| MEX3A | -0.848895946 |  | AIG1 | 0.852400078 |
| BHLHE41 | -0.84785903 |  | MED20 | 0.851861844 |
| SCARA5 | -0.847608814 |  | ANKRD11 | 0.851344979 |
| CCND1 | -0.847210625 |  | HLA-J | 0.851303415 |
| MEAF6 | -0.846963809 |  | LOC100505549 | 0.850644773 |
| MINA | -0.846385311 |  | STPG1 | 0.850530539 |
| KIAA1430 | -0.846073212 |  | MVB12B | 0.849653425 |
| TNPO3 | -0.846028409 |  | FUBP1 | 0.849499005 |
| TMTC4 | -0.84594781 |  | APCDD1L-AS1 | 0.849053143 |
| APCDD1 | -0.844463731 |  | PPP1R13L | 0.848737941 |
| STIP1 | -0.84346843 |  | GRTP1 | 0.847921068 |
| SIKE1 | -0.84182961 |  | MCOLN1 | 0.847897154 |
| THEM4 | -0.841296752 |  | FBXW2 | 0.847769557 |
| SUV420H1 | -0.840260568 |  | PPP2R2B | 0.847497906 |
| NEFL | -0.838632169 |  | TLR4 | 0.847148928 |
| GUCY1A3 | -0.838572824 |  | FKRP | 0.846655746 |
| EIF2AK4 | -0.838028858 |  | RASSF1 | 0.846236382 |
| ANGPT1 | -0.837285398 |  | LOC153682 | 0.845803357 |
| JAK2 | -0.836988318 |  | CDH4 | 0.845187073 |
| RAB14 | -0.836614167 |  | MINOS1 | 0.844969557 |
| COL15A1 | -0.836589073 |  | GAREML | 0.844182948 |
| EBAG9 | -0.836545846 |  | AHR | 0.843733639 |
| CGREF1 | -0.835746748 |  | HDX | 0.843603123 |
| DHX9 | -0.835339907 |  | PGM5-AS1 | 0.843114465 |
| LRP8 | -0.834585741 |  | PEAR1 | 0.842757714 |
| ANKRD44 | -0.834433943 |  | SMYD2 | 0.842372622 |
| TNFAIP8 | -0.833655878 |  | GNAS | 0.841566086 |
| MRAS | -0.8333749 |  | MARCH4 | 0.84138168 |
| SELRC1 | -0.833007468 |  | TAPT1 | 0.840503757 |
| ABCA6 | -0.832069235 |  | BVES | 0.840439096 |
| YPEL1 | -0.831216664 |  | PSG7 | 0.839727354 |
| STAT5A | -0.830927146 |  | GPRC5A | 0.839300236 |
| KANK2 | -0.83005489 |  | NFKBIE | 0.838915756 |
| YWHAE | -0.829556031 |  | TPK1 | 0.83870431 |
| KCNE3 | -0.829185478 |  | LMAN2L | 0.837909445 |
| CEP19 | -0.829114653 |  | KLHL3 | 0.837859306 |
| MYC | -0.828849295 |  | XYLT2 | 0.837708505 |
| TNRC6B | -0.828149495 |  | PFKP | 0.837544401 |
| RNF19A | -0.827928613 |  | SGK494 | 0.837210078 |
| ADAM32 | -0.82750418 |  | DKFZP434I0714 | 0.836471557 |
| PPP1R15B | -0.826651924 |  | F2RL1 | 0.835665129 |
| TSC22D1 | -0.826555532 |  | MEG3 | 0.835540582 |
| ZKSCAN1 | -0.826226654 |  | MGC21881 | 0.835481938 |
| TRIM2 | -0.825793332 |  | MAF | 0.835412523 |
| ZNF655 | -0.824640507 |  | TPP1 | 0.834483334 |
| FAM126A | -0.824355221 |  | EZR | 0.833684917 |
| GNL3 | -0.824196708 |  | MLLT10 | 0.833644112 |
| PDGFA | -0.824071747 |  | TMED3 | 0.833201584 |
| SETBP1 | -0.823699267 |  | POPDC3 | 0.833072382 |
| FAN1 | -0.823542305 |  | NXPE3 | 0.832886282 |
| LIFR | -0.821786672 |  | PRTFDC1 | 0.832759389 |
| LSM12 | -0.82157838 |  | LINC00883 | 0.832161973 |
| NNAT | -0.821054762 |  | TMED5 | 0.831807324 |
| GPRC5B | -0.820984952 |  | ADTRP | 0.831354492 |
| ERRFI1 | -0.820732239 |  | HIST1H2BK | 0.831292849 |
| HSD17B12 | -0.820419114 |  | RSPH3 | 0.831075153 |
| TGM2 | -0.819290793 |  | MYLK4 | 0.829827194 |
| AOX1 | -0.818816259 |  | MAB21L1 | 0.829294084 |
| RBM18 | -0.818748343 |  | SLC9A8 | 0.829163678 |
| CKB | -0.81864477 |  | CYB5R1 | 0.828008043 |
| NDUFV3 | -0.818118836 |  | LRCH2 | 0.82782607 |
| PLEKHH2 | -0.817598977 |  | GXYLT1 | 0.827696573 |
| PHF3 | -0.817206211 |  | RTCA | 0.827472717 |
| LOC100506795 | -0.816664283 |  | COQ10A | 0.827467979 |
| LYRM5 | -0.816403714 |  | LOC642361 | 0.826777834 |
| IL11RA | -0.815707527 |  | MAPK13 | 0.826755782 |
| DTWD1 | -0.815513223 |  | MAGED2 | 0.826684595 |
| FAM120AOS | -0.814911586 |  | TMEM184B | 0.826481354 |
| LSM4 | -0.814617611 |  | HSD11B1L | 0.826107437 |
| RGS3 | -0.81444875 |  | FAM134C | 0.825930705 |
| STXBP4 | -0.814446765 |  | AKR1B1 | 0.825807522 |
| G3BP2 | -0.81423455 |  | LOC646903 | 0.8250058 |
| SBNO1 | -0.813690233 |  | TRAPPC2 | 0.823079476 |
| MID1 | -0.812948147 |  | LRRC1 | 0.82273078 |
| MCM7 | -0.812127402 |  | ABHD12 | 0.822176506 |
| LYRM1 | -0.812054264 |  | AVL9 | 0.82209514 |
| PRIM1 | -0.81204012 |  | AZI2 | 0.821329191 |
| NAGK | -0.811617266 |  | AQP11 | 0.821078966 |
| FAM208B | -0.811508875 |  | ZXDC | 0.821037888 |
| ATP9A | -0.811007363 |  | LAMB3 | 0.820740646 |
| SLC35A3 | -0.810816509 |  | DPH3 | 0.820679538 |
| EIF4G3 | -0.810806289 |  | UNKL | 0.820604982 |
| ARHGAP35 | -0.810672318 |  | IGSF3 | 0.820595402 |
| XG | -0.810543097 |  | HOMER1 | 0.820441281 |
| NR1D2 | -0.810258927 |  | PRDM1 | 0.819014722 |
| OSBPL5 | -0.809780886 |  | CERS5 | 0.818465615 |
| LOX | -0.808827384 |  | ZP1 | 0.817784643 |
| RCN2 | -0.808144 |  | MORN4 | 0.817103308 |
| TCF3 | -0.807780991 |  | SLC24A6 | 0.816783263 |
| ALDH1L2 | -0.807344186 |  | CUL5 | 0.816151998 |
| SRSF11 | -0.806862085 |  | TBC1D22B | 0.816088043 |
| SOCS2 | -0.803744769 |  | NLK | 0.816018257 |
| HDAC5 | -0.802924461 |  | CEP97 | 0.815445271 |
| ADAM33 | -0.80256905 |  | FMN2 | 0.815124134 |
| NRXN3 | -0.80237736 |  | CRIPAK | 0.814549759 |
| ARIH1 | -0.80218355 |  | TRAM1L1 | 0.814005371 |
| AP4E1 | -0.802044818 |  | SMAD3 | 0.813438608 |
| NRP2 | -0.80191301 |  | MTFR2 | 0.812081039 |
| SPATA22 | -0.801549956 |  | CASP1 | 0.811156373 |
| PHC3 | -0.801208394 |  | SERPINB1 | 0.810891523 |
| LOC100652772 | -0.801124334 |  | RPS6KA3 | 0.810156474 |
| SLC1A3 | -0.80032002 |  | SNTB1 | 0.808504188 |
| HS2ST1 | -0.799937012 |  | EME2 | 0.808197386 |
| TUBD1 | -0.799465057 |  | RNF121 | 0.808187065 |
| CPT2 | -0.798576502 |  | SNX5 | 0.807935623 |
| PIK3IP1 | -0.797222492 |  | ATP7B | 0.807741523 |
| SEMA6D | -0.796995734 |  | SHISA9 | 0.806896529 |
| MPC2 | -0.796896782 |  | SKA2 | 0.806846955 |
| SLC38A4 | -0.796661785 |  | FUCA2 | 0.806385742 |
| BCKDHB | -0.796225084 |  | LOC100507018 | 0.806310099 |
| IFT57 | -0.795992604 |  | LOC100287177 | 0.805985113 |
| CDKN1B | -0.794461271 |  | FBLN7 | 0.803980829 |
| LRRN3 | -0.794380404 |  | AKT2 | 0.803630068 |
| RORA | -0.794000389 |  | LY75 | 0.80315155 |
| TXNRD1 | -0.793859325 |  | THUMPD2 | 0.802854858 |
| DDX55 | -0.792979292 |  | DUSP3 | 0.802376662 |
| FHL1 | -0.792963302 |  | PKM | 0.801600763 |
| ASB8 | -0.7924417 |  | LOC101060091 | 0.801304133 |
| RFX2 | -0.792323304 |  | XPNPEP3 | 0.801188841 |
| PPARGC1A | -0.790971406 |  | TBRG1 | 0.800768301 |
| ADARB1 | -0.790971183 |  | MBOAT2 | 0.800688037 |
| FAM120A | -0.79053246 |  | PPP2R2D | 0.800503157 |
| KIAA0922 | -0.789969974 |  | GUCY1A2 | 0.799438559 |
| PLAC9 | -0.789879135 |  | TRO | 0.798957086 |
| UNG | -0.789496071 |  | DTX3L | 0.798902684 |
| GSKIP | -0.789328784 |  | FBXL2 | 0.798662544 |
| TFDP1 | -0.788789737 |  | HIF1AN | 0.797625562 |
| VHL | -0.788780884 |  | E2F7 | 0.797405116 |
| RASSF4 | -0.788733093 |  | ARL6 | 0.797174906 |
| MAGI2-AS3 | -0.788624882 |  | RAB11FIP1 | 0.797085573 |
| TARBP1 | -0.787241781 |  | SNX24 | 0.797019075 |
| RPRD1A | -0.786649635 |  | TSPAN4 | 0.79692085 |
| LOC101060353 | -0.785205284 |  | SLC25A4 | 0.796715089 |
| ACADSB | -0.785153573 |  | GPR107 | 0.796073465 |
| RBM48 | -0.784726679 |  | HOXB-AS1 | 0.795705014 |
| UBTD2 | -0.784446718 |  | CXorf24 | 0.795506505 |
| ZNF271 | -0.784326306 |  | GIT2 | 0.795063965 |
| IDH1-AS1 | -0.783293075 |  | IFIH1 | 0.795041244 |
| LRRFIP1 | -0.783271389 |  | TMEM120B | 0.794449548 |
| SH3RF3 | -0.783250632 |  | NDNF | 0.793629663 |
| KCTD21 | -0.782448878 |  | SPATA18 | 0.79318484 |
| LCORL | -0.782394357 |  | FLVCR1-AS1 | 0.7929271 |
| ADH1C | -0.782248758 |  | OXSR1 | 0.792885521 |
| EHMT1 | -0.781380603 |  | PLS1 | 0.792801285 |
| HNRNPUL2 | -0.781336807 |  | MED28 | 0.792640688 |
| PLEKHA1 | -0.781165436 |  | KIAA1919 | 0.791248636 |
| WDR78 | -0.780739891 |  | GSG1 | 0.790657174 |
| JHDM1D | -0.780648999 |  | UBE2W | 0.790121534 |
| TCF7L2 | -0.780536829 |  | TRPC4 | 0.789855329 |
| IL17D | -0.779949007 |  | ANO3 | 0.78962949 |
| TMEM132B | -0.779939215 |  | TBC1D13 | 0.789052363 |
| RUNX1T1 | -0.779352862 |  | PDIA3 | 0.788962657 |
| MAML3 | -0.779323944 |  | SLC33A1 | 0.788902248 |
| RAP2A | -0.778357251 |  | COMMD7 | 0.788536604 |
| KREMEN1 | -0.778331276 |  | CHRDL1 | 0.788288412 |
| MAP1LC3B2 | -0.778125535 |  | SYTL4 | 0.78827642 |
| LUC7L2 | -0.777495256 |  | PCDH7 | 0.787629369 |
| TBC1D9 | -0.777465927 |  | ZNF677 | 0.787582127 |
| EZH1 | -0.776264331 |  | PSMD9 | 0.787001761 |
| SLIT3 | -0.776079937 |  | SIRT7 | 0.786898022 |
| TAF1B | -0.77550913 |  | ARSA | 0.78592707 |
| PTCD2 | -0.775458724 |  | CACNA1A | 0.785861199 |
| INSIG1 | -0.774880829 |  | NKX3-1 | 0.785451261 |
| FAM76B | -0.774377076 |  | CHPT1 | 0.785421744 |
| ARSD | -0.774271272 |  | PTCHD3P1 | 0.785299089 |
| PRDM2 | -0.773147287 |  | LOC400043 | 0.784878859 |
| ZNF516 | -0.772992055 |  | PTBP3 | 0.783883321 |
| UTP15 | -0.771951509 |  | TAF5 | 0.78333887 |
| CRYAB | -0.771148424 |  | PAOX | 0.783020479 |
| ABCF2 | -0.770542219 |  | PRCP | 0.781866295 |
| UBE2G2 | -0.768940608 |  | SMCO4 | 0.781563638 |
| PMP22 | -0.76710271 |  | LOC100132352 | 0.78152197 |
| AQPEP | -0.764451376 |  | FRZB | 0.781274142 |
| LOC100506325 | -0.764375909 |  | TOPORS-AS1 | 0.780934589 |
| HOXA7 | -0.763699404 |  | MGC12916 | 0.780781807 |
| PCDHB14 | -0.763328118 |  | ZNF267 | 0.780606809 |
| PPP1R3E | -0.763035929 |  | SUCNR1 | 0.780194365 |
| IQCK | -0.762988442 |  | SALL2 | 0.779978158 |
| GFOD2 | -0.762880625 |  | HCG26 | 0.779587707 |
| FAM107B | -0.761963262 |  | SLC11A2 | 0.779542321 |
| U2AF1 | -0.761433703 |  | NOVA1 | 0.779378726 |
| PBRM1 | -0.761112695 |  | DTX4 | 0.779318771 |
| ARHGEF3 | -0.760968404 |  | TMEM17 | 0.7791678 |
| NSDHL | -0.760324137 |  | LCLAT1 | 0.779061617 |
| OPLAH | -0.759917424 |  | ZNF302 | 0.778861601 |
| SYNCRIP | -0.759373055 |  | FOXL1 | 0.778746397 |
| BACH2 | -0.75820365 |  | C1orf115 | 0.778486805 |
| ITPR1 | -0.75785252 |  | GMFB | 0.778026048 |
| MAPK10 | -0.757096748 |  | C12orf49 | 0.777249293 |
| GSK3B | -0.756776347 |  | FLNB | 0.777080481 |
| MARCKS | -0.756480126 |  | MAP6D1 | 0.776621069 |
| FGD5-AS1 | -0.755965671 |  | NMNAT2 | 0.776347918 |
| CACNA1C | -0.755867655 |  | SALL1 | 0.776321862 |
| ERI1 | -0.755798377 |  | NLRC5 | 0.776208667 |
| PTPRD | -0.755710528 |  | LIMS2 | 0.776158126 |
| PKN2 | -0.755670223 |  | LIMK2 | 0.77601143 |
| WDR89 | -0.755322959 |  | LOC100506469 | 0.775910343 |
| CDH11 | -0.755061147 |  | TMEM37 | 0.775705073 |
| PDHA1 | -0.754982421 |  | SLC22A17 | 0.775501366 |
| PTPLB | -0.754319822 |  | PARP8 | 0.775391479 |
| SAMD8 | -0.75422432 |  | TMED4 | 0.774899341 |
| MMP9 | -0.75396374 |  | CNPY3 | 0.774654188 |
| LDLR | -0.753539826 |  | MTFP1 | 0.774071668 |
| CLIP1 | -0.753488459 |  | SYS1 | 0.773839578 |
| CDK12 | -0.752817485 |  | TM7SF3 | 0.773802037 |
| SGCD | -0.75268515 |  | IMPA1 | 0.773683861 |
| SGTB | -0.751807218 |  | RASSF3 | 0.77350082 |
| KIAA1217 | -0.751523854 |  | LOC654433 | 0.773440924 |
| RIMKLB | -0.750632024 |  | AGPAT1 | 0.773284347 |
| STK32A | -0.750492457 |  | ENTPD5 | 0.773141118 |
| MYCBP2 | -0.750363841 |  | DDX58 | 0.773088605 |
| ASMTL | -0.748987277 |  | ELOVL4 | 0.77304882 |
| EMP1 | -0.747957527 |  | NFE2L3 | 0.772806943 |
| TRIM24 | -0.747534766 |  | SMIM7 | 0.772746299 |
| SMAD9 | -0.74670903 |  | ASAH1 | 0.772472868 |
| PCDH18 | -0.746655217 |  | PAQR7 | 0.772446017 |
| SPHK1 | -0.746508686 |  | GOLT1B | 0.772231504 |
| DNAJC21 | -0.746415057 |  | ZNF518B | 0.772026889 |
| ATP8B2 | -0.746294595 |  | SMIM8 | 0.771706762 |
| STAM | -0.746283929 |  | AGFG1 | 0.771120377 |
| ENSA | -0.74608024 |  | ZBTB5 | 0.770851284 |
| JMJD4 | -0.74583222 |  | GANC | 0.770168378 |
| FBXO33 | -0.74564108 |  | PTPDC1 | 0.769170994 |
| CYGB | -0.745055991 |  | PCYOX1L | 0.769115506 |
| IL6ST | -0.74499756 |  | KHNYN | 0.768983131 |
| PGM2L1 | -0.744692871 |  | CXCL3 | 0.768966487 |
| E2F5 | -0.744124818 |  | TRPV2 | 0.767257765 |
| ADSS | -0.743726236 |  | HEXIM1 | 0.76695952 |
| TNFSF10 | -0.743061406 |  | SIK1 | 0.766517109 |
| LHFPL2 | -0.742513793 |  | RNASEH2C | 0.766290479 |
| FAM101B | -0.742452696 |  | LOC100996388 | 0.766193434 |
| CSDE1 | -0.742422978 |  | FBXL20 | 0.766182572 |
| FUNDC2 | -0.742360836 |  | ORAOV1 | 0.765968083 |
| RCAN1 | -0.742225314 |  | TMEM9 | 0.765492566 |
| C16orf74 | -0.741994524 |  | ADCY6 | 0.765459678 |
| LINC00467 | -0.741960123 |  | MAGEH1 | 0.765383702 |
| ROR2 | -0.741680258 |  | TNFRSF14 | 0.765224482 |
| ANGPTL2 | -0.74069502 |  | IFIT3 | 0.764841767 |
| GPD2 | -0.739761319 |  | PCBD1 | 0.764326151 |
| CCNG2 | -0.73925084 |  | LOC284513 | 0.764103953 |
| CXorf38 | -0.738409626 |  | C9orf85 | 0.76369655 |
| ZFYVE26 | -0.738379688 |  | AREL1 | 0.763546469 |
| TMEM194A | -0.738166369 |  | LOC152742 | 0.763340716 |
| RIOK2 | -0.738088477 |  | AGPAT4-IT1 | 0.763123996 |
| LRIG1 | -0.738062578 |  | B3GALT4 | 0.763099858 |
| WWP1 | -0.737103177 |  | COX17 | 0.761407906 |
| OSER1-AS1 | -0.736713636 |  | ARSB | 0.760869685 |
| ZFAND4 | -0.736596961 |  | ST7L | 0.76064455 |
| ECE1 | -0.736355783 |  | PIAS2 | 0.760204849 |
| GTF2H1 | -0.73520225 |  | CCL13 | 0.759827417 |
| ARMC9 | -0.735111354 |  | P4HA2 | 0.759742081 |
| BACE1 | -0.734480521 |  | LINC00312 | 0.759671394 |
| HEATR2 | -0.734365097 |  | HOXD10 | 0.759485374 |
| RAB7A | -0.733668235 |  | IPO9 | 0.759384262 |
| H2AFV | -0.732817568 |  | SQSTM1 | 0.758962333 |
| ANP32A | -0.732691193 |  | SKP1 | 0.758547946 |
| SYBU | -0.731680514 |  | FAS | 0.75775866 |
| KIAA1377 | -0.731588891 |  | DPY19L1 | 0.757674136 |
| ACOX1 | -0.731333013 |  | BTN2A1 | 0.757497506 |
| ARPC1A | -0.731122241 |  | TRAPPC13 | 0.75643742 |
| PSMA5 | -0.729556664 |  | PABPC5 | 0.755451087 |
| PRDM5 | -0.729454824 |  | LOC100129195 | 0.75525289 |
| ELF2 | -0.729377453 |  | WBSCR22 | 0.754700951 |
| TTC37 | -0.729156303 |  | ZFYVE27 | 0.754590348 |
| TRMT5 | -0.72914514 |  | LOC338799 | 0.754353769 |
| VPS37A | -0.728982827 |  | GPR137B | 0.754245998 |
| GLUL | -0.728887677 |  | ZEB1-AS1 | 0.753925907 |
| PPIG | -0.728422772 |  | ROBO1 | 0.753832031 |
| CNOT4 | -0.728338952 |  | ZNF33B | 0.753722493 |
| TOMM70A | -0.727927951 |  | AGO4 | 0.752400125 |
| HK2 | -0.727822099 |  | MFSD9 | 0.752298022 |
| PXK | -0.727460323 |  | CD58 | 0.75201662 |
| DAAM1 | -0.727368203 |  | MESDC2 | 0.751543083 |
| PDE8B | -0.727349094 |  | C12orf76 | 0.751526172 |
| CWC27 | -0.72656893 |  | PROSER2 | 0.751513448 |
| FAXDC2 | -0.726160746 |  | EGLN1 | 0.751432725 |
| BTBD11 | -0.725800581 |  | PSMD5-AS1 | 0.751252576 |
| MT1P3 | -0.725665065 |  | SP110 | 0.750440109 |
| BNC2 | -0.72563961 |  | NPEPL1 | 0.750222906 |
| ADCK3 | -0.724531428 |  | CMPK2 | 0.749942182 |
| CKS1B | -0.724350822 |  | PCBP1-AS1 | 0.749889747 |
| FAM172A | -0.724219857 |  | ARHGAP20 | 0.749676143 |
| TPD52L1 | -0.723111619 |  | HLA-DRA | 0.74867047 |
| CSNK1G1 | -0.723032808 |  | MTMR2 | 0.748368861 |
| PDLIM5 | -0.723026452 |  | STX6 | 0.748124609 |
| BBS1 | -0.722614715 |  | TCTN3 | 0.747973125 |
| MSX1 | -0.722435671 |  | DCBLD2 | 0.747908563 |
| DDX52 | -0.722397827 |  | FGD4 | 0.747795302 |
| MMAB | -0.72230115 |  | NAGA | 0.74752047 |
| MECP2 | -0.722265672 |  | CHST11 | 0.747440932 |
| FAM20C | -0.721818379 |  | PPAPDC2 | 0.7471645 |
| CTSC | -0.721797206 |  | MAGEF1 | 0.746425551 |
| ZADH2 | -0.721761031 |  | LUM | 0.746372546 |
| ALKBH2 | -0.720770231 |  | SFT2D3 | 0.745775198 |
| C4orf33 | -0.720647973 |  | SLC30A6 | 0.745238612 |
| ABCA1 | -0.720339385 |  | UBE2D3 | 0.745181033 |
| PECR | -0.720252982 |  | KDM5A | 0.74516899 |
| STAT5B | -0.720126395 |  | ABCA5 | 0.744933351 |
| ERG | -0.719625636 |  | YDJC | 0.744899624 |
| HNRNPA1 | -0.719554948 |  | DTX3 | 0.744860945 |
| PCDHB7 | -0.718551874 |  | FAM122B | 0.744244939 |
| MTMR10 | -0.717738055 |  | GID8 | 0.743790964 |
| KLF3 | -0.71723732 |  | FAM131B | 0.743775874 |
| RELL1 | -0.716299857 |  | HIST1H2BH | 0.743150483 |
| SOS1 | -0.716272043 |  | BRE-AS1 | 0.74252774 |
| COL16A1 | -0.715958014 |  | ZHX1-C8ORF76 | 0.742189816 |
| STS | -0.715945892 |  | ZFP3 | 0.740511969 |
| SWAP70 | -0.715081139 |  | SERINC2 | 0.740484125 |
| LRRFIP2 | -0.714935362 |  | WDHD1 | 0.740374997 |
| PAN2 | -0.714740247 |  | CLPTM1 | 0.739855649 |
| MCM6 | -0.714373502 |  | LOC154761 | 0.73980868 |
| TACC2 | -0.713978901 |  | TMEM14A | 0.739758662 |
| CCDC59 | -0.713868456 |  | POGLUT1 | 0.739521476 |
| C15orf57 | -0.713670817 |  | TM9SF1 | 0.73941249 |
| KAT6B | -0.713628025 |  | HLA-E | 0.739185725 |
| FAM65C | -0.713044879 |  | DKK1 | 0.738941903 |
| MN1 | -0.712933436 |  | HOXC11 | 0.738566579 |
| C5orf22 | -0.712906282 |  | C10orf12 | 0.738547255 |
| LAMA3 | -0.712717384 |  | TNIK | 0.738423168 |
| RAB15 | -0.712395469 |  | ZNF211 | 0.738053546 |
| NFIX | -0.712304688 |  | HOXB8 | 0.73804795 |
| MAGI3 | -0.711739984 |  | PON2 | 0.737995357 |
| BRAF | -0.711529822 |  | ZNF215 | 0.737945528 |
| ATRX | -0.711196459 |  | SYNRG | 0.736703335 |
| SEPT10 | -0.710974115 |  | NHP2L1 | 0.736369554 |
| IFNGR1 | -0.710108277 |  | SRD5A3 | 0.736291249 |
| BCL2L11 | -0.71008536 |  | ACP6 | 0.735966879 |
| CCDC169 | -0.709981405 |  | C19orf10 | 0.735696423 |
| KCNK3 | -0.709924278 |  | MAGT1 | 0.735490876 |
| MAPK9 | -0.708995365 |  | MECOM | 0.734785676 |
| EPB41 | -0.708754696 |  | APBA1 | 0.733846458 |
| PRRX2 | -0.707331316 |  | TPBG | 0.732907733 |
| RRP7A | -0.70659957 |  | MMP12 | 0.732491926 |
| PI4K2A | -0.706583615 |  | POLR1D | 0.732284437 |
| GPT2 | -0.706540842 |  | PAG1 | 0.731763129 |
| RC3H2 | -0.705972078 |  | ZNF204P | 0.731730516 |
| ING5 | -0.70592417 |  | SNX27 | 0.731290844 |
| SOBP | -0.705853208 |  | SLC8A1 | 0.7311799 |
| CMTM7 | -0.705631137 |  | LOC645513 | 0.730842377 |
| PSAT1 | -0.705169835 |  | SMPD1 | 0.730732458 |
| CCDC23 | -0.705101484 |  | LOC100505738 | 0.730025622 |
| EXOC5 | -0.704924154 |  | TUSC2 | 0.730000636 |
| GNAL | -0.704621011 |  | DBP | 0.729949193 |
| COL11A1 | -0.70455234 |  | REEP5 | 0.729808119 |
| MT1X | -0.704395729 |  | EMB | 0.729472359 |
| DFFA | -0.704004676 |  | SLC44A1 | 0.729295709 |
| ZBED3-AS1 | -0.703651331 |  | TMEM136 | 0.728760973 |
| FSTL1 | -0.703436008 |  | ZNF836 | 0.727053961 |
| WRN | -0.703398666 |  | ZNF850 | 0.726182663 |
| MAPKAP1 | -0.703333558 |  | PTGER2 | 0.726140717 |
| MSI2 | -0.703205055 |  | ICAM2 | 0.725984117 |
| FOS | -0.702015729 |  | BARD1 | 0.725722774 |
| NOLC1 | -0.701778706 |  | FAM162B | 0.725601009 |
| COL8A1 | -0.70135566 |  | FAM134A | 0.725327826 |
| NAV2 | -0.701281151 |  | MYADM | 0.725251913 |
| C6orf89 | -0.700926364 |  | MSRB2 | 0.725033574 |
| FZD4 | -0.700832926 |  | RHPN2 | 0.724746158 |
| WISP1 | -0.700721569 |  | FNIP1 | 0.724720041 |
| DDX5 | -0.700687733 |  | SLC26A11 | 0.724073214 |
| MAP9 | -0.700621353 |  | KAZALD1 | 0.723857416 |
| TROVE2 | -0.700596961 |  | SPNS1 | 0.723730794 |
| LPP-AS2 | -0.700576621 |  | MYOZ2 | 0.723252529 |
| RABL3 | -0.700280437 |  | DPAGT1 | 0.722987373 |
| LOC100129461 | -0.70000755 |  | CHPF2 | 0.722390944 |
| TCP11L2 | -0.699974182 |  | MMP16 | 0.722025689 |
| PIP4K2A | -0.699848753 |  | C7orf13 | 0.721710725 |
| GJD3 | -0.699735598 |  | CLN8 | 0.721694312 |
| PLEKHH1 | -0.699706633 |  | PTDSS1 | 0.721520283 |
| RFC5 | -0.699483168 |  | RBBP4 | 0.721371857 |
| FAM214B | -0.699391947 |  | SRPRB | 0.721325611 |
| KLHDC1 | -0.69921012 |  | KDM3A | 0.719869959 |
| YTHDF1 | -0.699004327 |  | CST3 | 0.719655093 |
| SERPINF1 | -0.698943156 |  | BROX | 0.719635102 |
| COA4 | -0.698569782 |  | EMILIN2 | 0.718857521 |
| GLUD1 | -0.698552995 |  | ZNF85 | 0.718682345 |
| KATNBL1 | -0.698439974 |  | TCIRG1 | 0.718315316 |
| METTL7A | -0.698026965 |  | EXD3 | 0.718268724 |
| PGM3 | -0.69777677 |  | SELT | 0.718260724 |
| SPRY4 | -0.697543923 |  | CC2D1B | 0.717819908 |
| TSC22D3 | -0.697392322 |  | HPCAL1 | 0.71764787 |
| PLXNA4 | -0.697325429 |  | ZNF226 | 0.717647489 |
| TRMT10A | -0.697282992 |  | SCN8A | 0.717132955 |
| SLC16A6 | -0.696508827 |  | FAM178A | 0.7170306 |
| PDE4DIP | -0.696376949 |  | BCOR | 0.716853083 |
| RNA45S5 | -0.696369994 |  | CSTA | 0.71666777 |
| EXOSC2 | -0.696349051 |  | LOC100505761 | 0.715984118 |
| WSB2 | -0.696230225 |  | ECE2 | 0.7154229 |
| RNASEL | -0.695552592 |  | THSD4 | 0.714855126 |
| CHTOP | -0.695165606 |  | DNAJC10 | 0.714636693 |
| ADH1B | -0.694910337 |  | MMP8 | 0.713899537 |
| KDM4B | -0.694695326 |  | KDELR2 | 0.713737905 |
| ZSCAN2 | -0.694633355 |  | LOC100507516 | 0.71366426 |
| PICALM | -0.694584108 |  | CAMSAP2 | 0.713521526 |
| ILF3 | -0.694529412 |  | RAVER2 | 0.713350956 |
| ATP8B4 | -0.694474341 |  | FAP | 0.713290472 |
| PDIA6 | -0.694221183 |  | DNAJC5 | 0.712959289 |
| KBTBD6 | -0.69379237 |  | ZMYM6 | 0.71295916 |
| FRMD4A | -0.69375042 |  | EXTL3 | 0.712808993 |
| PEX2 | -0.69324096 |  | IFITM10 | 0.712760408 |
| ABHD11 | -0.692992651 |  | SGCE | 0.712343524 |
| C5orf24 | -0.692671231 |  | NDUFA7 | 0.712324091 |
| NFX1 | -0.692601556 |  | GALNT2 | 0.712045892 |
| DUSP6 | -0.69245044 |  | RPS28 | 0.71186658 |
| MKRN2 | -0.692423645 |  | ELAVL1 | 0.711371531 |
| LOC155060 | -0.69227825 |  | EIF3J-AS1 | 0.710097543 |
| OBSL1 | -0.692267587 |  | RCE1 | 0.710086988 |
| QRSL1 | -0.691740653 |  | ZNF83 | 0.709661231 |
| CAPRIN1 | -0.691639832 |  | C5orf54 | 0.709499176 |
| FAM160B1 | -0.691122055 |  | SLC2A4RG | 0.708932687 |
| DBN1 | -0.691100306 |  | MAN2A2 | 0.708902321 |
| TIAM1 | -0.690424703 |  | NR1H3 | 0.708848781 |
| TMEM43 | -0.690232969 |  | P4HB | 0.708687262 |
| PCDH9 | -0.690191906 |  | MYL9 | 0.708403958 |
| SEPT11 | -0.689923147 |  | UFM1 | 0.708224359 |
| TRIM37 | -0.689839244 |  | TPCN1 | 0.70810323 |
| AKNA | -0.689795889 |  | NAGPA | 0.70803831 |
| MRPS25 | -0.689600048 |  | VWCE | 0.707509383 |
| TOP2A | -0.689014 |  | PGM2 | 0.70736872 |
| ASAP2 | -0.688818427 |  | PAK1 | 0.707295518 |
| CCBE1 | -0.688688967 |  | LRRC28 | 0.706643314 |
| LRRK2 | -0.686717606 |  | BATF3 | 0.706313634 |
| ARHGEF6 | -0.686462774 |  | HOXA1 | 0.706254408 |
| LDLRAD3 | -0.686399442 |  | HOXD8 | 0.705986435 |
| MTHFD2 | -0.686388487 |  | CYTH1 | 0.70579503 |
| CCDC91 | -0.685810692 |  | LOC100310756 | 0.705723117 |
| CASK | -0.685362292 |  | IKBIP | 0.70490853 |
| CAST | -0.685000174 |  | PPAPDC1B | 0.704236626 |
| CDC42BPA | -0.684874048 |  | LOC284926 | 0.704200088 |
| SERINC5 | -0.68347073 |  | CBLB | 0.704083598 |
| ARGLU1 | -0.682407312 |  | MYLIP | 0.703902522 |
| FABP3 | -0.681836189 |  | GDF15 | 0.703827898 |
| MED21 | -0.680667801 |  | GRIN2D | 0.703559372 |
| DPP9 | -0.680160744 |  | BCHE | 0.703411611 |
| HES1 | -0.680133524 |  | RGMB | 0.703143536 |
| MTF1 | -0.680089551 |  | CISD2 | 0.702941285 |
| RBPMS | -0.67998498 |  | TMEM33 | 0.702256465 |
| PTPN1 | -0.679848515 |  | KRBOX1 | 0.702245738 |
| DIAPH2 | -0.679533978 |  | NAP1L1 | 0.701741208 |
| EBF1 | -0.679272882 |  | SLC39A13 | 0.701634114 |
| ITGA4 | -0.679237892 |  | ATP8A1 | 0.701623089 |
| BRI3BP | -0.679115843 |  | CNTN1 | 0.701465579 |
| TMOD2 | -0.678993469 |  | NPTXR | 0.701220764 |
| WWTR1 | -0.678808579 |  | SERTAD1 | 0.70065657 |
| ASF1A | -0.677748595 |  | C1QTNF6 | 0.700502042 |
| FGD6 | -0.677727603 |  | FAM26F | 0.700144172 |
| NUP50 | -0.677677868 |  | PSEN1 | 0.700078192 |
| KCMF1 | -0.677527869 |  | FAM177A1 | 0.700046451 |
| DHCR7 | -0.677326053 |  | CYBA | 0.699772388 |
| ERVK3-1 | -0.676707615 |  | FAR2 | 0.699737981 |
| GID4 | -0.676521033 |  | CPT1A | 0.699516804 |
| CDO1 | -0.676212409 |  | FUT11 | 0.699287363 |
| CILP2 | -0.676204716 |  | LINC00520 | 0.69900118 |
| ZNF576 | -0.676039236 |  | ZFPM2 | 0.698942106 |
| TMEM119 | -0.675896647 |  | PCGF2 | 0.698898576 |
| ASIC1 | -0.675837077 |  | CCDC136 | 0.698704243 |
| SMC4 | -0.675202634 |  | PPP6C | 0.698178212 |
| CHM | -0.67482858 |  | FAM83G | 0.697783541 |
| SRR | -0.674428533 |  | ENDOD1 | 0.697727838 |
| LGALS3 | -0.674228753 |  | VMA21 | 0.697611204 |
| EDEM3 | -0.673903923 |  | CLPTM1L | 0.697481921 |
| PTGER4 | -0.673857772 |  | MATN3 | 0.69748185 |
| DAB2 | -0.673479943 |  | C3orf33 | 0.697350513 |
| DDX6 | -0.672524974 |  | LIG1 | 0.696928059 |
| TSPAN11 | -0.671728531 |  | H1F0 | 0.696926771 |
| ZNF3 | -0.671596232 |  | IL17RA | 0.696850101 |
| IGF2BP2 | -0.671545657 |  | DKK2 | 0.696700501 |
| PTPLAD1 | -0.670866754 |  | SLC17A9 | 0.696028916 |
| CXCR7 | -0.670722952 |  | COLEC11 | 0.695587927 |
| RUVBL1 | -0.670375987 |  | LPIN2 | 0.69541703 |
| GPR155 | -0.670358652 |  | PRELID2 | 0.695324896 |
| SPOCK1 | -0.670113198 |  | CPEB4 | 0.694421247 |
| LRRC34 | -0.669869827 |  | LGMN | 0.694136775 |
| GTF2B | -0.668755692 |  | ZRANB1 | 0.69364845 |
| GNB1L | -0.668623537 |  | ZNF207 | 0.693116708 |
| RGS17 | -0.66853791 |  | NYNRIN | 0.692662412 |
| CARF | -0.668269995 |  | HELLS | 0.692653897 |
| BUB3 | -0.667968326 |  | NDST2 | 0.692583416 |
| TATDN3 | -0.667634358 |  | SENP5 | 0.692415391 |
| FAM20A | -0.667552429 |  | MAOA | 0.691424376 |
| EPS15L1 | -0.667347315 |  | GPR176 | 0.691423134 |
| CNTRL | -0.666806242 |  | SIDT2 | 0.691300803 |
| ALG14 | -0.666466805 |  | SFXN3 | 0.690535504 |
| GPATCH4 | -0.666168405 |  | LINC00115 | 0.690143678 |
| IGFBP4 | -0.666121736 |  | TOM1L1 | 0.690143496 |
| UBLCP1 | -0.666057634 |  | TEK | 0.689625201 |
| SCRN3 | -0.665798279 |  | ITFG3 | 0.689500876 |
| FAM198B | -0.665742874 |  | KMT2A | 0.689301455 |
| DCAF4 | -0.665135011 |  | MGST2 | 0.689170669 |
| MBP | -0.664199266 |  | HAPLN1 | 0.689058109 |
| ELN | -0.663953868 |  | CTDSP2 | 0.68888116 |
| ASL | -0.663893683 |  | SYPL2 | 0.688343849 |
| HIVEP3 | -0.663594604 |  | RTN3 | 0.688123682 |
| NET1 | -0.662867715 |  | MAN1A2 | 0.687840324 |
| ZCCHC24 | -0.662816967 |  | SMCR8 | 0.687729306 |
| PITPNC1 | -0.66268708 |  | SLC38A9 | 0.687708826 |
| COQ7 | -0.662686308 |  | MFSD10 | 0.687217109 |
| MKLN1 | -0.662581558 |  | PHF10 | 0.687110791 |
| RBL1 | -0.662533347 |  | ALG5 | 0.686971116 |
| MRPL4 | -0.662420171 |  | RBM47 | 0.686391059 |
| PHYHD1 | -0.662348896 |  | HIPK3 | 0.686315575 |
| DHODH | -0.662012914 |  | ATP6V1D | 0.686093589 |
| POLR1E | -0.661529859 |  | ITPR1-AS1 | 0.685991462 |
| SERINC3 | -0.661427806 |  | MAPKAPK5-AS1 | 0.685896852 |
| PXMP4 | -0.661415478 |  | MR1 | 0.685882378 |
| LOC389831 | -0.661071339 |  | DNM2 | 0.684957453 |
| EXTL2 | -0.660807909 |  | LOC100996578 | 0.684784035 |
| MBD4 | -0.660652694 |  | ASXL1 | 0.684579564 |
| NUP54 | -0.660458805 |  | HLA-G | 0.68445346 |
| SGCB | -0.660201739 |  | NLRX1 | 0.684333312 |
| DAPK2 | -0.660013925 |  | GCLM | 0.683891018 |
| PUS1 | -0.659983822 |  | ASB13 | 0.683826151 |
| ANTXR2 | -0.659703392 |  | PPP6R2 | 0.683743162 |
| LTV1 | -0.659463934 |  | TRIM6 | 0.683691722 |
| HSPB1 | -0.659153347 |  | DDB2 | 0.683493394 |
| LARP4 | -0.659062653 |  | IFIT5 | 0.683367318 |
| PLCL1 | -0.65870648 |  | METTL9 | 0.683311355 |
| RFX7 | -0.658705931 |  | HEPH | 0.68293085 |
| GAPVD1 | -0.658083342 |  | TNFRSF10B | 0.682534735 |
| GSN | -0.658026358 |  | SREK1IP1 | 0.682335037 |
| RPS6KA5 | -0.657923636 |  | SLC37A1 | 0.682074585 |
| C21orf33 | -0.657774931 |  | HPS1 | 0.681852647 |
| ALAD | -0.657705376 |  | WDR54 | 0.681670268 |
| LINC00472 | -0.657688926 |  | PTP4A1 | 0.681549221 |
| RHOT1 | -0.657490236 |  | DEDD2 | 0.681466427 |
| BID | -0.657208737 |  | MMP24-AS1 | 0.681456412 |
| GEMIN2 | -0.657130888 |  | DDR1 | 0.681289974 |
| NAF1 | -0.656835758 |  | FEN1 | 0.681222274 |
| PEMT | -0.656781286 |  | RNF182 | 0.680800613 |
| C2orf42 | -0.655911812 |  | TMEM127 | 0.680604344 |
| WDR19 | -0.655571786 |  | JMJD8 | 0.68055179 |
| WIPF1 | -0.655197262 |  | BAG1 | 0.680390807 |
| CCSER2 | -0.655182479 |  | ATP6V0C | 0.680119901 |
| CEP68 | -0.654919359 |  | WWC3 | 0.680114939 |
| RHOJ | -0.654407177 |  | RTN2 | 0.679919735 |
| FAM161A | -0.654214989 |  | CREBZF | 0.679029507 |
| LMNA | -0.65343382 |  | KCNT2 | 0.67838032 |
| DZIP1 | -0.653310125 |  | TOMM34 | 0.677707092 |
| IKZF5 | -0.652769176 |  | B4GALT1 | 0.677630364 |
| SNX1 | -0.65210712 |  | DOCK10 | 0.677607839 |
| CWC25 | -0.651899553 |  | AGAP2-AS1 | 0.677410012 |
| BCCIP | -0.651895653 |  | LAPTM4B | 0.677090342 |
| ST3GAL1 | -0.651838632 |  | LOC100507291 | 0.677043697 |
| MBNL1 | -0.651716357 |  | TMEM50A | 0.676474745 |
| RFX5 | -0.65161289 |  | C9orf69 | 0.676027127 |
| ITGB8 | -0.651245389 |  | SSFA2 | 0.67593523 |
| AHNAK | -0.651193806 |  | UVSSA | 0.675745436 |
| HECTD1 | -0.650963889 |  | MBOAT1 | 0.67567784 |
| KNOP1 | -0.650751142 |  | C2orf27A | 0.675249403 |
| ENC1 | -0.649935267 |  | RTN1 | 0.674624082 |
| LZIC | -0.649896409 |  | SLC4A8 | 0.674434828 |
| TAF3 | -0.649850378 |  | CGA | 0.674273466 |
| CPSF3L | -0.649756715 |  | RPL28 | 0.674266372 |
| BBS4 | -0.649691691 |  | DCK | 0.674111301 |
| TMEM67 | -0.649515014 |  | GPX7 | 0.674080993 |
| USP53 | -0.649301664 |  | DIRC3 | 0.673828312 |
| SSC5D | -0.649261861 |  | SPG20OS | 0.673630054 |
| EYA1 | -0.648740324 |  | BMP2 | 0.673178799 |
| RGMA | -0.648494772 |  | PREX1 | 0.673031434 |
| C1orf21 | -0.648187196 |  | ALG8 | 0.672762877 |
| CCDC69 | -0.648180902 |  | TP53TG1 | 0.672694028 |
| SBF2 | -0.647998192 |  | KCTD11 | 0.672526471 |
| DCTN5 | -0.647745856 |  | EPM2AIP1 | 0.670864134 |
| MIS18BP1 | -0.64765165 |  | ZWILCH | 0.67073337 |
| IDH3G | -0.647258068 |  | SEMA4F | 0.670009638 |
| SIX4 | -0.646609559 |  | SUDS3 | 0.669813247 |
| GPRC5C | -0.646333784 |  | CNNM2 | 0.669557022 |
| IQCG | -0.646289086 |  | INTS6-AS1 | 0.669112921 |
| OGG1 | -0.646001242 |  | SDC4 | 0.668997971 |
| EFNA1 | -0.645430468 |  | ABCB5 | 0.668886497 |
| GSE1 | -0.645352969 |  | LAMP1 | 0.667818844 |
| ARHGEF40 | -0.644896616 |  | CCR10 | 0.66769511 |
| IL1RL1 | -0.644888517 |  | NGLY1 | 0.66760766 |
| LYRM7 | -0.644836946 |  | CHFR | 0.667225637 |
| SNRNP27 | -0.644409065 |  | POC5 | 0.667180712 |
| POLH | -0.644154268 |  | PDK3 | 0.667082665 |
| ABCA9 | -0.643938084 |  | RAB27B | 0.667015643 |
| MCL1 | -0.643670974 |  | HOXD-AS1 | 0.666186398 |
| RDH11 | -0.643482828 |  | ABRACL | 0.665890488 |
| ZC3H6 | -0.643460119 |  | VMP1 | 0.665854944 |
| C1GALT1 | -0.643114395 |  | TRPM4 | 0.665847634 |
| ADCY3 | -0.643090742 |  | YRDC | 0.665545538 |
| LARS | -0.642975022 |  | LMF2 | 0.665498269 |
| ASCC3 | -0.642931779 |  | TPGS2 | 0.665199891 |
| NBN | -0.642700196 |  | OSBPL3 | 0.664487167 |
| CTNNBIP1 | -0.642623854 |  | CDCP1 | 0.663783365 |
| SMARCE1 | -0.642212661 |  | NCOA3 | 0.663732384 |
| SLC25A23 | -0.641268409 |  | NCBP2-AS2 | 0.663662469 |
| ZYG11B | -0.641072765 |  | NUCB2 | 0.663551917 |
| OTUD3 | -0.640981336 |  | PERP | 0.663047308 |
| KTN1 | -0.640765031 |  | SDPR | 0.662958646 |
| CCDC8 | -0.640736131 |  | MAFK | 0.66273276 |
| SEMA3A | -0.640112686 |  | CDS2 | 0.662683694 |
| GTPBP6 | -0.639864929 |  | ST3GAL3 | 0.662649217 |
| IFRD2 | -0.639768362 |  | LINC00565 | 0.662563386 |
| DGAT2 | -0.639377356 |  | CD2AP | 0.662541983 |
| NOC2L | -0.638857087 |  | LNX1 | 0.662397894 |
| LDLRAP1 | -0.638509851 |  | TMEM38A | 0.661779601 |
| RPP40 | -0.638332893 |  | CMAS | 0.661713071 |
| ETV6 | -0.638168809 |  | SPPL2A | 0.661436813 |
| FBXO28 | -0.637054891 |  | CHPF | 0.66130707 |
| CDC6 | -0.637045264 |  | CASP4 | 0.661058984 |
| ACOX2 | -0.636505833 |  | IFI44 | 0.661031947 |
| TSHZ2 | -0.636078592 |  | SLC6A15 | 0.660978547 |
| PDE1C | -0.634096919 |  | C12orf75 | 0.660677376 |
| PRKG1 | -0.634035481 |  | CNOT6 | 0.66033397 |
| TMEM254 | -0.633938424 |  | SLC35D2 | 0.660218279 |
| ULK2 | -0.633798853 |  | FANCI | 0.660165455 |
| AHI1 | -0.633796516 |  | DDOST | 0.660086526 |
| PNO1 | -0.633418821 |  | TXNDC11 | 0.660024109 |
| HSPA4L | -0.633368851 |  | PKP4 | 0.660022119 |
| CAMK1D | -0.633140435 |  | TXNDC15 | 0.65995963 |
| RRN3 | -0.632826543 |  | RABIF | 0.659289917 |
| YES1 | -0.632802543 |  | TAF11 | 0.65917532 |
| ARNTL | -0.632661085 |  | ATP1A1 | 0.6586344 |
| CYP20A1 | -0.632476289 |  | ZCCHC2 | 0.658596784 |
| MAP3K2 | -0.6324197 |  | LOC100996615 | 0.658477858 |
| PLK1S1 | -0.632268204 |  | SPIN3 | 0.658063078 |
| CCDC126 | -0.632163475 |  | PITHD1 | 0.65779598 |
| SLC25A45 | -0.631875532 |  | TP53I13 | 0.657709658 |
| SNX6 | -0.631866167 |  | BTBD9 | 0.657676567 |
| TMEM237 | -0.631417954 |  | LOC100505715 | 0.657555324 |
| DDHD1 | -0.63125977 |  | PRKCH | 0.657526107 |
| GALNT15 | -0.631021521 |  | LOC100996920 | 0.65748352 |
| PDZD8 | -0.62998319 |  | CDC42 | 0.657479668 |
| PRKRIR | -0.629283792 |  | STX2 | 0.657157578 |
| TPMT | -0.628531252 |  | DST | 0.656860443 |
| USP46-AS1 | -0.628127918 |  | ANKRD36B | 0.656817541 |
| HEATR5B | -0.627741349 |  | SERPINE1 | 0.656730912 |
| MUM1 | -0.627308567 |  | C14orf132 | 0.656508215 |
| ITGA8 | -0.627248281 |  | ECT2 | 0.65631602 |
| TMEM150A | -0.626740885 |  | RER1 | 0.655956741 |
| ANAPC10 | -0.626734286 |  | TSKU | 0.655848304 |
| RING1 | -0.626640785 |  | SPTLC2 | 0.655543951 |
| TTLL12 | -0.626457142 |  | KIF9 | 0.655287218 |
| NIPSNAP1 | -0.62635145 |  | TMEM158 | 0.655242058 |
| ETV3 | -0.626242671 |  | KCTD2 | 0.654829202 |
| NIN | -0.626150797 |  | HTRA4 | 0.654793771 |
| UTRN | -0.625969708 |  | COQ4 | 0.654516698 |
| XIST | -0.625736213 |  | IP6K1 | 0.654087069 |
| SENP6 | -0.625208067 |  | PTGS2 | 0.653896445 |
| RPGRIP1L | -0.625147957 |  | HDLBP | 0.653650676 |
| ZNF532 | -0.6249548 |  | ICAM1 | 0.653573062 |
| ZNF703 | -0.62486574 |  | FZD6 | 0.653372077 |
| IL17RC | -0.624683499 |  | FBXO7 | 0.653201977 |
| NEDD9 | -0.624562967 |  | CHEK1 | 0.653185691 |
| RNMT | -0.624249766 |  | TTC32 | 0.652951805 |
| FNDC3A | -0.624110998 |  | MAFG | 0.652924505 |
| CENPW | -0.623874371 |  | NOV | 0.652905236 |
| ERV3-1 | -0.622837275 |  | KCNMB4 | 0.652862487 |
| SYNJ2BP | -0.622620472 |  | PTPN14 | 0.652813331 |
| CRADD | -0.622596695 |  | LOC151009 | 0.652720505 |
| MAPK8 | -0.622390789 |  | TXNRD2 | 0.652311603 |
| HPD | -0.621774486 |  | LAYN | 0.652008998 |
| ANXA6 | -0.62171825 |  | TRIM68 | 0.651964108 |
| WBP11 | -0.621389678 |  | TEP1 | 0.651888716 |
| BTG2 | -0.621267335 |  | ANKRD37 | 0.651841016 |
| OGFRL1 | -0.621162413 |  | SRPR | 0.651424873 |
| FOXN3 | -0.620966681 |  | RAB36 | 0.651352787 |
| PDE7B | -0.62095465 |  | NRCAM | 0.651313911 |
| DR1 | -0.620758508 |  | SNX7 | 0.651249839 |
| NOP16 | -0.620386051 |  | OASL | 0.651120938 |
| SRSF6 | -0.620167643 |  | REEP4 | 0.650771654 |
| SELO | -0.619964139 |  | SLC15A3 | 0.65074761 |
| KLF6 | -0.619865053 |  | TBC1D1 | 0.650533515 |
| COPS2 | -0.619782231 |  | EIF2B5 | 0.649971043 |
| UFL1 | -0.619177314 |  | GRSF1 | 0.649926363 |
| ADAT2 | -0.618805288 |  | PNMAL1 | 0.649306572 |
| IL1RN | -0.618578639 |  | HYI | 0.649139579 |
| ADIPOR1 | -0.618400986 |  | CCDC176 | 0.649020383 |
| MICU3 | -0.618251712 |  | SLC2A5 | 0.648700842 |
| BACH1 | -0.618070402 |  | LPPR2 | 0.648564495 |
| PPP3R1 | -0.617873543 |  | YBEY | 0.648326631 |
| FGF18 | -0.617780683 |  | FIG4 | 0.64832612 |
| FGFR1 | -0.617776711 |  | VAT1L | 0.6480931 |
| SPDL1 | -0.617748481 |  | DISP1 | 0.647945466 |
| ZNRF1 | -0.617394576 |  | SYN2 | 0.647722834 |
| PPAP2A | -0.617232462 |  | MOGS | 0.647679226 |
| FAM118B | -0.616584174 |  | RAB27A | 0.647135337 |
| SULF2 | -0.616366829 |  | GNS | 0.647030859 |
| SLC7A6 | -0.616024216 |  | WDTC1 | 0.646571125 |
| PLTP | -0.615849987 |  | PCED1B | 0.646156126 |
| KLHDC10 | -0.61577504 |  | PLOD2 | 0.646005249 |
| ZBTB7A | -0.615321002 |  | RNF14 | 0.645701744 |
| NAV1 | -0.614994015 |  | CFL2 | 0.645551093 |
| SUV39H2 | -0.614827223 |  | IGFBP3 | 0.645535381 |
| CLASP1 | -0.614597575 |  | REN | 0.645236189 |
| ZDHHC3 | -0.613745816 |  | TMCO1 | 0.645234585 |
| FKBP4 | -0.613564273 |  | AHRR | 0.645129703 |
| OSBPL1A | -0.61345192 |  | DENND1A | 0.645018494 |
| DDX20 | -0.613393413 |  | TMEM223 | 0.644967478 |
| ARMC8 | -0.613203457 |  | RHNO1 | 0.644578781 |
| LOC100996555 | -0.613099049 |  | ERBB2 | 0.644552917 |
| WSB1 | -0.612760675 |  | SNCA | 0.644176886 |
| TMEM168 | -0.611466398 |  | ANKRD10 | 0.644045589 |
| CABLES1 | -0.611334001 |  | ZNF346 | 0.643647736 |
| PPM1A | -0.611281662 |  | CCDC104 | 0.643393888 |
| UBXN2B | -0.611076409 |  | ERV3-2 | 0.643325807 |
| METTL20 | -0.610940526 |  | SSR3 | 0.642801215 |
| PTK7 | -0.610866132 |  | KIAA1737 | 0.642516908 |
| KIRREL | -0.610865586 |  | LOC283508 | 0.642356112 |
| AKAP13 | -0.610554967 |  | PRPF38A | 0.642014568 |
| RPL22L1 | -0.610429743 |  | C15orf65 | 0.641665364 |
| PRKG2 | -0.610154661 |  | CTSB | 0.641493275 |
| EMCN | -0.609715674 |  | NPC1 | 0.641315697 |
| ALG10B | -0.609635688 |  | RRM1 | 0.641276442 |
| SOX5 | -0.609465844 |  | NAPG | 0.6408743 |
| TCF7L1 | -0.609403252 |  | ASPHD1 | 0.640591696 |
| DICER1 | -0.60899027 |  | GORASP2 | 0.639757992 |
| REV3L | -0.608888868 |  | RTP4 | 0.639252938 |
| CWC22 | -0.608684116 |  | ARRB1 | 0.63903827 |
| ZNF37A | -0.608448474 |  | TVP23B | 0.639005291 |
| ANGEL2 | -0.608094804 |  | RNF149 | 0.638785324 |
| INHBA | -0.607975344 |  | IDS | 0.638525812 |
| EFR3A | -0.607963426 |  | HMGA1 | 0.638084267 |
| METTL1 | -0.607836114 |  | GLIS2 | 0.63807027 |
| SNUPN | -0.607593135 |  | ZNF702P | 0.637497611 |
| RASSF8 | -0.607577178 |  | IL1A | 0.637215102 |
| PPM1B | -0.607284607 |  | C6orf132 | 0.636845138 |
| FAM217B | -0.607214942 |  | NAGLU | 0.636191968 |
| FIP1L1 | -0.60716782 |  | HIST1H2AJ | 0.636047185 |
| EIF2S2 | -0.607078763 |  | TRIM13 | 0.635403025 |
| DUS3L | -0.607070576 |  | GBP3 | 0.634482115 |
| CNIH3 | -0.606157956 |  | NINJ2 | 0.634473114 |
| SNAI3-AS1 | -0.605914835 |  | RAB3GAP1 | 0.634133824 |
| MCRS1 | -0.605812524 |  | HOXB2 | 0.633876181 |
| RASAL2 | -0.60520886 |  | C15orf37 | 0.63378578 |
| RBBP8 | -0.604620245 |  | EREG | 0.633611706 |
| TCHP | -0.604594117 |  | NAP1L3 | 0.633409225 |
| WDR4 | -0.604407487 |  | PHF11 | 0.633355776 |
| LRRC27 | -0.604401286 |  | CCL20 | 0.633153554 |
| FERMT2 | -0.603608895 |  | C3orf70 | 0.633109173 |
| LOC100287896 | -0.603388076 |  | GALNS | 0.633098648 |
| MAP1LC3A | -0.603274526 |  | NF2 | 0.633039671 |
| PRKCE | -0.603260538 |  | SLC48A1 | 0.632732896 |
| BTBD19 | -0.602869189 |  | LTBP1 | 0.632383331 |
| DMKN | -0.602590269 |  | TGFBRAP1 | 0.631760113 |
| GEMIN8 | -0.602584255 |  | RASA2 | 0.631584834 |
| SMARCC1 | -0.602379301 |  | ZNF404 | 0.63135818 |
| IFI16 | -0.602342451 |  | BTG3 | 0.631281095 |
| SLAIN2 | -0.602340516 |  | RNF7 | 0.631096902 |
| PDE4D | -0.602255066 |  | HGSNAT | 0.630995148 |
| PDS5A | -0.601744886 |  | CCDC71L | 0.63033926 |
| LOC374443 | -0.601627199 |  | NBEA | 0.630308042 |
| FGFRL1 | -0.601411864 |  | ORAI2 | 0.630241426 |
| USP40 | -0.601308904 |  | GPBP1L1 | 0.630156925 |
| UGGT2 | -0.601207135 |  | SULT1E1 | 0.630153893 |
| SAT1 | -0.601158787 |  | HIST1H2AM | 0.630096098 |
| FOXP1 | -0.600862528 |  | ZKSCAN5 | 0.629861072 |
| EVL | -0.600830113 |  | CRYBB2P1 | 0.629690292 |
| CD109 | -0.600680058 |  | ZBED6CL | 0.629688529 |
| SAR1B | -0.600600923 |  | PGK1 | 0.629201353 |
| BBIP1 | -0.600394011 |  | BIRC6 | 0.629127389 |
| SLC30A1 | -0.600330102 |  | CLOCK | 0.628229293 |
| HNRNPDL | -0.600023949 |  | MGAT1 | 0.628222218 |
| IDH3A | -0.599853061 |  | CEP152 | 0.628078292 |
| RAB11B-AS1 | -0.599828769 |  | PSMF1 | 0.627855927 |
| TRPS1 | -0.599520862 |  | BCR | 0.62779429 |
| N4BP2 | -0.599195322 |  | PPFIA4 | 0.627244216 |
| UBE2D1 | -0.598930624 |  | LOXL3 | 0.627048828 |
| KIAA0586 | -0.598624985 |  | DIAPH3 | 0.626954597 |
| SLC16A7 | -0.598237287 |  | ZNF395 | 0.626932454 |
| MYO1E | -0.597902868 |  | FAM120C | 0.626459762 |
| PGBD4 | -0.597660734 |  | PCF11 | 0.626429707 |
| ITIH1 | -0.597658733 |  | HIST1H2BD | 0.626378648 |
| MT1F | -0.597613103 |  | B4GALT4 | 0.626239232 |
| CDK1 | -0.597429347 |  | IFI35 | 0.626231959 |
| SEPT2 | -0.596995379 |  | HOXA10 | 0.625916947 |
| FOXF1 | -0.596739847 |  | NEGR1 | 0.62567717 |
| MOCOS | -0.596302735 |  | APH1A | 0.625452975 |
| NME6 | -0.596214795 |  | TRIM27 | 0.625198466 |
| RBM41 | -0.595913523 |  | EHD1 | 0.624920494 |
| SVEP1 | -0.595669272 |  | FAM46A | 0.624767287 |
| CADPS | -0.595666722 |  | STIM2 | 0.624764154 |
| TEX9 | -0.595453939 |  | SPTSSA | 0.624687198 |
| CTDSPL2 | -0.595387084 |  | TBC1D24 | 0.624258573 |
| GSTZ1 | -0.595260726 |  | KDELR1 | 0.624178562 |
| NARS | -0.595095192 |  | RBBP6 | 0.624157764 |
| CEP44 | -0.59484489 |  | ALG9 | 0.624132129 |
| CORO6 | -0.594804162 |  | PCSK5 | 0.62382536 |
| YTHDC2 | -0.594545386 |  | POMT2 | 0.623544631 |
| ACTR8 | -0.594399152 |  | LYPLA2 | 0.623370683 |
| ZNF682 | -0.594074678 |  | MMP15 | 0.623031665 |
| PKI55 | -0.593960357 |  | TRIM52 | 0.622754676 |
| CXCL12 | -0.593478609 |  | POFUT2 | 0.622543891 |
| FUT8 | -0.593022962 |  | RPL31 | 0.622244254 |
| PCYOX1 | -0.592524356 |  | LOC286068 | 0.622244002 |
| NUP160 | -0.592232922 |  | C6orf226 | 0.62205799 |
| STK17B | -0.591987769 |  | FOXRED2 | 0.621750931 |
| SFXN2 | -0.591653791 |  | TLDC1 | 0.621734945 |
| UBE3A | -0.591577206 |  | SLC35B3 | 0.621127014 |
| PDE10A | -0.590328297 |  | ZNF587 | 0.621112961 |
| CALD1 | -0.590072955 |  | COQ10B | 0.621068671 |
| AKAP12 | -0.590023035 |  | ZNF776 | 0.62051028 |
| COX20 | -0.589959988 |  | PLOD3 | 0.62016449 |
| CNOT7 | -0.589852889 |  | LRBA | 0.620151376 |
| WHSC1L1 | -0.589695252 |  | EMC1 | 0.619843782 |
| EDC3 | -0.589415977 |  | ECHDC2 | 0.619806296 |
| PLIN5 | -0.589379138 |  | FLJ31715 | 0.619311692 |
| TRMT13 | -0.589213593 |  | RCHY1 | 0.619172641 |
| ADAL | -0.589177235 |  | LINC00674 | 0.618789067 |
| SUMO4 | -0.588941744 |  | B4GALT3 | 0.618709247 |
| CDC42EP3 | -0.588751595 |  | FLG-AS1 | 0.618628872 |
| C14orf37 | -0.588610742 |  | TOMM20 | 0.618435758 |
| ADPGK | -0.588207252 |  | ZNF837 | 0.618374632 |
| SRGN | -0.588144195 |  | EIF5A2 | 0.618099863 |
| MCTP1 | -0.587720542 |  | MGAT4B | 0.617810677 |
| DCAF5 | -0.587706156 |  | UCHL1 | 0.617790105 |
| ACTR3 | -0.587584745 |  | GPR160 | 0.616388287 |
| ADCY1 | -0.587235014 |  | HIST1H2AK | 0.616109835 |
| RRAS2 | -0.587038836 |  | AURKA | 0.61557686 |
| ZDHHC7 | -0.586863172 |  | XAF1 | 0.615430645 |
| SFRP1 | -0.586782488 |  | NCOA1 | 0.614816295 |
| SDAD1 | -0.586604683 |  | MXRA7 | 0.614791942 |
| YWHAZ | -0.586174263 |  | SLC22A18 | 0.61474961 |
| HMGB3 | -0.585924867 |  | ALG2 | 0.614737975 |
| STAM2 | -0.585825745 |  | ZC2HC1C | 0.614635469 |
| NAT9 | -0.585087787 |  | RSAD2 | 0.614556531 |
|  |  |  | SLFN12 | 0.614463207 |
|  |  |  | NBR2 | 0.6142312 |
|  |  |  | GPR133 | 0.613842953 |
|  |  |  | NEDD4L | 0.613692721 |
|  |  |  | IFI27L2 | 0.613647946 |
|  |  |  | SMIM3 | 0.613618342 |
|  |  |  | SLC35C1 | 0.613519315 |
|  |  |  | SP3 | 0.613423618 |
|  |  |  | TRAF3IP2-AS1 | 0.61316279 |
|  |  |  | FLJ43663 | 0.613089465 |
|  |  |  | ERLEC1 | 0.613045647 |
|  |  |  | TMEM246 | 0.61223223 |
|  |  |  | ZNF512 | 0.612224036 |
|  |  |  | MRPL23 | 0.612116501 |
|  |  |  | ZNF708 | 0.611976413 |
|  |  |  | ADM | 0.611915636 |
|  |  |  | METRN | 0.611798889 |
|  |  |  | CAHM | 0.611770055 |
|  |  |  | NAA40 | 0.611497912 |
|  |  |  | LOXL2 | 0.611447387 |
|  |  |  | ARRDC3-AS1 | 0.611202279 |
|  |  |  | FBXL14 | 0.611194367 |
|  |  |  | YTHDF3-AS1 | 0.61098827 |
|  |  |  | SGSH | 0.6103196 |
|  |  |  | LCAT | 0.610296049 |
|  |  |  | MAMDC4 | 0.610200487 |
|  |  |  | KMT2D | 0.610153694 |
|  |  |  | RNF114 | 0.610043621 |
|  |  |  | COPZ2 | 0.609590111 |
|  |  |  | PAGR1 | 0.609512618 |
|  |  |  | ATP6V1G2 | 0.609465886 |
|  |  |  | BMP1 | 0.609291769 |
|  |  |  | ZCCHC10 | 0.609283939 |
|  |  |  | TMED8 | 0.609203738 |
|  |  |  | NDUFB2 | 0.609121368 |
|  |  |  | HSD17B14 | 0.608984219 |
|  |  |  | LINC00960 | 0.608900087 |
|  |  |  | CDIP1 | 0.608789439 |
|  |  |  | PPME1 | 0.608763027 |
|  |  |  | ATXN7L3B | 0.608664702 |
|  |  |  | ACER3 | 0.608409789 |
|  |  |  | SZT2 | 0.608345632 |
|  |  |  | CADM1 | 0.608151744 |
|  |  |  | SLC20A2 | 0.60792673 |
|  |  |  | HOPX | 0.607922508 |
|  |  |  | ZNF664 | 0.607787887 |
|  |  |  | LRRC8E | 0.607787694 |
|  |  |  | SLC35B4 | 0.607569288 |
|  |  |  | PPTC7 | 0.606920646 |
|  |  |  | LOC646762 | 0.606590207 |
|  |  |  | ZNF264 | 0.606291097 |
|  |  |  | TUSC1 | 0.606257414 |
|  |  |  | FBXL17 | 0.605946621 |
|  |  |  | SUMF1 | 0.605528134 |
|  |  |  | TUSC3 | 0.605025683 |
|  |  |  | MFAP3 | 0.605018602 |
|  |  |  | STC1 | 0.604862444 |
|  |  |  | ATF3 | 0.604789983 |
|  |  |  | SLIT2 | 0.604032572 |
|  |  |  | PPM1F | 0.603991135 |
|  |  |  | ILF3-AS1 | 0.603608806 |
|  |  |  | NIM1 | 0.603526614 |
|  |  |  | SLC1A1 | 0.603447431 |
|  |  |  | SNX11 | 0.603360282 |
|  |  |  | AP2B1 | 0.603160378 |
|  |  |  | LOC401321 | 0.603030923 |
|  |  |  | RAB3B | 0.602948023 |
|  |  |  | ZBED4 | 0.60264516 |
|  |  |  | ANO4 | 0.602353377 |
|  |  |  | CMAHP | 0.602241336 |
|  |  |  | RNASEH2B | 0.601883712 |
|  |  |  | HOXB5 | 0.60187156 |
|  |  |  | EFCAB7 | 0.601813326 |
|  |  |  | ATG5 | 0.601721215 |
|  |  |  | TBL1X | 0.601546283 |
|  |  |  | TMEM217 | 0.601083994 |
|  |  |  | LOC80154 | 0.601073338 |
|  |  |  | APOL3 | 0.601003611 |
|  |  |  | TMEM134 | 0.600954948 |
|  |  |  | KLHL42 | 0.600824869 |
|  |  |  | SAV1 | 0.600708044 |
|  |  |  | WTIP | 0.600440903 |
|  |  |  | BBOX1 | 0.600118122 |
|  |  |  | PRRG4 | 0.599996064 |
|  |  |  | UBN2 | 0.599935749 |
|  |  |  | TECPR2 | 0.599926195 |
|  |  |  | L3MBTL4 | 0.599725842 |
|  |  |  | CACFD1 | 0.599691301 |
|  |  |  | LOC100506299 | 0.599597927 |
|  |  |  | PSD3 | 0.599470101 |
|  |  |  | RHBDF1 | 0.599461462 |
|  |  |  | CXCL16 | 0.59936577 |
|  |  |  | NAPB | 0.599219518 |
|  |  |  | PDCD6 | 0.598660938 |
|  |  |  | RAD51D | 0.598461079 |
|  |  |  | POC1A | 0.598249543 |
|  |  |  | CUL4A | 0.598123431 |
|  |  |  | SLC25A30 | 0.59811644 |
|  |  |  | SLC15A4 | 0.597941353 |
|  |  |  | PRRG1 | 0.597635562 |
|  |  |  | SH2D5 | 0.597617568 |
|  |  |  | CCK | 0.597401327 |
|  |  |  | ARHGEF28 | 0.59728045 |
|  |  |  | RP2 | 0.597254118 |
|  |  |  | PRPS1 | 0.597230659 |
|  |  |  | VPS36 | 0.597212521 |
|  |  |  | REM2 | 0.597200247 |
|  |  |  | PTPRB | 0.59716895 |
|  |  |  | TMEM173 | 0.596737822 |
|  |  |  | AGO2 | 0.596420557 |
|  |  |  | CERCAM | 0.596419622 |
|  |  |  | LOC100130219 | 0.596395016 |
|  |  |  | GDPD1 | 0.595869069 |
|  |  |  | B3GALT2 | 0.595760901 |
|  |  |  | PMM2 | 0.595740244 |
|  |  |  | ZBTB44 | 0.595716435 |
|  |  |  | ALG12 | 0.595314629 |
|  |  |  | SCAP | 0.594936029 |
|  |  |  | PPP1R14C | 0.594824323 |
|  |  |  | LOC339535 | 0.594347268 |
|  |  |  | IMPAD1 | 0.594183805 |
|  |  |  | EPHB1 | 0.59396474 |
|  |  |  | STARD3NL | 0.593455535 |
|  |  |  | CEP89 | 0.593400177 |
|  |  |  | TPD52 | 0.593286849 |
|  |  |  | A1BG | 0.593130252 |
|  |  |  | SPINT2 | 0.592736719 |
|  |  |  | RAB3IP | 0.592733142 |
|  |  |  | RIOK3 | 0.592577953 |
|  |  |  | ZBTB8OS | 0.592467014 |
|  |  |  | FANCD2 | 0.59229652 |
|  |  |  | ACTL10 | 0.591999529 |
|  |  |  | GNB5 | 0.591818465 |
|  |  |  | MTA3 | 0.591811373 |
|  |  |  | ZNF322 | 0.591760104 |
|  |  |  | LOC100507580 | 0.59117879 |
|  |  |  | DHRS3 | 0.590803568 |
|  |  |  | AEN | 0.589994898 |
|  |  |  | MAN1B1 | 0.589975067 |
|  |  |  | TRADD | 0.589638416 |
|  |  |  | SNORD89 | 0.589589835 |
|  |  |  | FSD1L | 0.589158237 |
|  |  |  | SLC39A7 | 0.588918744 |
|  |  |  | RARS2 | 0.588898339 |
|  |  |  | SLC35E3 | 0.588818199 |
|  |  |  | TRAPPC1 | 0.588595202 |
|  |  |  | STARD13 | 0.588561421 |
|  |  |  | FLCN | 0.588535185 |
|  |  |  | APAF1 | 0.58823761 |
|  |  |  | ST3GAL2 | 0.588066194 |
|  |  |  | ZNF788 | 0.588017192 |
|  |  |  | GOSR2 | 0.587955807 |
|  |  |  | HSBP1L1 | 0.587946335 |
|  |  |  | TENM3 | 0.587408539 |
|  |  |  | FAM69A | 0.586326964 |
|  |  |  | FAM219A | 0.58623936 |
|  |  |  | SLC44A2 | 0.585817136 |
|  |  |  | TNS3 | 0.585789811 |
|  |  |  | PIK3C2B | 0.585773896 |
|  |  |  | IER3IP1 | 0.585664297 |
|  |  |  | USP25 | 0.585597925 |
|  |  |  | SLC39A6 | 0.585415133 |
