# Supplementary material for: MYC Is an Early Response Regulator of Human Adipogenesis in Adipose Stem Cells
Source: PLoS One. 2014 Dec 1;9(12):e114133. doi: 10.1371/journal.pone.0114133 (PMC4250176; doi:10.1371/journal.pone.0114133)
Supplement: Table S3 — Gene ontology enrichment for pooled MYC siRNA significant genes. (DOCX) [file pone.0114133.s006.docx]

**MYC Pooled_Upregulated Pathways**

| Name | FDR p-value | Percent Identifiers | Number of Identifiers |
| --- | --- | --- | --- |
| Development | 6.17E-10 | 9.9 | 128 |
| Cell adhesion | 6.17E-10 | 13.8 | 67 |
| Chemokines and adhesion | 7.41E-08 | 20.1 | 31 |
| Regulation of epithelial-to-mesenchymal transition (EMT) | 7.79E-06 | 22.5 | 20 |
| Integrin outside-in signaling | 1.34E-05 | 23.7 | 18 |
| TGF-beta-dependent induction of EMT via RhoA, PI3K and ILK. | 1.42E-05 | 27.8 | 15 |
| ECM remodeling | 5.75E-05 | 25.0 | 15 |
| Endothelial cell contacts by non-junctional mechanisms | 8.43E-05 | 30.0 | 12 |
| TGF, WNT and cytoskeletal remodeling | 1.42E-04 | 15.0 | 27 |
| Stellate cells activation and liver fibrosis | 1.42E-04 | 19.8 | 18 |
| HGF signaling pathway | 1.44E-04 | 26.0 | 13 |
| Correlations from Replication data | 4.65E-04 | 19.8 | 16 |
| CD40 signaling | 6.74E-04 | 21.2 | 14 |
| IL-17 signaling pathways | 1.53E-03 | 19.7 | 14 |
| Role of tetraspanins in the integrin-mediated cell adhesion | 2.23E-03 | 21.4 | 12 |
| Plasmin signaling | 2.67E-03 | 22.4 | 11 |
| Cytoskeleton remodeling | 2.67E-03 | 10.1 | 48 |
| Influence of Ras and Rho proteins on G1/S Transition | 2.83E-03 | 19.4 | 13 |
| VEGF signaling via VEGFR2 - generic cascades | 2.84E-03 | 16.7 | 16 |
| Gamma-Secretase regulation of neuronal cell development and function | 3.57E-03 | 23.3 | 10 |
| BMP signaling | 4.25E-03 | 25.0 | 9 |
| Gastrin in inflammatory response | 4.48E-03 | 18.3 | 13 |
| PTEN pathway | 4.97E-03 | 20.4 | 11 |
| TREM1 signaling pathway | 4.97E-03 | 19.0 | 12 |

**MYC Pooled_Downregulated Pathways**

| Name | FDR p-value | Percent Identifiers | Number of Identifiers |
| --- | --- | --- | --- |
| Role of Diethylhexyl Phthalate and Tributyltin in fat cell differentiation | 1.28E-06 | 57.9 | 11 |
| SCAP/SREBP Transcriptional Control of Cholesterol and FA Biosynthesis | 1.85E-06 | 34.0 | 16 |
| Putative pathways for stimulation of fat cell differentiation by Bisphenol A | 3.15E-06 | 40.6 | 13 |
| Glycine, serine, cysteine and threonine metabolism | 2.19E-05 | 31.8 | 14 |
| Phenylalanine metabolism | 3.15E-05 | 39.3 | 11 |
| Insulin regulation of fatty acid methabolism | 5.01E-05 | 27.3 | 15 |
| Propionate metabolism p.2 | 1.19E-04 | 42.9 | 9 |
| Cholesterol Biosynthesis | 1.19E-04 | 42.9 | 9 |
| Tyrosine metabolism p.1 (dopamine) | 3.02E-04 | 44.4 | 8 |
| Catecholamine metabolism | 4.29E-04 | 42.1 | 8 |
| Tyrosine metabolism p.2 (melanin) | 4.87E-04 | 36.0 | 9 |
| Butanoate metabolism | 6.54E-04 | 34.6 | 9 |
| Aminoacyl-tRNA biosynthesis in cytoplasm | 1.27E-03 | 1.3 | 4 |
| Regulation of metabolism | 2.78E-03 | 18.2 | 16 |
| Tryptophan metabolism | 2.90E-03 | 28.1 | 9 |
| CoA biosynthesis | 2.90E-03 | 36.8 | 7 |
| Bile acids regulation of glucose and lipid metabolism via FXR | 4.73E-03 | 26.5 | 9 |
