# Supplementary material for: MYC Is an Early Response Regulator of Human Adipogenesis in Adipose Stem Cells
Source: PLoS One. 2014 Dec 1;9(12):e114133. doi: 10.1371/journal.pone.0114133 (PMC4250176; doi:10.1371/journal.pone.0114133)
Supplement: Table S4 — Gene ontology enrichment for s91-29 MYC siRNA significant genes. (DOCX) [file pone.0114133.s007.docx]

**MYC s91-29_Upregulated Pathways**

| Name | FDR p-value | Percent Identifiers | Number of Identifiers |
| --- | --- | --- | --- |
| Immune response | 8.95E-04 | 11.5 | 123 |
| N-Glycan biosynthesis p2 | 1.40E-03 | 45.0 | 9 |
| Sphingolipid metabolism | 1.53E-03 | 33.3 | 12 |
| Sphingolipid metabolism / Human version | 1.81E-03 | 32.4 | 12 |
| Neurophysiological process | 1.48E-02 | 4.7 | 39 |
| CD40 signaling | 1.48E-02 | 22.7 | 15 |
| Phospholipid metabolism p.1 | 3.35E-02 | 42.9 | 6 |
| ECM remodeling | 4.79E-02 | 21.7 | 13 |
| Th17 cytokines in COPD | 4.79E-02 | 20.6 | 14 |
| Vitamin B7 (biotin) metabolism | 4.85E-02 | 27.3 | 9 |

**MYC s91-29_Downregulated Pathways**

| Name | FDR p-value | Percent Identifiers | Number of Identifiers |
| --- | --- | --- | --- |
| Aminoacyl-tRNA biosynthesis in cytoplasm | 5.10E-07 | 1.0 | 3 |
| Olfactory transduction | 5.10E-07 | 0.9 | 3 |
| Aminoacyl-tRNA biosynthesis in cytoplasm/ Rodent version | 5.10E-07 | 1.0 | 3 |
| SCAP/SREBP Transcriptional Control of Cholesterol and FA Biosynthesis | 3.42E-05 | 36.2 | 17 |
| Development | 1.88E-04 | 12.2 | 158 |
| Growth hormone signaling via STATs and PLC/IP3 | 4.53E-04 | 40.0 | 12 |
| Role of Diethylhexyl Phthalate and Tributyltin in fat cell differentiation | 1.31E-03 | 47.4 | 9 |
| FAS signaling cascades | 5.99E-03 | 29.5 | 13 |
| GnRH signaling | 1.61E-02 | 21.3 | 19 |
| Cholesterol Biosynthesis | 1.86E-02 | 38.1 | 8 |
| Endothelin-1/EDNRA signaling | 1.87E-02 | 22.9 | 16 |
| PDGF signaling via MAPK cascades | 2.00E-02 | 26.7 | 12 |
| Cytoskeleton remodeling | 2.00E-02 | 13.5 | 64 |
| Normal and pathological TGF-beta-mediated regulation of cell proliferation | 2.00E-02 | 28.2 | 11 |
| Thyroliberin in cytoskeleton remodeling | 2.53E-02 | 28.6 | 10 |
| G-protein signaling | 2.53E-02 | 13.8 | 54 |
| Regulation of lipid metabolism | 2.53E-02 | 14.6 | 44 |
| Reproduction | 2.53E-02 | 18.4 | 23 |
| Insulin regulation of fatty acid methabolism | 2.87E-02 | 23.6 | 13 |
| Oncostatin M signaling via JAK-Stat in human cells | 2.94E-02 | 36.8 | 7 |
| Transport | 3.18E-02 | 13.1 | 61 |
| ATP metabolism | 3.26E-02 | 22.8 | 13 |
| Gastrin in cell growth and proliferation | 3.26E-02 | 21.9 | 14 |
| Membrane-bound ESR1: interaction with G-proteins signaling | 3.26E-02 | 21.1 | 15 |
| Gamma-aminobutyrate (GABA) biosynthesis and metabolism | 3.38E-02 | 35.0 | 7 |
| Putative pathways for stimulation of fat cell differentiation by Bisphenol A | 3.72E-02 | 28.1 | 9 |
| Propionate metabolism p.2 | 3.98E-02 | 33.3 | 7 |
| Arginine metabolism/ Rodent version | 3.98E-02 | 25.6 | 10 |
| Netrin-1 in regulation of axon guidance | 3.98E-02 | 23.1 | 12 |
